# Antioxidant and Anticancer Activity of Novel Derivatives of 3-[(4-Methoxyphenyl)amino]propanehydrazide

**DOI:** 10.3390/molecules25132980

**Published:** 2020-06-29

**Authors:** Ingrida Tumosienė, Kristina Kantminienė, Arnas Klevinskas, Vilma Petrikaitė, Ilona Jonuškienė, Vytautas Mickevičius

**Affiliations:** 1 Department of Organic Chemistry, Kaunas University of Technology, Radvilėnų pl. 19, LT-50254 Kaunas, Lithuania; ingrida.tumosiene@ktu.lt (I.T.); arnas.klevinskas@ktu.edu (A.K.); ilona.jonuskiene@ktu.lt (I.J.); vytautas.mickevicius@ktu.lt (V.M.); 2 Department of Physical and Inorganic Chemistry, Kaunas University of Technology, Radvilėnų pl. 19, LT-50254 Kaunas, Lithuania; 3 Laboratory of Drug Targets Histopathology, Institute of Cardiology, Lithuanian University of Health Sciences, Sukilėlių pr. 13, LT-50162 Kaunas, Lithuania; vilmapetrikaite@gmail.com; 4 Institute of Physiology and Pharmacology, Faculty of Medicine, Lithuanian University of Health Sciences, A. Mickevičiaus g. 9, LT-44307 Kaunas, Lithuania; 5 Institute of Biotechnology, Life Sciences Center, Vilnius University, Saulėtekio al. 7, LT-10257 Vilnius, Lithuania

**Keywords:** hydrazine, Schiff base, 1,2,4-triazole-3-thione, antioxidative, anticancer

## Abstract

Series of novel 3-[(4-methoxyphenyl)amino]propanehydrazide derivatives bearing semicarbazide, thiosemicarbazide, thiadiazole, triazolone, triazolethione, thiophenyltriazole, furan, thiophene, naphthalene, pyrrole, isoindoline-1,3-dione, oxindole, etc. moieties were synthesized and their molecular structures were confirmed by IR, ^1^H-, ^13^C-NMR spectroscopy and mass spectrometry data. The antioxidant activity of the synthesized compounds was screened by DPPH radical scavenging method. The antioxidant activity of *N*-(1,3-dioxoisoindolin-2-yl)-3-((4-methoxyphenyl)amino)propanamide and 3-((4-methoxyphenyl)amino)-*N’*-(1-(naphthalen-1-yl)-ethylidene)propanehydrazide has been tested to be *ca*. 1.4 times higher than that of a well-known antioxidant ascorbic acid. Anticancer activity was tested by MTT assay against human glioblastoma U-87 and triple-negative breast cancer MDA-MB-231 cell lines. In general, the tested compounds were more cytotoxic against U-87 than MDA-MB-231 cell line. 1-(4-Fluorophenyl)-2-((5-(2-((4-methoxyphenyl)amino)ethyl)-4-phenyl-4*H*-1,2,4-triazol-3-yl)thio)ethanone has been identified as the most active compound against the glioblastoma U-87 cell line.

## 1. Introduction

Oxidative stress, induced by the generation of reactive oxygen (ROS) and nitrogen (NOS) species, is considered a major causative factor of many contemporary diseases including diabetes, cardiovascular diseases, cancer, viral infection, several neurodegenerative diseases, and various digestive disorders [1]. Antioxidants are involved in the defense mechanism of the organism against pathologies associated with the attack of free radicals by slowing down or inhibiting completely the oxidation processes caused by reactive radicals. In recent years, the interest in synthesis and pharmacological properties of new antioxidant agents has been increasing rapidly. Several classes of organic compounds have attracted attention of the researchers as potential scaffolds for the synthesis of novel biologically active compounds.

1,2,4-Triazole derived compounds usually exhibit a series of pharmacological properties such as anticancer, antimicrobial, antiviral, anticonvulsant, antidepressant, antihypertensive, etc. [2,3,4,5]. 1,2,4-Triazole moiety can influence lipophilicity, polarity, and hydrogen bonding capacity of a molecule, improving pharmacological, pharmacokinetic, toxicological, and physicochemical properties of the compounds [5]. Several drugs, such as ribavirin, estazolam, triazolam, and alprazolam contain the 1,2,4-triazole moiety. Among the biologically active 1,2,4-triazole derivatives, the ones bearing 1,2,4-triazole-3-thione moiety distinguish themselves by a diverse spectrum of activities, including antioxidant, anticancer, antibacterial, and antiviral [6].

Hydrazone derivatives constitute another significant category of compounds in medicinal and pharmaceutical chemistry. It has been established that the biological activity of hydrazone compounds is associated with the presence of the active azomethine -NH-N=CH- pharmacophore [7] and these compounds, in combination with various heterocyclic scaffolds, possess diverse biological activity, including anticancer, antibacterial, antiviral, antiplatelet [8,9], as well as antioxidant one [10,11]. As a model of a hybrid drug, bearing hydrazone and thiophene moieties, 2,5-dimethoxy-terephthalaldehyde bis(thiophene-2-carbonylhydrazone) was synthesized by the condensation reaction between 2,5-dimethoxyterephthalaldehyde and 2-thiophenecarboxylic acid hydrazide to be evaluated by computational pharmacological evaluation for the drug’s pharmacokinetics in the human body and presented a drug score of 45% according to the Lipinski rule of five [12]. Phthalimide ring (isoindoline-1,3-dione) represents another promising pharmacophore subunit for incorporation into hydrazone molecule. The hydrophobic nature of phthalimides increases their potential to cross different biological membranes in vivo [13]. Several isoindoline-1,3-dione and 2-oxindole moieties-containing drugs have been reported as potent anticancer agents in advanced stages of development: RG-108 as DNA methyltransferase inhibitor and promising anticancer agent; orantinib as inhibitor of vascular endothelial growth factor receptors type 2 (VEGFR2), platelet-derived growth factor receptors (PDGFR), and platelet-derived growth factor receptors (FGFR) inhibitor, as well as agent effective against hepatocellular carcinoma; 16PF-00562271 as an inhibitor of focal adhesion kinase (FAK) and protein tyrosine kinase 2 (PYK2) and effective agent against hepatocellular carcinoma. [14]. Introduction of one or several electron donating methoxy groups into benzene ring has been proven to strengthen the anticancer activity of various compounds [15,16]. Incorporation of methoxybenzene and naphthalene moieties into the structure of dihydropyrazole isatin dihydrothiazole hybrid resulted in a very efficient derivative, exhibiting anticancer activity higher than currently used anticancer drug sunitinib [17].

As a continuation of our interest to further explore the structure-activity relationship of the biologically active derivatives of amino acids and nitrogen-containing heterocyclic compounds [18,19,20], we report herein the synthesis of a series of 1-(5-chloro-2-hydroxyphenyl)-5-oxopyrrolidine-3-carboxylic acid derivatives bearing heterocyclic moieties and evaluation of their antioxidant and anticancer activities.

## 2. Results and Discussion

### 2.1. Chemistry

2-(3-((4-Methoxyphenyl)amino)propanoyl)-*N*-phenylhydrazinecarboxamide (**2**) and its thio analogue were synthesized from 3-((4-methoxyphenyl)amino)propanehydrazide (**1**) by reaction with phenyl isocyanate and phenyl isothiocyanate, respectively (Scheme 1). Structures of all synthesized compounds have been confirmed by the ^1^H- and ^13^C-NMR spectra and HRMS data (Supplementary Material, Figures S1–S120). In the ^13^C-NMR spectrum for hydrazinecarboxamide **2**, carbon in C=O group of semicarbazide moiety resonated at 170.34 ppm, whereas analogous carbon (C=S) in thiosemicarbazide moiety gave a signal at 180.83 ppm in the ^13^C-NMR spectrum for **3**.

Reactions of **1** with isocyanate and isothiocyanate resulted in formation of diphenylcarbamoyl hydrazide **4** and its thio analogue **5**, respectively. In the ^13^C-NMR spectrum of **4**, carbon of C=O in the second phenyl carbamoyl moiety resonated at 155.33 ppm, whereas analogous carbon (C=S) in thiocarbamoyl group resonated at 181.67 ppm in the ^13^C-NMR spectrum for **5**. In the ^13^C-NMR spectrum for **5**, the resonance of carbonyl carbon shifted downfield (170.2 ppm) in comparison with the resonance of this group carbon in the spectrum of **3** (158.59 ppm). Condensation reaction of hydrazinecarbothioamide **5** in concentrated sulfuric acid provided thiadiazole derivative **6** in 77% yield. In the ^1^H-NMR spectrum for **6**, two singlets of NH group protons are observed at 8.66 ppm and 10.29 ppm in comparison with the ^1^H-NMR spectrum of **5**, in which four singlets of NH groups are present. 

Condensation reactions of phenylhydrazinecarboxamide **2** and its thio analogue **3** in alkaline medium resulted in formation of triazolone **7** and triazolethione **8** derivatives, respectively, in 80% yield. In the ^13^C-NMR spectrum for **7**, the resonance of carbonyl group carbon in triazolone ring (154.44 ppm) shifted upfield in comparison with the spectrum of open-chain precursor **2**. The same pattern in the resonance of the C=S carbon (172.08 ppm) in triazolethione moiety of **8** has been observed in respect with the resonance of the corresponding carbon in an open-chain derivative **3**. Alkylation reactions of triazolone **7** with 2-bromoacetophenone and 2-bromo-4′-fluoroacetophenone afforded derivatives **9** and **10**, respectively. In their ^1^H-NMR spectra, the singlets at 4.8 ppm have been attributed to the CH_2_ group in acetophenone moiety. 

Introduction of the acetyl group usually enhances biological activity of the compound. Therefore, triazolethione **8** was heated at reflux with acetyl chloride to afford *N*-(4-methoxyphenyl)-*N*-(2-(4-phenyl-5-thioxo-4,5-dihydro-1*H*-1,2,4-triazol-3-yl)ethyl)acetamide (**11**). The introduction of acetyl moiety has been confirmed by the carbonyl carbon resonance at 167.71 ppm in its ^13^C-NMR spectrum. Treatment of triazolone **7** and triazolethione **8** with potassium thiocyanate in acetic acid gave corresponding thioureas **12a** and **12b**. In their ^13^C-NMR spectra, the carbon resonances at 181.93 ppm have been attributed to the carbons in thiourea moiety. Thiazole moiety was introduced into the structure of **12b** by its reaction with maleic anhydride in 1,4-dioxane at reflux temperature of the reaction mixture to afford compound **13**. Formation of the thiazole ring in **13** has been confirmed by the ^13^C-NMR carbon resonances at 51.21 ppm (CS), 182.95 ppm (NCS), and 187.44 ppm (C=O). 

**Scheme 2:**
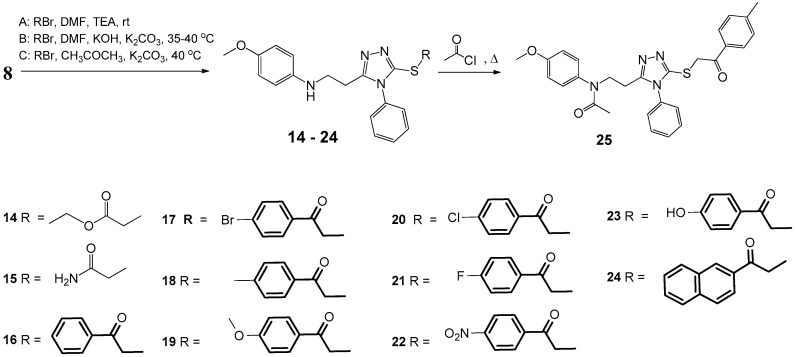
Synthesis of compounds **14**–**25**.

Thione-thiol tautomerism is characteristic of triazolethiones, therefore they easily form *S*-substituted derivatives, which possess a broad spectrum of biological activities. A series of *S*-substituted triazolethione derivatives **14**–**24** were prepared by alkylation reaction of triazolethione **8** with corresponding aliphatic and aromatic halocarbonyl compounds using three methods (A, B, and C) (Scheme 2) [18].

Method A was used to carry out alkylation with ethyl chloroacetate and several acetophenones in DMF in the presence of trimethylamine to afford derivatives **14**, **16**, **17**, and **20**. Alkylation of **8** with 2-chloroacetamide in the presence of KOH and K_2_CO_3_ was employed for the synthesis of **15** (Method B). Compounds **18**, **19**, and **21**–**24** were synthesized according to the Method C in acetone in the presence of K_2_CO_3_. The IR spectra of *S*-substituted triazole derivatives display absorption bands of carbonyl group in the region of 1649–1753 cm^−1^, whereas the absorption band of C=S group, which is present at 1237 cm^−1^ in the IR spectrum for **8**, is absent. In the ^13^C-NMR spectra for **14** and **15**, C-S- group carbons resonated at approx. 168 ppm, whereas carbons of the same group with aromatic moiety in the attached substituent resonated in the range of 191–193 ppm in the ^13^C-NMR spectra for compounds **16**–**24**. Acetyl group was introduced into the structure of **18** in its reaction with acetyl chloride at reflux temperature to afford acetamide **25**. The singlet of CH_3_ group protons at 1.61 ppm in the ^1^H-NMR spectrum for **25** has been ascribed to the methyl group in acetyl moiety. 

**Scheme 3:**
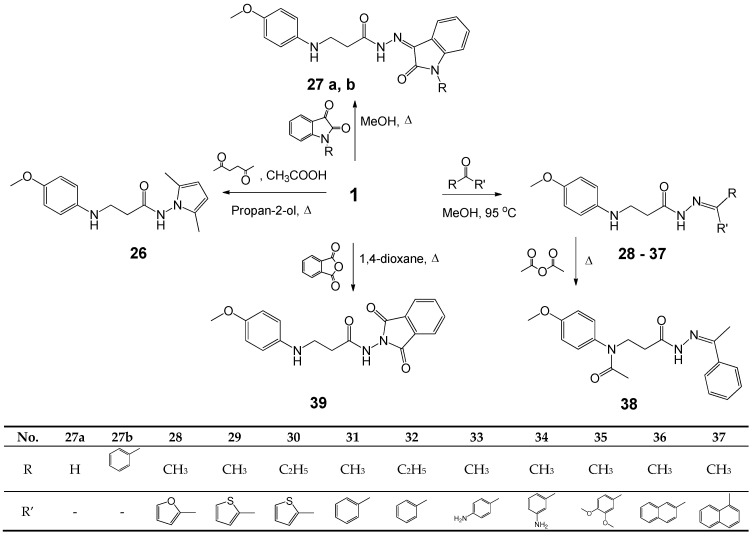
Synthesis of compounds **26**–**39**.

Condensation reaction of **1** with hexane-2,5-dione in propan-2-ol in the presence of acetic acid as a catalyst afforded *N*-(2,5-dimethyl-1*H*-pyrrol-1-yl)-3-((4-methoxyphenyl)amino)propanamide (**26**) (Scheme 3). The formation of pyrrole ring has been confirmed by the singlets of two methyl group protons at 1.98 ppm and 2.02 ppm and singlets of the CH group protons at 5.63 ppm and 5.71 ppm in the ^1^H-NMR spectrum for **26**. Reaction of **1** with isatin or *N*-benzylisatin in methanol in the presence of glacial acetic acid as a catalyst provided compounds **27a** and **27b** in 72% and 78% yield, respectively. The singlet at 5.26 ppm has been ascribed to the NH group proton in the 2-oxindole moiety in the ^1^H-NMR spectrum for **27a**. In the ^1^H-NMR spectrum for **27b**, the protons of the methylene group in benzyl moiety resonated as a singlet at 5.01 ppm.

By employing the most often used method for the synthesis of hydrazone-type compounds, i.e., the reaction of hydrazides with carbonyl compounds, Schiff bases **28**–**37** were synthesized by condensation reaction of **1** and corresponding disubstituted ketones in methanol at reflux temperature of the reaction mixtures [19,21]. The ^1^H-NMR spectra for these compounds display double sets of resonances of the CO–NH group protons with signal intensity ratio 0.6 : 0.4 due to the restricted rotation around the amide bond. This splitting of the proton resonances indicates that in DMSO-*d_6_* solution hydrazones exist as a mixture of *Z/E* isomers and, usually, the *Z* isomer predominates [22,23]. 

Acetamide **38** was synthesized by treating **30** with acetic anhydride in methanol at reflux temperature of the reaction mixture. The singlet of CH_3_ group protons at 1.70 ppm in the ^1^H-NMR spectrum for **38** has been ascribed to the methyl group in acetyl moiety. Reaction of **1** with phthalic anhydride in 1,4-dioxane at reflux temperature resulted in formation of propanamide **39** in 81% yield. The proton resonances of the benzene ring of the phthalimide moiety are observed in the range of 7.37–7.70 ppm. In the ^13^C-NMR spectrum for **39**, the carbon resonance of double intensity at 173.09 ppm has been attributed to the carbonyl group carbons in phthalimide moiety.

### 2.2. Evaluation of Antioxidant Activity

Antioxidant properties of compounds **1**–**39** were evaluated by 2,2-diphenyl-1-(2,4,6-trinitrophenyl)hydrazyl (DPPH) radical scavenging method [4]. The DPPH radical is a stable free radical that is commonly used as a substrate to evaluate *in vitro* antioxidant activity [24]. Compound possessing antioxidant property donates a hydrogen atom or electrons to DPPH and converts it to a stable molecule, 1,1-diphenyl-picryl hydrazine. DPPH assay is considered to be an accurate, easy and economic method to evaluate radical scavenging activity of antioxidants, since the radical compound is stable and needs not to be generated [25].

Antioxidant activity of *N*-(1,3-dioxoisoindolin-2-yl)-3-((4-methoxyphenyl)amino)propanamide (**39**) has been tested to be 1.37 times higher than that of a well-known antioxidant ascorbic acid, whereas antioxidant activity of 3-((4-methoxyphenyl)amino)-*N’*-(1-(naphthalen-1-yl)ethylidene)-propanehydrazide (**36**) surpassed that of ascorbic acid by 1.35-fold (Table 1). It is interesting to note that hydrazone bearing 2-naphthalene moiety **37** scavenged DPPH radical weaker than hydrazine **36**, but still at the level of vitamin C. Antioxidant activity of hydrazone bearing thiophene moiety **29** was 1.26 times higher than that of the positive control.

Hydrazone **30**, which structure differs from the one of **29** just by ethyl group instead of methyl one, exhibited much lower DPPH scavenging activity (55.92%). Introduction of the furan moiety into hydrazone molecule **28** led to an even lower antioxidant activity. The presence of aniline moiety in these molecules led to inactive compounds **33** and **34**. Acylation of compound **31** (33.18%) provided almost inactive acetamide **38** (7.89%).

Compounds **3**, **14**, and **27a** have been identified as possessing quite high antioxidant activity as well. The antioxidant activity according to DPPH inhibition decreases in the following order among the investigated compounds: **39** > **36 > 29** > **3** > **14** > **27a** > **17** ≈ **35** > **6** > **20** ≈ **15** > **37** ≈ **16** > ascorbic acid > **30** > **1** > **28**

1-(4-Bromophenyl)-2-((5-(2-((4-methoxyphenyl)amino)ethyl)-4-phenyl-4*H*-1,2,4-triazol-3-yl)-thio)ethanone (**17**) has been shown to possess the highest antioxidant activity among the series of *S*-substituted triazolethione derivatives **14**–**24**. Its DPPH radical scavenging activity has been determined to be 1.13 times higher than that of ascorbic acid. Replacement of bromine substituent in **17** with chlorine one in **20** led to a slight decrease in activity, whereas activity of **21** bearing fluorine atom decreased almost twice. Introduction of hydroxyl group led to almost complete inactivity of derivative **23**. The same loss of moderate activity of **18** was caused by introduction of acetyl group into the structure of acetamide **25**. A similar pattern in the decrease of antioxidant activity has been observed in a pair of triazolethione derivatives **8** (45.1%) and **11** (20.2%), proving the importance of the presence of N-H functional group, which can donate a hydrogen atom. Replacement of this hydrogen atom in triazolone **7** (29.98%) with much bulkier functional groups resulted in completely inactive derivatives **9** and **10**.

### 2.3. Evaluation of Anticancer Activity

**Figure 1:**
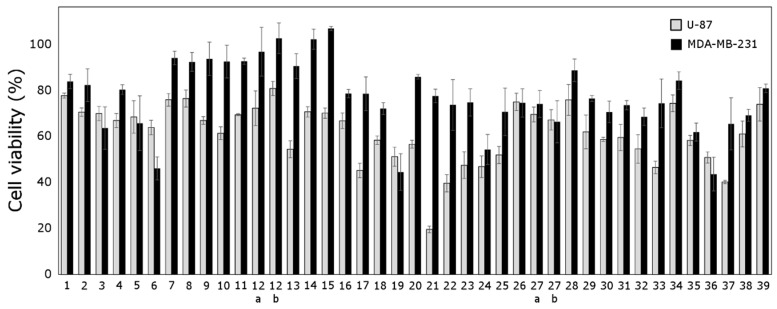
Cell viability reducing activity of the synthesized compounds **1**–**39**.

The synthesized compounds **1**–**39** have been tested to possess different activity against human glioblastoma U-87 and triple-negative breast cancer MDA-MB-231 cell lines at 100 µM concentration. In general, compounds showed relatively low activity against the cancer cell lines used in the screening experiments (Figure 1). This could be explained by the presence of drug efflux systems in brain tumours contributing to the drug resistance [26]. Triple-negative breast cancer is typically more resistant to majority of available drugs due to the higher expression of P-glycoprotein that enhances drug efflux from the nucleus [27,28], also epigenetic alterations in histone deacetylase [29], and other mechanisms. Cell line U-87 is quite resistant to temozolomide, which is clinically used drug to treat glioblastoma. It reduces U-87 cell viability up to 60% at 100 µM concentration after 48 h of incubation [30]. It is worthy to note that activity depends a lot on the mechanism of action. For example, topoisomerase inhibitor etoposide at 100 µM concentration reduces MDA-MB-231 cell viability up to approximately 80% after 72 h incubation [31], antimicrotubular drug docetaxel at 100 nM concentration reduces cell viability up to 40% already after 48 h of incubation [32], and novel kinase inhibitor dasatinib at 1 µM concentration reduces the viability up to 30% [33].

A majority of the synthesized compounds were more active against glioblastoma U-87 than the triple-negative breast cancer cell line MDA-MB-231. Cisplatin (a clinically used alkylating agent) also shows similar higher activity towards glioblastoma cells. It almost completely inhibited U-87 cell proliferation after 48 h incubation [30,34], and reduced MDA-MB-231 cell viability only up to 30% at 10 µM concentration after 72 h of incubation [31].

1-(4-Fluorophenyl)-2-((5-(2-((4-methoxyphenyl)amino)ethyl)-4-phenyl-4*H*-1,2,4-triazol-3-yl)thio)ethanone (**21**), which reduced cell viability up to 19.6 ± 1.5%, has been identified as the most active compound against glioblastoma cells. However, its’ analogues bearing bromine, methyl, methoxy, chlorine and hydroxyl substituents (**17**–**20** and **23**) showed at least twice lower activity. Thiophenyltriazole **16** with no substituent at *para*-position of benzene ring was even less active in comparison with the *para*-substituted ones. It can be assumed, that only small substituents (such as fluorine) could be introduced at this position. Possibly, this fluorination prevents metabolism due to which the anticancer activity could be reduced [35]. 

Thiophenyltriazole **22** and hydrazone **37** were also among the most active compounds against U-87 cell line; they reduced cell viability up to 39.8 ± 3.8% and 40.3 ± 0.8%, respectively. It is worthy to note, that **22**, instead of fluorine at the *para*-position in **21**, contains nitro group, which is often associated with anticancer properties as well as mutagenic and genotoxic ones [36]. Hydrazone **37** has been identified as a highly active antioxidant substance, proving that these two properties could be related to each other.

Compounds **36**, **19** and **6** have shown the highest activity against MDA-MB-231 cell line. They reduced breast cancer cell viability up to 43.7 ± 7.4%, 44.6 ± 8.0%, and 46.2 ± 5.0%, respectively. Both hydrazones **36** and **37** contain bulky naphthalene moiety in their structure differing only in the position of substitution. Both of them have shown high antioxidant properties. This cross-activity could be non-specific and related to their radical scavenging activity.

Thiophene derivatives **29** and **30**, which have been identified as strong antioxidants in DPPH assay, exhibited a very weak effect on cell viability. It could be explained by our previous findings that antioxidant and anticancer activity relationship could not be always explained [37], as many other different mechanisms of action contribute to the anticancer activity of different structures.

In summary, fluorine-substitututed thiophenyltriazole **21**, which has shown different activity on different cell lines, possibly due to selectivity on specific targets in glioblastoma cells, has been identified as possessing the promising anticancer activity.

## 3. Experimental

### 3.1. General Information

Reagents were purchased from Sigma-Aldrich (St. Louis, MO, USA) and TCI Europe N.V. (Zwijndrecht, Belgium). Reaction course and purity of the synthesized compounds was monitored by TLC using aluminium plates precoated with silica gel 60 F254 (Merck KGaA, Darmstadt, Germany). Melting points were determined on a MEL-TEMP (Electrothermal, A Bibby Scientific Company, Burlington, NJ, USA) melting point apparatus and are uncorrected. FT-IR spectra (ν, cm^−1^) were recorded on a Spectrum BX FT–IR spectrometer (Perkin–Elmer Inc., Waltham, MA, USA) using KBr pellets. The ^1^H- and ^13^C-NMR spectra were recorded in DMSO-*d_6_* on a Varian Unity Inova (300 MHz, 75 MHz, Palo Alto, CA, USA) and Bruker Avance III (400 MHz, 100 MHz Bruker BioSpin AG, Fällanden, Switzerland ) spectrometers operating in the Fourier transform mode. Chemical shifts (*δ*) are reported in parts per million (ppm) calibrated from TMS (0 ppm) as an internal standard for ^1^H-NMR, and DMSO-*d_6_* (39.43 ppm) for ^13^C-NMR. Mass spectra were obtained on a maXis UHR-TOF mass spectrometer (Bruker Daltonics, Bremen, Germany) with ESI ionization.

### 3.2. Synthesis

*2-(3-((4-Methoxyphenyl)amino)propanoyl)-N-phenylhydrazinecarboxamide* (**2**): A mixture of propane-hydrazide (**1**, 2.09 g, 10 mmol), methanol (30 mL), and phenyl isocyanate (1.35 g, 1.08 mL, 10 mmol) was heated at reflux for 30 min. The reaction mixture was cooled down, precipitate formed was filtered off and recrystallized from DMF/H_2_O mixture. Yield 59%; white solid; m.p. 192–193 °C; IR (KBr) ν_max_ (cm^−1^): 3375, 3344, 3283, 3206 (NH), 1659, 1638 (C=O); ^1^H-NMR (DMSO-*d_6_*, 400 MHz) δ: 2.41 (t, *J* = 7.2 Hz, 2H, CH_2_CO), 3.78 (s, 3H, CH_3_O), 3.84 (t, *J* = 7.2 Hz, 2H, *CH_2_*NH), 6.99–7.44 (m, 9H, H_Ar_), 7.76, 8.01, 8.71, 9.77 (4s, 4H, 4NH); ^13^C-NMR (DMSO-*d_6_*, 101 MHz) δ: 32.3 (*CH_2_*CO), 46.4 (CH_2_NH), 55.3 (CH_3_O), 114.9, 119.9, 128.3, 128.7, 129.7, 134.1, 139.6, 140.0, 154.7 (C_Ar_), 158.1, 170.3 (C=O); HRMS (ESI): *m/z* calcd for C_17_H_20_N_4_O_3_ 328.1535 [M + H]^+^, found 328.1537.

*2-(3-((4-Methoxyphenyl)amino)propanoyl)-N-phenylhydrazinecarbothioamide* (**3**): A mixture of propanehydrazide **1** (3.15 g, 15 mmol), methanol (60 mL), and phenyl isothiocyanate (0.42 g, 3.71 mL, 20 mmol) was heated at reflux for 4 h. The reaction mixture was cooled down, precipitate formed was filtered off and recrystallized from DMF/H_2_O mixture. Yield 78%; white solid; m.p. 164–165 °C; IR (KBr) ν_max_ (cm^−1^): 3451–2831 (NH), 1698 (C=O), 1244 (C=S); ^1^H-NMR (DMSO-*d_6_*, 400 MHz) δ: 2.46 (t, *J* = 7.2 Hz, 2H, CH_2_CO), 3.24 (t, *J* = 7.2 Hz, 2H, *CH_2_*NH); 3.63 (s, 3H, CH_3_O), 5.18 (s, 1H, NH), 6.55 (d, *J* = 8.8 Hz, 2H, H_Ar2,6_), 6.73 (d, *J* = 8.8 Hz, 2H, H_Ar3,5_), 7.21–7.37 (m, 5H, H_Ar‘_), 9.54, 9.59 (2s, 2H, *NHNH*CS), 9.98 (s, 1H, NHAr‘); ^13^C-NMR (DMSO-*d_6_*, 101 MHz) δ: 33.5 (*CH_2_*CO), 40.2 (CH_2_NH), 55.3 (CH_3_O), 113.4, 114.7, 115.29, 126.1, 127.9, 139.1, 142.9, 150.9 (C_Ar_), 158.6 (C=O), 180.8 (C=S); HRMS (ESI): *m*/*z* calcd for C_17_H_20_N_4_O_2_S 345.1385 [M + H]^+^, found 345.1379.

#### 3.2.1. General Procedure for Synthesis of Compounds **4** and **5**

To propanehydrazide **1** (2.09 g, 0.01 mol) dissolved in methanol (70 mL), corresponding cyanate (0.02 mol) was added. The reaction mixture was heated at reflux for 1–2 h. Precipitate formed was filtered off, washed with water, and recrystallized from DMF/H_2_O mixture.

*2-(3-(1-(4-Methoxyphenyl)-3-phenylureido)propanoyl)-N-phenylhydrazinecarboxamide* (**4**): Prepared from phenyl isocyanate by heating at reflux a reaction mixture for 2 h. Yield 76%; white solid; m.p. 179–180 °C; IR (KBr) ν_max_ (cm^−1^): 3386, 3254, 3283, 3206 (NH); 1675, 1661, 1601 (C=O); ^1^H-NMR (DMSO-*d_6_*, 400 MHz) δ: 2.43 (t, *J* = 7.2 Hz, 2H, CH_2_CO), 3.79 (s, 3H, CH_3_O), 3.85 (t, *J* = 7.2 Hz, 2H, CH_2_N), 6.88–7.48 (m, 14H, H_Ar, Ar’, Ar’’_), 7.72 (s, 1H, NH), 8.00 (s, 1H, NH), 8.69 (s, 1H, NH), 9.76 (s, 1H, NH); ^13^C-NMR (DMSO-*d_6_*, 101 MHz) δ: 32.3 (*CH_2_*CO), 46.2 (CH_2_N), 55.3 (CH_3_O), 114.9, 118.6, 119.8, 121.9, 122.0, 128.2, 128.6, 129.4, 129.6, 134.1, 139.6, 139.9, 154.6 (C_Ar_), 155.3, 158.1, 170.5 (C=O); HRMS (ESI): *m*/*z* calcd for C_24_H_25_N_5_O_4_ 447.1985 [M + H]^+^, found 448.1987.

*2-(3-(1-(4-Methoxyphenyl)-3-phenylthioureido)propanoyl)-N-phenylhydrazinecarbothioamide* (**5**): Prepared from phenyl isothiocyanate by heating at reflux a reaction mixture for 1 h. Yield 78%; white solid; m.p. 178–179 °C; IR (KBr) ν_max_ (cm^−1^): 3333, 3299, 3206, 3154 (NH), 1698 (C=O), 1243, 1216 (C=S); ^1^H-NMR (DMSO-*d_6_*, 400 MHz) δ: 2.64 (t, *J* = 7.8 Hz, 2H, CH_2_CO), 3.77, 3.78 (2s, 3H, CH_3_O), 4.34 (t, *J* = 7.8 Hz, 2H, CH_2_N), 6.96–7.34 (m, 14H, H_Ar, Ar’, Ar’’_), 7.38, 8.65, 9.50, 9.95 (4s, 4H, 4NH); ^13^C-NMR (DMSO-*d_6_*, 101 MHz) δ: 31.0 (*CH_2_*CO), 50.6 (CH_2_N), 55.4 (CH_3_O), 115.1, 115.2, 124.7, 125.8, 126.0, 127.8, 128.0, 129.1, 134.7, 139.1, 140.5, 158.6 (C_Ar_), 170.20 (C=O), 180.8, 181.7 (C=S); HRMS (ESI): *m*/*z* calcd for C_24_H_25_N_5_O_2_S_2_ 480.1528 [M + H]^+^, found 480.1526.

*1-(4-Methoxyphenyl)-3-phenyl-1-(2-(5-(phenylamino)-1,3,4-thiadiazol-2-yl)ethyl)thiourea* (**6**): A mixture of conc. H_2_SO_4_ (25 mL) and phenylhydrazinecarbothioamide **5** (0.96 g, 2 mmol) was stirred at room temperature until all solid dissolved (approx. 25 min). Afterwards the reaction mixture was added drop by drop to a water-ice mixture. Precipitate formed was filtered off, washed with water, and recrystallized from propan-2-ol. Yield 77%; white solid; m.p. 151–152 °C; IR (KBr) ν_max_ (cm^−1^): 3358, 3191 (NH), 1249 (C=S); ^1^H-NMR (DMSO-*d_6_*, 400 MHz) δ: 3.29 (t, *J* = 7.4 Hz, 2H, CH_2_C), 3.76 (s, 3H, CH_3_O), 4.46 (t, *J* = 7.4 Hz, 2H, CH_2_N), 6.96–7.59 (m, 14H, H_Ar, Ar’, Ar’’_), 8.66 (s, 1H, NH), 10.29 (s, 1H, NHC=S); ^13^C-NMR (DMSO-*d_6_*, 101 MHz) δ: 27.8 (*CH_2_*C), 53.8 (CH_2_N), 55.3, 55.5 (CH_3_O), 115.0, 115.3, 117.2, 121.7, 124.9, 126.3, 127.8, 129.1, 134.6, 140.5, 140.8, 156.4, 158.6, 164.4 (C_Ar_), 181.9 (C=S); HRMS (ESI): *m*/*z* calcd for C_24_H_23_N_5_OS_2_ 462.1422 [M + H]^+^, found 462.1419.

*3-(2-((4-Methoxyphenyl)amino)ethyl)-4-phenyl-1H-1,2,4-triazol-5(4H)-one* (**7**): A mixture of phenyl-hydrazinecarboxamide **2** (1.34 g, 3 mmol) and 20% aqueous KOH solution (25 mL) was heated at reflux for 2 h. The reaction mixture was cooled down and acidified with HCl to p*H* 4. Precipitate formed was filtered off, washed with water, and recrystallized from DMF/H_2_O mixture. Yield 81%; white solid; m.p. 156–157 °C; IR (KBr) ν_max_ (cm^−1^): 3175, 3017 (NH), 1659 (C=O); ^1^H-NMR (DMSO-*d_6_*, 400 MHz) δ: 2.62 (t, *J* =7.2 Hz, 2H, CH_2_C), 3.14 (t, *J* =7.2 Hz, 2H, *CH_2_*NH), 3.62 (s, 3H, CH_3_O); 6.39 (d, *J* = 8.4 Hz, 2H, H_Ar2,6_), 6.67 (d, *J* = 8.4 Hz, 2H, H_Ar3,5_), 7.41–7.57 (m, 6H, H_Ar’_+NH), 11.76 (s, 1H, NH); ^13^C-NMR (DMSO-*d_6_*, 101 MHz) δ: 25.6 (*CH_2_*C), 40.8 (CH_2_NH), 55.3 (CH_3_O), 113.2, 114.7, 127.6, 128.8, 129.6, 133.0, 145.2, 145.3, 150.9, 151.5 (C_Ar_), 154.4 (C=O); HRMS (ESI): *m*/*z* calcd for C_17_H_18_N_4_O_2_ 311.1509 [M + H]^+^, found 311.1503.

*3-(2-((4-Methoxyphenyl)amino)ethyl)-4-phenyl-1H-1,2,4-triazole-5(4H)-thione* (**8**): A mixture of phenyl-hydrazinecarbothioamide **3** (1 g, 3 mmol) and 20% aqueous KOH solution (40 mL) was heated at reflux for 4 h. The reaction mixture was cooled down and acidified with HCl to p*H* 4. Precipitate formed was filtered off, washed with water, and recrystallized from DMF/H_2_O mixture. Yield 80%; white solid; m.p. 141–142 °C; IR (KBr) ν_max_ (cm^−1^): 3446–2826 (NH), 1237 (C=S); ^1^H-NMR (DMSO-*d_6_*, 400 MHz) δ: 2.63 (t, *J* = 9.0 Hz, 2H, CH_2_C), 3.15 (t, 2H, *J* = 9.0 Hz, *CH_2_*NH), 3.61 (s, 3H, CH_3_O), 5.22 (s, 1H, NHAr’), 6.31 (d, *J* = 9 Hz, 2H, H_Ar2,6_), 6.63 (d, *J* = 9 Hz, 2H, H_Ar3,5_), 7.39–7.58 (m, 5H, H_Ar’_), 13.75 (s, 1H, NH); ^13^C-NMR (DMSO-*d_6_*, 101 MHz) δ: 25.4 (*CH_2_*C), 40.4 (CH_2_NH), 55.3 (CH_3_O), 113.1, 114.7, 122.0, 128.5, 129.5, 133.8, 142.2, 150.7, 150.9, 167.6 (C_Ar_), 172.1 (C=S); HRMS (ESI): *m*/*z* calcd for C_17_H_18_N_4_OS 327.1279 [M + H]^+^, found 327.1293.

#### 3.2.2. General Procedure for the Synthesis of 1,2,4-Triazolones **9** and **10**

To hydrazinecarboxamide **7** (0.93 g, 3 mmol) dissolved in DMF (15 mL), KOH (0.17 g, 3 mmol), K_2_CO_3_ (0.37 g, 2.7 mmol), and corresponding bromoacetophenone (3.75 mmol) were added. The reaction mixture was stirred at 40 °C for 24 h. Afterwards water (30 mL) was added to the reaction mixture. Precipitate formed was filtered off, washed with water, and recrystallized from propan-2-ol.

*3-(2-((4-Methoxyphenyl)(2-oxo-2-phenylethyl)amino)ethyl)-4-phenyl-1H-1,2,4-triazol-5(4H)-one* (**9**): Prepared from 2-bromoacetophenone. Yield 63%; white solid; m.p. 142–143 °C; IR (KBr) ν_max_ (cm^−1^): 3375 (NH), 1694, 1513 (C=O); ^1^H-NMR (DMSO-*d_6_*, 400 MHz) δ: 2.70 (t, *J* = 7.4 Hz, 2H, CH_2_C), 3.49 (t, *J* = 7.4 Hz, 2H, CH_2_N), 3.61 (s, 3H, CH_3_O), 4.80 (s, 2H, CH_2_CO), 6.22 (d, *J* = 9.2 Hz, 2H, H_Ar2,6_), 6.62 (d, *J* = 9.2 Hz, 2H, H_Ar3,5_), 7.41–7.96 (m, 10H, H_Ar’, Ar’’_), 11.68, 11.71 (2s, 1H, NH); ^13^C-NMR (DMSO-*d_6_*, 101 MHz) δ: 24.1 (*CH_2_*C), 40.2 (CH_2_N), 48.4 (*CH_2_*CO), 55.3 (CH_3_O), 112.6, 114.6, 127.5, 127.6, 127.7, 128.7, 128.8, 129.5, 132.9, 133.4, 135.3, 141.6, 145.4, 150.7 (C_Ar_), 154.3, 197.2 (C=O); HRMS (ESI): *m*/*z* calcd for C_25_H_24_N_4_O_3_ 429.1927 [M + H]^+^, found 429.1930.

*3-(2-((2-(4-Fluorophenyl)-2-oxoethyl)(4-methoxyphenyl)amino)ethyl)-4-phenyl-1H-1,2,4-triazol-5(4H)-one* (**10**): Prepared from 2-bromo-4′-fluoroacetophenone. Yield 58%; white solid; m.p. 103–104 °C; IR (KBr) ν_max_ (cm^−1^): 3494 (NH), 1698, 1514 (C=O); ^1^H-NMR (DMSO-*d_6_*, 400 MHz) δ: 2.70 (t, *J* = 7.4 Hz, 2H, CH_2_C), 3.48 (t, *J* = 7.4 Hz, 2H, CH_2_N), 3.61 (s, 3H, CH_3_O), 4.80 (s, 2H, CH_2_CO), 6.23 (d, *J* = 9.2 Hz, 2H, H_Ar2,6_), 6.62 (d, *J* = 9.2 Hz, 2H, H_Ar3,5_), 7.35–7.55 (m, 7H, H_Ar’, Ar’’_), 8.02–8.05 (m, 2H, H_Ar’’_), 11.67, 11.71 (2s, 1H, NH); ^13^C-NMR (DMSO-*d_6_*, 101 MHz) δ: 24.1 (*CH_2_*C), 40.2 (CH_2_N), 48.3 (*CH_2_*CO), 55.3 (CH_3_O), 112.7, 114.6, 115.6, 115.9, 127.6, 128.7, 129.5, 130.7, 130.8, 132.0, 132.0, 132.9, 141.6, 145.3, 150.7, 154.3, 163.9 (C_Ar_), 166.4, 195.8 (C=O); HRMS (ESI): *m*/*z* calcd for C_25_H_23_FN_4_O_3_ 447.1833 [M + H]^+^, found 447.1830.

*N-(4-Methoxyphenyl)-N-(2-(4-phenyl-5-thioxo-4,5-dihydro-1H-1,2,4-triazol-3-yl)ethyl)acetamide* (**11**): A mixture of triazolethione **8** (2.61 g, 8 mmol) and acetyl chloride (25 mL) was heated at reflux for 7 h. The reaction mixture was added drop by drop onto ice. Precipitate formed was filtered off, washed with water, and recrystallized from acetone. Yield 66%; white solid; m.p. 182–183 °C; IR (KBr) ν_max_ (cm^−1^): 1651, 1510 (C=O), 1255 (C=S); ^1^H-NMR (DMSO-*d_6_*, 400 MHz) δ: 1.62, 1.65 (2s, 3H, CH3), 2.60 (t, *J* = 6.8, 2H, CH_2_C), 3.56–3.70 (m, 2H, CH_2_N), 3.77 (s, 3H, CH_3_O), 6.94–7.56 (m, 9H, H_Ar_); 13.70 (s, 1H, NH); ^13^C-NMR (DMSO-*d_6_*, 101 MHz) δ: 22.2, 23.8 (CH_3_), 24.7 (*CH_2_*C), 45.4 (CH_2_N), 55.4 (CH_3_O), 114.7, 128.3, 129.2, 129.3, 129.5, 133.6, 135.0, 149.8, 158.4, 158.5 (C_Ar_), 167.7 (C=O), 169.4 (C=S); HRMS (ESI): *m*/*z* calcd for C_19_H_20_N_4_O_2_S 369.1386 [M + H]^+^, found 369.1381. 

#### 3.2.3. General Procedure for the Synthesis of Compounds **12a**, **b**

A mixture of triazolethione **7** or **8** (2 mmol), potassium thiocyanate (0.39 g, 4 mmol), and acetic acid (10 mL) was heated at reflux for 5 min. The reaction mixture was cooled to room temperature and diluted with water (50 mL). Precipitate formed was filtered off, washed with water, and recrystallized from propan-2-ol. 

*1-(4-Methoxyphenyl)-1-(2-(5-oxo-4-phenyl-4,5-dihydro-1H-1,2,4-triazol-3-yl)ethyl)thiourea* (**12a**): Prepared from **7** (0.62 g). Yield 76%; white solid; m.p. 203–204 °C; IR (KBr) ν_max_ (cm^−1^): 3242, 3176 (NH, NH_2_), 1691 (C=O); ^1^H-NMR (DMSO-*d_6_*, 400 MHz) δ: 2.73 (t, 2H, *J* = 7.2 Hz, CH_2_C), 3.34, 3.61 (2s, 2H, NH_2_), 3.77 (s, 3H, CH_3_O), 4.07 (t, *J* = 7.2 Hz, 2H, CH_2_N), 6.95 (d, *J* = 8.8 Hz, 2H, H_Ar2,6_), 7.02 (d, *J* = 8.8 Hz, 2H, H_Ar3,5_), 7.23–7.46 (m, 5H, H_Ar’_), 11.67, 11.71 (2s, 1H, NH); ^13^C-NMR (DMSO-*d_6_*, 101 MHz) δ: 24.2 (*CH_2_*C), 51.1 (CH_2_N), 55.4 (CH_3_O), 115.2, 127.2, 128.5, 128.8, 129.3, 132.7, 133.9, 144.4, 154.2 (C_Ar_), 158.6 (C=O), 181.9 (C=S); HRMS (ESI): *m*/*z* calcd for C_18_H_19_N_5_O_2_S 370.1337 [M + H]^+^, found 370.1337.

*1-(4-Methoxyphenyl)-1-(2-(4-phenyl-5-thioxo-4,5-dihydro-1H-1,2,4-triazol-3-yl)ethyl)thiourea* (**12b**): Prepared from **8** (0.65 g). Yield 66%; white solid; m.p. 198–199 °C; IR (KBr) ν_max_ (cm^−1^): 3233, 3185 (NH, NH_2_), 1520 (C=S); ^1^H-NMR (DMSO-*d_6_*, 400 MHz) δ: 2.73 (t, *J* = 7.2 Hz, 2H, CH_2_C), 3.34 (s, 2H, NH_2_), 3.77 (s, 3H, CH_3_O), 4.09 (t, *J* = 7.2 Hz, 2H, CH_2_N), 6.95 (d, *J* = 8.8 Hz, 2H, H_Ar2,6_), 7.02 (d, 2H, *J* = 8.8 Hz, H_Ar3,5_), 7.30–7.50 (m, 5H, H_Ar‘_), 13.70 (s, 1H, NH); ^13^C-NMR (DMSO-*d_6_*, 101 MHz) δ: 23.7 (*CH_2_*C), 51.2 (CH_2_N), 55.4 (CH_3_O), 114.7, 115.2, 128.1, 128.8, 129.3, 129.4, 133.5, 133.7, 149.6, 158.6 (C_Ar_), 167.6, 181.9 (C=S); HRMS (ESI): *m*/*z* calcd for C_18_H_19_N_5_OS_2_ 386.1109 [M + H]^+^, found 386.1104.

*2-(2-((4-Methoxyphenyl)(2-(4-phenyl-5-thioxo-4,5-dihydro-1H-1,2,4-triazol-3-yl)ethyl)amino)-4-oxo-4,5-dihydrothiazol-5-yl)acetic acid* (**13**): A mixture of **12b** (0.58 g, 1.5 mmol), maleic anhydride (0.29 g, 3 mmol), 1,4-dioxane (15 mL), and DMF (5 mL) was heated at reflux for 7 h. The reaction mixture was cooled down and cold water (10 mL) was added. Precipitate formed was filtered off and recrystallized from DMF/H_2_O mixture. Yield 82%; yellow solid; m.p. 159–160 °C; IR (KBr) ν_max_ (cm^−1^): 3233, 3185 (NH, NH_2_), 1648, 1713 (C=O), 1530 (C=S); ^1^H-NMR (DMSO-d_6_, 400 MHz) δ: 2.52–2.60 (m, 1H, CH), 2.73–2.83 (m, 2H, CH_2_C), 2.98–3.05 (m, 1H, CH_2_), 3.81 (s, 3H, CH_3_O), 3.88–4.09 (m, 2H, CH_2_N), 4.23–4.35 (m, 1H, CH_2_), 6.98–7.57 (m, 9H, H_Ar, Ar‘_), 12.78 (br s, 1H, OH), 13.75 (s, 1H, NH); ^13^C-NMR (DMSO-d_6_, 101 MHz) δ: 23.6 (CH_2_C), 37.6 (CH_2_), 50.3 (CH_2_N), 52.2 (CS), 55.6 (CH_3_O), 114.9, 128.3, 129.3, 129.4, 129.5, 132.5, 133.4, 149.1, 159.7 (C_Ar_), 167.8 (C=S), 172.4 (COOH), 183.0 (NCS), 187.4 (C=O); HRMS (ESI): m/z calcd for C_22_H_21_N_5_O_4_S_2_ 484.1113 [M + H]^+^, found 484.1110.

#### 3.2.4. General Procedure for the Synthesis of *S*-Substituted 1,2,4-Triazolethiones **14**–**24**

*Method A*. To triazolethione **8** (0.49 g, 1.5 mmol) dissolved in DMF (5 mL), triethylamine (0.20 g, 0.28 mL, 2 mmol) and corresponding halocarbonyl compound (2 mmol) were added. The reaction mixture was stirred at room temperature for 4 h. Afterwards cold water (30 mL) was added, the precipitate formed was filtered off and recrystallized from propan-2-ol. 

*Method B*. To triazolethione **8** (0.49 g, 1.5 mmol) dissolved in DMF (5 mL), KOH powder (0.11 g, 2 mmol), K_2_CO_3_ (0.28 g, 2.2 mmol), and corresponding halocarbonyl compound (2 mmol) were added. The reaction mixture was stirred at 35–40 °C for 24 h. Afterwards cold water (30 mL) was added, the precipitate formed was filtered off and recrystallized from propan-2-ol. 

*Method C*. To triazolethione **8** (0.49 g, 1.5 mmol) dissolved in acetone (15 mL), K_2_CO_3_ (1 g, 7.2 mmol) and corresponding halocarbonyl compound (2 mmol) were added. The reaction mixture was stirred at 40 °C for 3 h. Afterwards water (20 mL) was added, the precipitate formed was isolated by filtration, and recrystallized from propan-2-ol.

*((5-(2-((4-Methoxyphenyl)amino)ethyl)-4-phenyl-4H-1,2,4-triazol-3-yl)thio)methyl propionate* (**14**): Prepared according to *method A* from ethyl chloroacetate (0.25 g, 0.21 mL). Yield 80%; white solid; m.p. 71–72 °C; IR (KBr) ν_max_ (cm^−1^): 3309 (NH), 1753 (C=O); ^1^H-NMR (DMSO-*d_6_*, 400 MHz) δ: 1.17 (t, *J* = 7.2 Hz, 3H, *CH_3_*CH_2_), 2.73 (t, *J* = 7.2 Hz, 2H, CH_2_C), 3.19 (t, 2H, *J* = 7.2 Hz, 2H, *CH_2_*NH), 3.61, 3.62 (2s, 3H, CH_3_O), 4.01 (s, 2H, SCH_2_), 4.09 (q, *J* = 7.2 Hz, 2H, CH_3_*CH_2_*), 5.24 (s, 1H, NH), 6.31 (d, *J* = 8.8 Hz, 2H, H_Ar2,6_), 6.63 (d, *J* = 8.8 Hz, 2H, H_Ar3,5_), 7.44–7.64 (m, 5H, H_Ar’_); ^13^C-NMR (DMSO-*d_6_*, 101 MHz) δ: 14.0 (*CH_3_*CH_2_), 24.7 (*CH_2_*C), 33.9 (SCH_2_), 41.3 (CH_2_NH); 55.3 (CH_3_O), 61.2 (CH_3_*CH_2_*), 112.6, 113.1, 114.6, 127.4, 130.0, 130.1, 132.8, 142.2, 149.1, 150.8 (C_Ar_), 154.2 (C=O), 168.1 (C-S-); HRMS (ESI): *m*/*z* calcd for C_21_H_24_N_4_O_3_S 413.1647 [M + H]^+^, found 413.1660. 

*2-((5-(2-((4-Methoxyphenyl)amino)ethyl)-4-phenyl-4H-1,2,4-triazol-3-yl)thio)acetamide* (**15**): Prepared according to *method B* from 2-chloroacetamide (0.19 g). Yield 75%; white solid; m.p. 77–78 °C; IR (KBr) ν_max_ (cm^−1^): 3346–2826 (NH, NH_2_), 1696 (C=O); ^1^H-NMR (DMSO-*d_6_*, 400 MHz) δ: 2.74 (t, *J* = 7.2 Hz, 2H, CH_2_C), 3.20 (q, 2H, *J* = 7.2 Hz, *CH_2_*NH), 3.62 (s, 3H, CH_3_O), 3.88 (s, 2H, SCH_2_), 5.23 (t, *J* = 6.2 Hz, 1H, NH), 6.32 (d, *J* = 8.8 Hz, 2H, H_Ar2,6_), 6.64 (d, *J* = 8,8 Hz, 2H, H_Ar3,5_), 7.23 (s, 1H, NH_2_), 7.46–7.61 (m, 5H, H_Ar’_), 7.67 (s, 1H, NH_2_); ^13^C-NMR (DMSO-*d_6_*, 101 MHz) δ: 24.7 (*CH_2_*C), 35.9 (SCH_2_), 41.3 (CH_2_NH), 55.3 (CH_3_O), 113.1, 114.6, 127.4, 129.9, 130.1, 132.9, 142.2, 149.9, 150.8 (C_Ar_), 154.0 (C=O), 168.7 (C-S-); HRMS (ESI): *m*/*z* calcd for C_19_H_21_N_5_O_2_S 384.1494 [M + H]^+^, found 384.1500. 

*2-((5-(2-((4-Methoxyphenyl)amino)ethyl)-4-phenyl-4H-1,2,4-triazol-3-yl)thio)-1-phenylethanone* (**16**): Prepared according to *method A* from 2-bromoacetophenone (0.4 g). Yield 69%; white solid; m.p. 159–160 °C; IR (KBr) ν_max_ (cm^−1^): 3292 (NH), 1687 (C=O); ^1^H-NMR (DMSO-*d_6_*, 400 MHz) δ: 2.74 (t, *J* = 7.2 Hz, 2H, CH_2_C), 3.15–3.25 (m, 2H, *CH_2_*NH), 3.61 (s, 3H, CH_3_O), 4.86 (s, 2H, SCH_2_), 5.23 (s, 1H, NH), 6.32 (d, *J* = 8.8 Hz, 2H, H_Ar2,6_), 6.64 (d, *J* = 8.8 Hz, 2H, H_Ar3,5_), 7.46–8.02 (m, 10H, H_Ar’, Ar”_); ^13^C-NMR (DMSO-*d_6_*, 101 MHz) δ: 24.6 (*CH_2_*C), 40.0 (SCH_2_), 41.2 (CH_2_NH), 55.2 (CH_3_O), 112.9, 114.5, 127.3, 128.3, 128.7, 129.9, 132.8, 133.6, 135.2, 142.1, 149.3, 150.7 (C_Ar_), 154.0 (C=O), 193.1 (C-S-); HRMS (ESI): *m*/*z* calcd for C_25_H_24_N_4_O_2_S 445.1698 [M + H]^+^, found 445.1708.

*1-(4-Bromophenyl)-2-((5-(2-((4-methoxyphenyl)amino)ethyl)-4-phenyl-4H-1,2,4-triazol-3-yl)thio)ethanone* (**17**): Prepared according to *method A* from 4-bromophenacyl bromide (0.56 g). Yield 63%; white solid; m.p. 168–169 °C; IR (KBr) ν_max_ (cm^−1^): 3291 (NH), 1694 (C=O); ^1^H-NMR (DMSO-*d_6_*, 400 MHz) δ: 2.73 (t, *J* = 7.2 Hz, 2H, CH_2_C), 3.19 (t, 2H, *J* = 7.2 Hz, *CH_2_*NH), 3.61 (s, 3H, CH_3_O), 4.82 (s, 2H, SCH_2_), 5.22 (s, 1H, NH), 6.31 (d, *J* = 8.8 Hz, 2H, H_Ar2,6_), 6.63 (d, *J* = 8.8 Hz, 2H, H_Ar3,5_), 7.45–7.94 (m, 9H, H_Ar’, Ar”_); ^13^C-NMR (DMSO-*d_6_*, 101 MHz) δ: 24.8 (*CH_2_*C), 40.2 (SCH_2_), 41.3 (CH_2_NH), 55.3 (CH_3_O), 113.1, 114.6, 127.4, 127.8, 130.0, 130.1, 130.4, 131.9, 132.9, 134.3, 142.2, 149.2, 150.8 (C_Ar_), 154.2 (C=O), 192.5 (C-S-); HRMS (ESI): *m*/*z* calcd for C_25_H_23_BrN_4_O_2_S 523.0803 [M + H]^+^, found 523.0799.

*2-((5-(2-((4-Methoxyphenyl)amino)ethyl)-4-phenyl-4H-1,2,4-triazol-3-yl)thio)-1-(p-tolyl)ethanone* (**18**): Prepared according to *method C* from 2-bromo-4′-methylacetophenone (0.43 g). Yield 71%; white solid; m.p. 136–137 °C; IR (KBr) ν_max_ (cm^−1^): 3282 (NH), 1689 (C=O); ^1^H-NMR (DMSO-*d_6_*, 400 MHz) δ: 2.39 (s, 3H, CH_3_), 2.74 (t, *J* = 7.4 Hz, 2H, CH_2_C), 3.19 (q, *J* = 6.8, 14.0 Hz, 2H, *CH_2_*NH), 3.61 (s, 3H, CH_3_O), 4.82 (s, 2H, SCH_2_), 5.22 (t, *J* = 6.0 Hz, 1H, NH), 6.31 (d, *J* = 8.8 Hz, 2H, H_Ar2,6_), 6.63 (d, *J* = 8.8 Hz, 2H, H_Ar3,5_), 7.35 (d, *J* = 8.0 Hz, 2H, H_Ar’’_), 7.45–7.50 (m, 2H, H_Ar’_), 7.58–7.63 (m, 3H, H_Ar’_), 7.90 (d, *J* = 8.0 Hz, 2H, H_Ar’’_); ^13^C-NMR (DMSO-*d_6_*, 101 MHz) δ: 21.2 (CH_3_), 24.7 (*CH_2_*C), 40.1 (SCH_2_), 41.3 (CH_2_NH), 55.3 (CH_3_O), 113.1, 114.6, 127.4, 128.5, 129.4, 130.0, 130.1, 132.8, 132.9, 142.2, 144.3, 149.4, 150.8 (C_Ar_), 154.1 (C=O), 192.7 (C-S-); HRMS (ESI): *m*/*z* calcd for C_26_H_26_N_4_O_2_S 459.1854 [M + H]^+^, found 459.1853.

*1-(4-Methoxyphenyl)-2-((5-(2-((4-methoxyphenyl)amino)ethyl)-4-phenyl-4H-1,2,4-triazol-3-yl)thio)ethanone* (**19**): Prepared according to *method C* from 2-bromo-4’-methoxyacetophenone (0.30 g). Yield 76%; white solid; m.p. 121–122 °C; IR (KBr) ν_max_ (cm^−1^): 3287 (NH), 1683 (C=O); ^1^H-NMR (DMSO-*d_6_*, 400 MHz) δ: 2.76 (t, *J* = 7.2 Hz, 2H, CH_2_C), 3.25 (t, *J* = 7.2 Hz, 2H, *CH_2_*NH), 3.61, 3.64 (2s, 3H, CH_3_O), 3.85, 3.86 (2s, 3H, CH_3_O), 4.79, 4.80 (2s, 2H, SCH_2_), 5.22 (s, 1H, NH), 6.45–7.99 (m, 14H, H_Ar, Ar‘, Ar‘_‘+NH); ^13^C-NMR (DMSO-*d_6_*, 101 MHz) δ: 24.3 (*CH_2_*C), 40.0 (SCH_2_), 42.2 (CH_2_NH), 55.3, 55.3 (CH_3_O), 55.6, 55.6 (CH_3_O), 112.5, 114.0, 114.7, 127.4, 128.1, 130.0, 130.1, 130.8, 132.9, 141.6, 149.6, 150.6, 153.8 (C_Ar_), 163.6 (C=O), 191.5 (C-S-); HRMS (ESI): *m*/*z* calcd for C_26_H_26_N_4_O_3_S 475.1804 [M + H]^+^, found 475.1802.

*1-(4-Chlorophenyl)-2-((5-(2-((4-methoxyphenyl)amino)ethyl)-4-phenyl-4H-1,2,4-triazol-3-yl)thio)ethanone* (**20**): Prepared according to *method A* from 2-bromo-4’-chloroacetophenone (0.47 g). Yield 61%; white solid; m.p. 152–153 °C; IR (KBr) ν_max_ (cm^−1^): 3291 (NH), 1694 (C=O); ^1^H-NMR (DMSO-*d_6_*, 400 MHz) δ: 2.73 (t, *J* = 7.2 Hz, 2H, CH_2_C), 3.19 (t, 2H, *J* = 7.2 Hz, *CH_2_*NH), 3.61 (s, 3H, CH_3_O), 4.83 (s, 2H, SCH_2_), 5.25 (s, 1H, NH), 6.31 (d, *J* = 8.8 Hz, 2H, H_Ar2,6_), 6.63 (d, *J* = 8.8 Hz, 2H, H_Ar3,5_), 7.44–8.06 (m, 9H, H_Ar′, Ar″_); ^13^C-NMR (DMSO-*d_6_*, 101 MHz) δ: 24.72 (*CH_2_*C), 40.22 (SCH_2_), 41.29 (CH_2_NH), 55.28 (CH_3_O), 113.10, 114.62, 127.37, 128.92, 129.80, 130.00, 130.07, 130.32, 132.90, 134.01, 138.62, 142.15, 149.24, 150.82 (C_Ar_), 154.15 (C=O), 192.29 (C-S-); HRMS (ESI): *m*/*z* calcd for C_25_H_23_ClN_4_O_2_S 479.1308 [M + H]^+^, found 479.1306.

*1-(4-Fluorophenyl)-2-((5-(2-((4-methoxyphenyl)amino)ethyl)-4-phenyl-4H-1,2,4-triazol-3-yl)thio)ethanone* (**21**): Prepared according to *method C* from 2-bromo-4′-fluoroacetophenone (0.43 g). Yield 73%; white solid; m.p. 145–146 °C; IR (KBr) ν_max_ (cm^−1^): 3290 (NH); 1693 (C=O); ^1^H-NMR (DMSO-*d_6_*, 400 MHz) δ: 2.74 (t, *J* = 7.4 Hz, 2H, CH_2_C), 3.19 (t, *J* = 7.4 Hz, 2H, *CH_2_*NH), 3.61 (s, 3H, CH_3_O), 4.84 (s, 2H, SCH_2_), 5.22 (s, 1H, NH), 6.31 (d, *J* = 8.8 Hz, 2H, H_Ar2,6_), 6.63 (d, *J* = 8.8 Hz, 2H, H_Ar3,5_), 7.36–7.61 (m, 7H, H_Ar’, Ar’’_), 8.07–8.11 (m, 2H, H_Ar’’_); ^13^C-NMR (DMSO-*d_6_*, 101 MHz) δ: 24.7 (*CH_2_*C), 40.2 (SCH_2_), 41.3 (CH_2_NH), 55.3 (CH_3_O), 113.1, 114.6, 115.8, 115.9, 127.4, 130.0, 130.1, 131.4, 132.1, 132.9, 142.2, 149.3, 150.8 (C_Ar_), 154.1 (C=O), 191.8 (C-S-); HRMS (ESI): *m*/*z* calcd for C_25_H_23_FN_4_O_2_S 463.1604 [M + H]^+^, found 463.1601.

*2-((5-(2-((4-Methoxyphenyl)amino)ethyl)-4-phenyl-4H-1,2,4-triazol-3-yl)thio)-1-(4-nitrophenyl)ethanone* (**22**): Prepared according to *method C* from 2-bromo-4′-nitroacetophenone (0.49 g), recrystallized from propan-2-ol. Yield 88 %; brown solid; m.p. 169–170 °C IR (KBr) ν_max_ (cm^−1^): 3306 (NH), 1698 (C=O); ^1^H-NMR (DMSO-*d_6_*, 400 MHz) δ: 2.74 (t, *J* = 7.4 Hz, 2H, CH_2_C), 3.19 (t, *J* = 7.0 Hz, 2H, *CH_2_*NH), 3.61 (s, 3H, CH_3_O), 4.89 (s, 2H, SCH_2_), 5.22 (s, 1H, NH), 6.31 (d, *J* = 8.8 Hz, 2H, H_Ar2,6_), 6.63 (d, *J* = 8.8 Hz, 2H, H_Ar3,5_), 7.46–7.49 (m, 2H, H_Ar’_), 7.60–7.62 (m, 3H, H_Ar’_), 8.23 (d, *J* = 8,7 Hz, 2H, H_Ar’’_), 8.36 (d, *J* = 8.7 Hz, 2H, H_Ar’’_); ^13^C-NMR (DMSO-*d_6_*, 101 MHz) δ: 24.7 (*CH_2_*C), 40.2 (CH_2_NH), 41.3 (SCH_2_), 55.3 (CH_3_O), 113.1, 114.6, 123.9, 127.4, 129.8, 130.0, 130.1, 132.9, 140.1, 142.2, 149.1, 150.1, 150.8 (C_Ar_), 154.2 (C=O), 192.7 (C-S-); HRMS (ESI): *m*/*z* calcd for C_25_H_23_N_5_O_4_S 490.1549 [M + H]^+^, found 490.1544.

*1-(4-Hydroxyphenyl)-2-((5-(2-((4-methoxyphenyl)amino)ethyl)-4-phenyl-4H-1,2,4-triazol-3-yl)thio)ethanone* (**23**): Prepared according to *method C* from 2-bromo-4’-hydroxyacetophenone (0.43 g). Yield 84%; white solid; m.p. 198–199 °C; IR (KBr) ν_max_ (cm^−1^): 3380, 3300 (OH, NH), 1649 (C=O); ^1^H-NMR (DMSO-*d_6_*, 400 MHz) δ: 2.74 (t, *J* = 7.4 Hz, 2H, CH_2_C), 3.20 (q, *J* = 6.8, 13.0 Hz, 2H, *CH_2_*NH), 3.61 (s, 3H, CH_3_O), 4.76 (s, 2H, SCH_2_), 5.23 (t, *J* = 5.6 Hz, 1H, NH), 6.32 (d, *J* = 8.8 Hz, 2H, H_Ar2,6_), 6.64 (d, *J* = 8.8 Hz, 2H, H_Ar3,5_), 6.88 (d, *J* = 8.7 Hz, 2H, H_Ar’_’), 7.46–7.61 (m, 5H, H_Ar’_), 7.88 (d, *J* = 8.7 Hz, 2H, H_Ar’’_), 10.58 (s, 1H, OH); ^13^C-NMR (DMSO-*d_6_*, 101 MHz) δ: 24.7 (*CH_2_*C), 40.2 (CH_2_NH), 41.3 (SCH_2_), 55.3 (CH_3_O), 113.1, 114.6, 115.4, 126.7, 127.4, 129.9, 130.0, 131.1, 133.0, 142.2, 149.6, 150.8, 154.1 (C_Ar_), 162.7 (C=O), 191.1 (C-S-); HRMS (ESI): *m*/*z* calcd for C_25_H_24_N_4_O_3_S 461.1647 [M + H]^+^, found 461.1653.

*2-((5-(2-((4-Methoxyphenyl)amino)ethyl)-4-phenyl-4H-1,2,4-triazol-3-yl)thio)-1-(naphthalen-2-yl)ethanone* (**24**): Prepared according to *method C* from 2-bromo-2′-acetonaphthone (0.50 g). Yield 91%; yellow solid; m.p. 154–155 °C; IR (KBr) ν_max_ (cm^−1^): 3283 (NH); 1688 (C=O); ^1^H-NMR (DMSO-*d_6_*, 400 MHz) δ: 2.74 (t, *J* = 7.4 Hz, 2H, CH_2_C), 3.19 (q, *J* = 6.8, 14.0 Hz, 2H, *CH_2_*NH), 3.61 (s, 3H, CH_3_O), 5.01 (s, 2H, SCH_2_), 5.23 (t, *J* = 5.7 Hz, 1H, NH), 6.31 (d, *J* = 8.8 Hz, 2H, H_Ar2,6_), 6.63 (d, *J* = 8.8 Hz, 2H, H_Ar3,5_), 7.47–8.76 (m, 12H, H_Ar’, Ar’’_); ^13^C-NMR (DMSO-*d_6_*, 101 MHz) δ: 24.7 (*CH_2_*C), 40.3 (CH_2_NH), 41.3 (SCH_2_), 55.3 (CH_3_O), 113.1, 114.6, 123.7, 127.1, 127.4, 127.7, 128.4, 128.9, 129.7; 130.0, 130.1, 130.6, 132.1, 132.6, 133.0, 142.2, 149.4, 150.8 (C_Ar_), 154.1 (C=O), 193.1 (C-S-); (HRMS (ESI): *m*/*z* calcd for C_29_H_26_N_4_O_2_S 495.1854 [M + H]^+^, found 495.1849.

*N-(4-Methoxyphenyl)-N-(2-(5-((2-oxo-2-(p-tolyl)ethyl)thio)-4-phenyl-4H-1,2,4-triazol-3-yl)ethyl)acetamide* (**25**): A mixture of triazolethione **18** (0.2 g, 0.44 mmol) and acetyl chloride (10 mL) was heated at reflux for 1 h. The reaction mixture was poured into a water-ice mixture. Precipitate formed was filtered off, washed with water, and recrystallized from propan-2-ol. Yield 96%; white solid; m.p. 163–164 °C; IR (KBr) ν_max_ (cm^−1^): 1651, 1510 (C=O); ^1^H-NMR (DMSO-*d_6_*, 400 MHz) δ: 1.61 (s, 3H, CH_3_); 2.38 (s, 3H, CH_3_), 2.82 (t, *J* = 6.8 Hz, 2H, CH_2_C), 3.66 (t, *J* = 6.8 Hz, 2H, CH_2_N); 3.77 (s, 3H, CH_3_O), 4.92 (s, 2H, SCH_2_), 6.94 (d, *J* = 8.8 Hz, 2H, H_Ar2,6_), 7.08 (d, *J* = 8.8 Hz, 2H, H_Ar3,5_), 7.35–7.43 (m, 4H, H_Ar’, Ar’’_), 7.56–7.63 (m, 3H, H_Ar’_), 7.91 (d, *J* = 8.0 Hz, 2H, H_Ar’’_); ^13^C-NMR (DMSO-*d_6_*, 101 MHz) δ: 21.3 (CH_3_), 22.3 (CH_3_), 23.2 (*CH_2_*C), 40.5 (SCH_2_), 46.2 (CH_2_N), 55.4 (CH_3_O), 114.8, 127.2, 128.6, 129.4, 130.2, 130.6, 132.1, 132.7, 135.2, 144.5, 150.8, 153.5 (C_Ar_), 158.5, 169.6 (C=O), 192.4 (C-S-); HRMS (ESI): *m*/*z* calcd for C_28_H_28_N_4_O_3_S 501.1960 [M + H]^+^, found 501.1963.

*N-(2,5-Dimethyl-1H-pyrrol-1-yl)-3-((4-methoxyphenyl)amino)propanamide* (**26**): A mixture of propanehydrazide **1** (1.05 g, 5 mmol), propan-2-ol (75 mL), hexane-2,5-dione (0.69 g, 6 mmol), and acetic acid (1.5 mL) was heated at reflux for 20 h. Afterwards cold water (25 mL) was added. Precipitate formed was filtered off and recrystallized from ethanol. Yield 93%; white solid; m.p. 119–120 °C; IR (KBr) ν_max_ (cm^−1^): 3346, 2843 (NH), 1663 (C=O); ^1^H-NMR (DMSO-*d_6_*, 400 MHz) δ: 1.98, 2.02 (2s, 6H, 2CH_3_), 2.54 (t, *J* = 6.9 Hz, 2H, CH_2_CO), 3.32 (t, *J* = 6.9 Hz, 2H, *CH_2_*NH), 3.65 (s, 3H, CH_3_O), 5.23 (s, 1H, NH), 5.63, 5.71 (2s, 2H, 2CH), 6.54–6.78 (m, 4H, H_Ar_), 10.60 (s, 1H, NH); ^13^C-NMR (DMSO-*d_6_*, 101 MHz) δ: 10.9 (СH_3_), 33.3 (*CH_2_*CO), 40.1 (CH_2_NH), 55.2 (CH_3_O), 102.8, 113.3, 114.6, 126.7, 142.5, 150.9 (C_Ar_), 170.4 (C=O); HRMS (ESI): *m*/*z* calcd for C_16_H_21_N_3_O_2_ 289.1790 [M + H]^+^, found 289.1790.

#### 3.2.5. General Procedure for the Synthesis of Compounds **27a**,**b**

To propanehydrazide **1** (2.09 g, 10 mmol) dissolved in methanol (30 mL), the corresponding isatin (11 mmol) dissolved in methanol (15 mL) and glacial acetic acid (2–3 drops) were added. The reaction mixture was heated at reflux for 10–15 min until precipitate formed. The precipitate was filtered off while hot and recrystallized from DMF/H_2_O mixture.

*3-((4-Methoxyphenyl)amino)-N’-(2-oxoindolin-3-ylidene)propanehydrazide* (**27a**): Prepared from isatin (1.62 g). Yield 72%; yellow solid; m.p. 203–204 °C; IR (KBr) ν_max_ (cm^−1^): 1694, 1728 (C=O), 3389, 3342, 3262 (NH); ^1^H-NMR (DMSO-*d_6_*, 400 MHz) δ: 3.01–3.34 (m, 4H, CH_2_CO+ *CH_2_*NH), 3.63 (s, 3H, CH_3_O), 5.26 (s, 1H, NHAr), 6.55–7.48 (m, 8H, H_Ar, Ar‘_), 11.23 (s, 1H, NHC), 12.54 (s, 0.7H, NHCO), 12.98 (s, 0.3H, NHCO); ^13^C-NMR (DMSO-*d_6_*, 101 MHz) δ: 31.2 (*CH_2_*CO), 40.2 (CH_2_NH), 55.3 (CH_3_O), 111.1, 113.3, 114.6, 118.8, 119.8, 122.5, 131.4, 133.9, 142.3, 142.7, 150.9 (C_Ar_), 162.5, 173.9 (C=O); HRMS (ESI): *m*/*z* calcd for C_18_H_18_N_4_O_3_ 339.1457 [M + H]^+^, found 339.1467.

*N’-(1-Benzyl-2-oxoindolin-3-ylidene)-3-((4-methoxyphenyl)amino)propanehydrazide* (**27b**): Prepared from *N*-benzylisatin (2.61 g). Yield 78%; yellow solid; m.p. 123–124 °C; IR (KBr) ν_max_ (cm^−1^): 1678, 1614 (C=O), 3384, 3219 (NH); ^1^H-NMR (DMSO-*d_6_*, 400 MHz) δ: 2.68–3.43 (m, 4H, CH_2_CO + *CH_2_*NH), 3.64 (s, 3H, CH_3_O), 4.90–4.98 (m, 2H, CH_2_), 5.82 (s, 1H, NHAr), 6.60–7.61 (m, 13H, H_Ar, Ar′, Ar″_), 11.29, 12.48 (2s, 0.7H, NHCO), 12.91 (s, 0.3H, NHCO); ^13^C-NMR (DMSO-*d_6_*, 101 MHz) δ: 31.1 (*CH_2_*CO), 39.9 (CH_2_), 42.5 (CH_2_NH), 55.3 (CH_3_O), 110.4, 113.8, 114.6, 114.8, 119.3, 120.2, 123.2, 127.4, 127.6, 128.7, 131.3, 135.7, 142.0, 142.5, 151.3 (C_Ar_), 160.6, 173.8 (C=O); HRMS (ESI): *m*/*z* calcd for C_25_H_24_N_4_O_3_ 429.1926 [M + H]^+^, found 429.1929.

#### 3.2.6. General Procedure for the Synthesis of Compounds **28**–**37**

To propanehydrazide **1** (0.63 g, 3 mmol) dissolved in methanol (20 mL), corresponding acetophenone (3 mmol) and acetic acid (4 drops; in the case of compounds **29**, **30**, **32**, **33**, and **36**) were added. The reaction mixture was heated at 95 °C for 4–24 h. Precipitate formed was filtered off, washed with water, and recrystallized from methanol. 

*N’-(1-(Furan-2-yl)ethylidene)-3-((4-methoxyphenyl)amino)propanehydrazide* (**28**): Prepared from 2-acetylfuran (0.33 g, 0.30 mL) by heating the reaction mixture for 22 h. Yield 76%; white solid; m.p. 142–143 °C; IR (KBr) ν_max_ (cm^−1^): 3361, 3389 (NH), 1679 (C=O); ^1^H-NMR (DMSO-*d_6_*, 400 MHz) δ: 2.16 (s, 3H, CH_3_), 2.56 (t, *J* = 7.2 Hz, 0.8H, CH_2_CO), 2.85 (t, *J* = 7.2 Hz, 1.2H, CH_2_CO), 3.26 (t, *J* = 7.2 Hz, 2H, *CH*_2_NH), 3.63 (s, 3H, CH_3_O), 5.15, 5.20 (2t, *J* = 4.8 Hz, 1H, NHAr), 6.53–6.89 (m, 7H, H_Ar, furan_), 10.30 (s, 0.4H, NH), 10.40 (s, 0.6H, NH); ^13^C-NMR (DMSO-*d_6_*, 101 MHz) δ: 13.2, 13.5 (CH_3_), 32.6, 34.1 (*CH_2_*CO), 48.6 (CH_2_NH), 55.3 (CH_3_O), 110.6, 111.8, 113.1, 114.6, 139.6, 142.8, 144.2, 150.7 (C_Ar_), 151.9 (C=N), 173.7 (C=O); HRMS (ESI): *m*/*z* calcd for C_16_H_19_N_3_O_3_ 302.1504 [M + H]^+^, found 302.1501.

*3-((4-Methoxyphenyl)amino)-N’-(1-(thiophen-2-yl)ethylidene)propanehydrazide* (**29**). (*Z/E* isomeric mixture, 60 : 40%): Prepared from 2-acetylthiophene (0.38 g, 0.32 mL) by heating the reaction mixture for 12 h. Yield 69%; white solid; m.p. 160–162 °C; IR (KBr) ν_max_ (cm^−1^): 3363, 3389 (NH), 1679 (C=O); ^1^H-NMR (DMSO-*d_6_*, 400 MHz) δ: 2.26 (s, 3H, CH_3_), 2.56 (t, *J* = 8.0 Hz, 0.8H, CH_2_CO), 2.86 (t, *J* = 8.0 Hz, 1.2H, CH_2_CO), 3.28 (t, *J* = 8.0 Hz, 2H, *CH_2_*NH), 3.63 (s, 3H, CH_3_O), 5.17 (s, 1H, NH), 6.55–6.74 (m, 4H, H_Ar_), 7.05–7.07 (m, 1H, CH), 7.40–7.56 (m, 2H, CH), 10.36 (s, 0.4H, NH), 10.51 (s, 0.6H, NH); ^13^C-NMR (DMSO-*d_6_*, 101 MHz) δ: 13.9, 14.4 (CH_3_), 32.6, 34.1 (*CH_2_*CO), 40.2 (CH_2_NH), 55.3 (CH_3_O), 113.2, 113.4, 114.6, 127.1, 127.4, 127.6, 127.7, 128.0, 128.6 (C_thiophene_), 142.8, 142.9, 143.5, 143.6, 143.9, 147.9, 150.7, 150.9 (C_Ar_), 167.6 (C=N), 173.6 (C=O); HRMS (ESI): *m*/*z* calcd for C_16_H_19_N_3_O_2_S 318.1276 [M + H]^+^, found 318.1273.

*3-((4-Methoxyphenyl)amino)-N’-(1-(thiophen-2-yl)propylidene)propanehydrazide* (**30**). (*Z/E* isomeric mixture, 60 : 40%): Prepared from 2-propionylthiophene (0.42 g, 0.37 mL) by heating the reaction mixture for 19 h. Yield 86%); white solid; m.p. 143–145 °C; IR (KBr) ν_max_ (cm^−1^): 3345, 3390 (NH), 1675 (C=O); ^1^H-NMR (DMSO-*d_6_*, 400 MHz) δ: 1.05 (t, 3H, *J* = 8.0 Hz, *CH_3_*CH_2_), 2.58 (t, 0.8H, *J* = 8.0 Hz, CH_2_CO), 2.77 (qui, 2H, *J* = 8.0 Hz, CH_3_*CH_2_*), 2.87 (t, 1.2H, Hz, CH_2_CO), 3.28 (qui, 2H, *J* = 8.0 Hz, *CH_2_*NH), 3.63, 3.64 (2s, 3H, CH_3_O), 5.17, 5.18 (2s, 1H, NH), 6.55–6.75 (m, 4H, H_Ar_), 7.04 (t, 1H, *J* = 4.0 Hz, CH), 7.39–7.56 (m, 2H, CH), 10.42 (s, 0.4H, NH), 10.65 (s, 0.6H, NH); ^13^C-NMR (DMSO-*d_6_*, 101 MHz) δ: 11.1 (*CH_3_*CH_2_), 20.1, 20.4 (CH_3_*CH_2_*), 32.7, 34.1 (*CH_2_*CO), 40.3 (CH_2_NH), 55.3 (CH_3_O), 113.2, 113.4, 114.6, 126.6, 127.2, 127.5, 127.7, 127.9, 128.5 (C_thiophene_), 142.7, 142.8, 142.8, 142.9, 148.3, 150.7, 150.9, 151.7 (C_Ar_), 167.7 (C=N), 173.7 (C=O); HRMS (ESI): *m*/*z* calcd for C_17_H_21_N_3_O_2_S 332.1432 [M + H]^+^, found 332.1431.

*3-((4-Methoxyphenyl)amino)-N’-(1-phenylethylidene)propanehydrazide* (**31**): Prepared from acetophenone (0.36 g, 0.35 mL) by heating the reaction mixture for 4h. Yield 75%; white solid; m.p. 155–156 °C; IR (KBr) ν_max_ (cm^−1^): 3385, 3358 (NH), 1680 (C=O); ^1^H-NMR (DMSO-*d_6_*, 400 MHz) δ: 2.25 (s, 3H, CH_3_), 2.59 (t, *J* = 6.8 Hz, 0.8H, CH_2_CO), 2.93 (t, *J* = 6.8 Hz, 1.2H, CH_2_CO), 3.24–3.32 (m, 2H, *CH_2_*NH), 3.63, 3.64 (2s, 3H, CH_3_O), 5.20 (s, 1H, NH), 6.52–6.77 (m, 4H, H_Ar_), 7.33–7.82 (m, 5H, H_acetophenone_), 10.39 (s, 0.4H, NH), 10.51 (s, 0.6H, NH), ^13^C-NMR (DMSO-*d_6_*, 101 MHz) δ: 13.6, 14.1 (CH_3_), 32.7, 34.1 (*CH_2_*CO), 40.3 (CH_2_NH), 55.3 (CH_3_O), 113.2, 113.4, 114.6, 126.0, 126.3, 128.3, 128.3, 128.9, 129.1 (C_acetophenone_), 138.3, 142.8, 147.3, 150.7, 150.9 (C_Ar_), 167.9 (C=N), 173.9 (C=O); HRMS (ESI): *m*/*z* calcd for C_18_H_21_N_3_O_2_ 312.1712 [M + H]^+^, found 312.1710.

*3-((4-Methoxyphenyl)amino)-N’-(1-phenylpropylidene)propanehydrazide* (**32**) (*Z/E* isomeric mixture, 60 : 40%): Prepared from propiophenone (0.4 g, 0.39 mL) by heating the reaction mixture for 12 h. Yield 75%; white solid; m.p. 136–138 °C; IR (KBr) ν_max_ (cm^−1^): 3345, 3395 (NH), 1676 (C=O); ^1^H-NMR (DMSO-*d_6_*, 400 MHz) δ: 1.01 (t, *J* = 8.0 Hz, 3H, *CH_3_*CH_2_), 2.61 (t, *J* = 8.0 Hz, 0.8H, CH_2_CO), 2.75–2.83 (m, 2H, CH_3_*CH_2_*), 2.95 (t, *J* = 8.0 Hz, 1.2H, CH_2_CO), 3.25–3.33 (m, 2H, *CH_2_*NH), 3.63, 3.64 (CH_3_O), 5.20 (s, 1H, NH), 6.51–6.78 (m, 4H, H_Ar_), 7.34–7.81 (m, 5H, H_acetophenone_), 10.49 (s, 0.4H, NH), 10.65 (s, 0.6H, NH); ^13^C-NMR (DMSO-*d_6_*, 101 MHz) δ: 10.8 (*CH_3_*CH_2_), 19.2, 19.5 (CH_3_*CH_2_*), 32.7, 34.2 (*CH_2_*CO), 40.3 (CH_2_NH), 55.3 (CH_3_O), 113.2, 113.4, 114.6, 126.1, 126.4, 128.4, 128.5, 128.9, 129.0 (C_acetophenone_), 137.1, 142.8, 142.9, 150.7, 150.9, 151.5, 154.8 (C_Ar_), 168.1 (C=N), 174.1 (C=O); HRMS (ESI): *m*/*z* calcd for C_19_H_23_N_3_O_2_ 326.1868 [M + H]^+^, found 326.1864.

*N’-(1-(4-Aminophenyl)ethylidene)-3-((4-methoxyphenyl)amino)propanehydrazide* (**33**) (*Z/E* isomeric mixture, 60 : 40%): Prepared from 4′-aminoacetophenone (0.41 g) by heating the reaction mixture for 24 h. Yield 68%; white solid; m.p. 137–138 °C; IR (KBr) ν_max_ (cm^−1^): 3464, 3363, 3332 (NH, NH_2_), 1660 (C=O); ^1^H-NMR (DMSO-*d_6_*, 400 MHz) δ: 2.13 (s, 3H, CH_3_), 2.54 (t, *J* = 6.8 Hz, 0.8H, CH_2_CO), 2.89 (t, *J* = 6.8 Hz, 1.2H, CH_2_CO), 3.26 (t, *J* = 6.8 Hz, 2H, *CH_2_*NH), 3.64 (s, 3H, CH_3_O), 5.11–5.22 (m, 1H, NH), 5.42 (s, 2H, NH_2_), 6.52–6.59 (m, 4H, H_Ar_), 6.68–6.75 (m, 2H, H_acetophenone_), 7.45–7.53 (m, 2H, H_acetophenone_), 10.14 (s, 0.4H, NH), 10.21 (s, 0.6H, NH); ^13^C-NMR (DMSO-*d_6_*, 101 MHz) δ: 13.2, 13.7 (CH_3_), 32.7, 34.2 (*CH_2_*CO), 39.7, 40.2 (CH_2_NH), 55.3 (CH_3_O), 113.1, 113.2, 113.3, 114.6, 125.3, 125.5, 127.1, 127.5 (C_acetophenone_), 142.9, 148.2, 149.9, 150.1, 150.7, 150.8 (C_acetophenone_), 152.3 (C_Ar_), 167.3 (C=N), 173.4 (C=O); HRMS (ESI): *m*/*z* calcd for C_18_H_22_N_4_O_2_ 327.1821 [M + H]^+^, found 327.1842.

*N’-(1-(3-Aminophenyl)ethylidene)-3-((4-methoxyphenyl)amino)propanehydrazide* (**34**): Prepared from 3′-aminoacetophenone (0.41 g) by heating the reaction mixture for 24 h. Yield 71%; white solid; m.p. 109–110 °C; IR (KBr) ν_max_ (cm^−1^): 3464, 3379, 3358 (NH, NH_2_), 1679 (C=O); ^1^H-NMR (DMSO-*d_6_*, 400 MHz) δ: 2.17 (s, 3H, CH_3_), 2.57 (t, *J* = 6.8 Hz, 0.8H, CH_2_CO), 2.91 (t, *J* = 6.8 Hz, 1.2H, CH_2_CO), 3.28 (t, *J* = 6.8 Hz, 2H, *CH_2_*NH), 3.63, 3.64 (2s, 3H, CH_3_O), 5.13 (s, 3H, NH+NH_2_), 6.51–7.08 (m, 8H, H_Ar, acetophenone_), 10.27 (s, 0.4H, NH), 10.39 (s, 0.6H, NH); ^13^C-NMR (DMSO-*d_6_*, 101 MHz) δ: 13.8, 14.2 (CH_3_), 32.8, 34.2 (*CH_2_*CO), 40.2, 40.3 (CH_2_NH), 55.3, 55.3 (CH_3_O), 111.5 (C-2_acetophenone_), 113.2, 113.4, 113.9 (C-4_acetophenone_), 114.3, 114.7, 115.0 (C-6_acetophenone_), 128.7, 128.7, 138.9, 139.0 (C-5_acetophenone_), 142.9 (C-1_acetophenone_), 148.2, 148.5, 148.6 (C-3_acetophenone_), 150.7, 150.9, 151.8 (C_Ar_), 167.8 (C=N), 173.8 (C=O); HRMS (ESI): *m*/*z* calcd for C_18_H_22_N_4_O_2_ 327.1821 [M + H]^+^, found 327.1835.

*N’-(1-(3,4-Dimethoxyphenyl)ethylidene)-3-((4-methoxyphenyl)amino)propanehydrazide* (**35**): Prepared from 3′,4′-dimethoxyacetophenone (0.54 g) by heating the reaction mixture for 48 h. Yield 27%; white solid; m.p. 73–74 °C; IR (KBr) ν_max_ (cm^−1^): 3465, 3366 (NH), 1675 (C=O); ^1^H-NMR (DMSO-*d_6_*, 400 MHz) δ: 2.21, 2.22 (2s, 3H, CH_3_), 2.57 (t, *J* = 6.8 Hz, 0.8H, CH_2_CO), 2.92 (t, *J* = 6.8 Hz, 1.2H, CH_2_CO), 3.24–3.32 (m, 2H, *CH_2_*NH), 3.62, 3.64 (2s, 3H, CH_3_O), 3.72, 3.78, 3.81, 3.84 (4s, 6H, 2CH_3_O), 5.15–5.23 (m, 1H, NH), 6.50–6.75 (m, 4H, H_Ar_), 6.92–7.65 (m, 3H, H_acetophenone_), 10.41 (s, 1H, NH); ^13^C-NMR (DMSO-*d_6_*, 101 MHz) δ: 13.5, 14.0 (CH_3_), 32.8, 34.2 (*CH*_2_CO), 39.6, 40.4 (CH_2_NH), 55.2, 55.3, 55.3, 55.4, 55.5, 55.7 (CH_3_O), 109.1, 109.2, 110.2, 110.8, 111.0, 111.2 (C-2,5_acetophenone_), 113.2, 113.3, 114.6, 114.6, 119.2, 119.6 (C-6_acetophenone_), 129.9, 130.9, 131.0 (C-1_acetophenone_), 142.9, 148.4, 148.5, 148.5 (C-3_acetophenone_), 149.8, 150.0, 150.7, 150.8, 151.3, 153.0 (C-4_acetophenone_) (C_Ar_), 167.7 (C=N), 173.8 (C=O); HRMS (ESI): *m*/*z* calcd for C_20_H_25_N_3_O_4_ 372.1923 [M + H]^+^, found 372.1921.

*3-((4-Methoxyphenyl)amino)-N’-(1-(naphthalen-1-yl)ethylidene)propanehydrazide* (**36**). (*Z/E* isomeric mixture, 60 : 40%): Prepared from 1-acetonaphthone (0.51 g, 0.46 mL) by heating the reaction mixture for 5 h. Yield 65%; white solid; m.p. 152–154 °C; IR KBr) ν_max_ (cm^−1^): 3337, 3290 (NH), 1667 (C=O); ^1^H-NMR (DMSO-*d_6_*, 400 MHz) δ: 2.37, 2.38 (2s, 3H, CH_3_), 2.64 (t, *J* = 6.8 Hz, 0.8H, CH_2_CO), 2.79 (t, *J* = 6.8 Hz, 1.2H, CH_2_CO), 3.19–3.32 (m, 2H, *CH_2_*NH), 3.57, 3.65 (2s, 3H, CH_3_O), 5.15 (s, 0.6H, NH), 5.22 (s, 0.4H, NH), 6.43–6.76 (m, 4H, H_Ar_), 7.48–7.58 (m, 4H, H_acetonaphthone_), 7.90–8.21 (m, 3H, H_acetonaphthone_), 10.51 (s, 0.4H, NH), 10.61 (s, 0.6H, NH); ^13^C-NMR (DMSO-*d_6_*, 101 MHz) δ: 18.8, 19.1 (CH_3_), 32.9, 34.2 (*CH_2_*CO), 40.2 (CH_2_NH), 55.2, 55.3 (CH_3_O), 113.1, 113.4, 114.4, 114.7, 125.2, 125.3, 125.3, 125.6, 125.9, 126.0, 126.5, 126.6, 128.4, 128.6, 128.7 (C_acetonaphthone_), 130.2, 133.4, 137.7, 137.9, 142.6, 142.9, 149.4, 150.6, 150.9 (C_Ar_), 153.5 (C=N), 167.9, 173.9 (C=O); HRMS (ESI): *m*/*z* calcd for C_22_H_23_N_3_O_2_ 362.1868 [M + H]^+^, found 362.1868.

*3-((4-Methoxyphenyl)amino)-N’-(1-(naphthalen-1-yl)ethylidene)propanehydrazide* (**36**). (*Z/E* isomeric mixture, 60 : 40%): Prepared from 1-acetonaphthone (0.51 g, 0.46 mL) by heating the reaction mixture for 5 h. Yield 65%; white solid; m.p. 152–154 °C; IR KBr) ν_max_ (cm^−1^): 3337, 3290 (NH), 1667 (C=O); ^1^H-NMR (DMSO-*d_6_*, 400 MHz) δ: 2.37, 2.38 (2s, 3H, CH_3_), 2.64 (t, *J* = 6.8 Hz, 0.8H, CH_2_CO), 2.79 (t, *J* = 6.8 Hz, 1.2H, CH_2_CO), 3.19–3.32 (m, 2H, *CH_2_*NH), 3.57, 3.65 (2s, 3H, CH_3_O), 5.15 (s, 0.6H, NH), 5.22 (s, 0.4H, NH), 6.43–6.76 (m, 4H, H_Ar_), 7.48–7.58 (m, 4H, H_acetonaphthone_), 7.90–8.21 (m, 3H, H_acetonaphthone_), 10.51 (s, 0.4H, NH), 10.61 (s, 0.6H, NH); ^13^C-NMR (DMSO-*d_6_*, 101 MHz) δ: 18.8, 19.1 (CH_3_), 32.9, 34.2 (*CH_2_*CO), 40.2 (CH_2_NH), 55.2, 55.3 (CH_3_O), 113.1, 113.4, 114.4, 114.7, 125.2, 125.3, 125.3, 125.6, 125.9, 126.0, 126.5, 126.6, 128.4, 128.6, 128.7 (C_acetonaphthone_), 130.2, 133.4, 137.7, 137.9, 142.6, 142.9, 149.4, 150.6, 150.9 (C_Ar_), 153.5 (C=N), 167.9, 173.9 (C=O); HRMS (ESI): *m*/*z* calcd for C_22_H_23_N_3_O_2_ 362.1868 [M + H]^+^, found 362.1868.

*3-((4-Methoxyphenyl)amino)-N’-(1-(naphthalen-2-yl)ethylidene)propanehydrazide* (**37**): Prepared from 2-acetonaphthone (0.51 g) by heating the reaction mixture for 16 h. Yield 69%; white solid; m.p. 141–142 °C; IR (KBr) ν_max_ (cm^−1^): 3367, 3310 (NH) 1662 (C=O); ^1^H-NMR (DMSO-*d_6_*, 400 MHz) δ: 2.37 (s, 3H, CH_3_), 2.64 (t, *J* = 6.8 Hz, 0.8H, CH_2_CO), 3.01 (t, *J* = 6.8 Hz, 1.2H, CH_2_CO), 3.27–3.39 (m, 2H, *CH_2_*NH), 3.61, 3.64 (2s, 3H, CH_3_O), 5.23 (s, 1H, NH), 6.53–6.78 (m, 4H, H_Ar_), 7.48–8.27 (m, 7H, H_acetonaphthone_), 10.50 (s, 0.4H, NH), 10.62 (s, 0.6H, NH); ^13^C-NMR (DMSO-*d_6_*, 101 MHz) δ: 13.4, 13.8 (CH_3_), 32.8, 34.3 (*CH_2_*CO), 39.6, 40.3 (CH_2_NH); 55.2, 55.3 (CH_3_O), 113.2, 113.4, 114.6, 123.4, 123.7, 125.9, 126.2, 126.4, 126.7, 126.7, 127.5, 127.6, 127.7, 128.4, 132.8, 132.8, 133.1, 133.2, 135.6, 135.7 (C_acetonaphthone_); 142.9, 142.9, 150.6, 150.7, 150.9 (C_Ar_), 153.5 (C=N), 168.1, 174.0 (C=O); HRMS (ESI): *m*/*z* calcd for C_22_H_23_N_3_O_2_ 362.1868 [M + H]^+^, found 362.1868.

*N-(4-Methoxyphenyl)-N-(3-oxo-3-(2-(1-phenylethylidene)hydrazinyl)propyl)acetamide* (**38**): To propanehydrazide **31** (0.19 g, 0.6 mmol) dissolved in methanol (5 mL), acetic anhydride (5 mL) was added and the reaction mixture was heated at reflux for 2 h. Afterwards water (10 mL) was added. Precipitate formed was filtered off, washed with methanol, and recrystallized from methanol. Yield 59 %; white solid; m.p. 145–146 °C; IR (KBr) ν_max_ (cm^−1^): 3176 (NH) 1658; 1607 (C=O); ^1^H-NMR (DMSO-*d_6_*, 400 MHz) δ: 1.70 (s, 3H, CH_3_), 2.19, 2.23 (2s, 3H, CH_3_), 2.54 (t, *J* = 6.8 Hz, 0.8H, CH_2_CO), 2.84 (t, *J* = 6.8 Hz, 1.2H, CH_2_CO), 3.77 (s, 3H, CH_3_O), 3.87 (t, 2H, CH_2_N), 6.93–7.78 (m, 9H, H_Ar_), 10.37 (s, 0.4H, NH), 10.47 (s, 0.6H, NH); ^13^C-NMR (DMSO-*d_6_*, 101 MHz) δ: 13.4, 14.1 (CH_3_), 22.5 (CH_3_), 31.5, 32.8 (CH_2_CO), 44.9, 45.1 (CH_2_N), 55.3 (CH_3_O), 114.7, 125.9, 126.3, 128.3, 128.9, 129.4 (C_acetophenone_), 135.4, 138.0, 138.3, 147.2, 158.4 (C_Ar_), 167.1, 169.2, 169.2, 173.1 (C=O); HRMS (ESI): *m*/*z* calcd for C_20_H_23_N_3_O_3_ 354.1817 [M + H]^+^, found 354.1844.

*N-(1,3-Dioxoisoindolin-2-yl)-3-((4-methoxyphenyl)amino)propanamide* (**39**): To propanehidrazyde **1** (0.63 g, 3 mmol) dissolved in 1,4-dioxane (20 mL), phthalic anhydride (0.89 g, 6 mmol) was added and the reaction mixture was heated at reflux for 4 h. Afterwards Na_2_CO_3_ was added until pH 7. Precipitate formed was filtered off, washed with water, and recrystallized from methanol. Yield 81%; white solid; m.p. 147–148 °C; IR (KBr) ν_max_ (cm^−1^): 3331, 3290 (NH), 1664 (C=O); ^1^H-NMR (DMSO-*d_6_*, 400 MHz) δ: 2.46 (t, *J* = 6.8 Hz, 0.8H, CH_2_CO), 2.89 (t, *J* = 6.8 Hz, 1.2H, CH_2_CO), 3.25–3.30 (m, 2H, *CH_2_*NH), 3.64 (s, 3H, CH_3_O), 5.14–5.24 (m, 1H, NH), 6.54–6.75 (m, 4H, H_Ar_), 7.37–7.70 (m, 4H, H_phthal_), 11.32 (s, 0.6H, NH), 11.38 (s, 0.4H, NH); ^13^C-NMR (DMSO-*d_6_*, 101 MHz) δ: 32.2 (*CH_2_*CO), 34.3 (CH_2_NH); 55.3, 55.3 (CH_3_O), 113.2, 113.2, 114.6, 126.6, 126.9, 128.8, 129.7, 129.9, 134.3, 134.3, 142.7, 150.7 (C_Ar_), 167.3, 173.1 (C=O); HRMS (ESI): *m*/*z* calcd for C_18_H_17_N_3_O_4_ 340.1297 [M + H]^+^, found 340.1299. 

### 3.3. Evaluation of Antioxidant Activity

The free radical scavenging activity of compounds was screened by DPPH (1,1-diphenyl-2-picrylhydrazyl) radical scavenging assay [4]. Briefly, 1 mM solution of DPPH in ethanol was prepared, and this solution (1 mL) was added to the solutions of the tested compounds (1 mg/mL of DMSO). The mixture was shaken vigorously and allowed to stand at room temperature for 20 min. Afterwards, the absorbance was measured at 517 nm with a UV-200-RS spectrophotometer (MRC Ltd., Israel). The lower absorbance of the reaction mixture indicated a higher free radical scavenging activity. The capability to scavenge the DPPH radical was calculated according to the following equation:

(1) $$DPPH\;scavenging\;effect\;\left(\%\right)=\frac{A_{0}-A_{1}}{A_{0}}\;\times \;100$$

where *A*_0_—the absorbance of the control reaction and *A*_1_—the absorbance in the presence of the compounds.

### 3.4. Evaluation of Anticancer Activity

The anticancer activity of the synthesized compounds was evaluated using a 3-(4,5-dimethylthiazol-2-yl)-2,5-diphenyltetrazolium bromide (MTT, Sigma-Aldrich Co.) assay as described elsewhere [37]. Briefly, human glioblastoma U-87 and human triple-negative breast cancer cell line MDA-MB-231 were obtained from the American Type Culture Collection (ATCC, Manassas, VA, USA). Cell lines were grown in Dulbecco’s Modified Eagle’s Medium GlutaMAX (DMEM GlutaMAX) (Gibco, Carlsbad, CA, USA) supplemented with 10% FBS and 1% antibiotics (10,000 U/mL penicillin and 10 mg/mL streptomycin; Gibco) at 37 °C in a humidified atmosphere containing 5% CO_2_. Cell cultures were grown to 70% confluence and trypsinized with 0.125% TrypLE™ Express solution (Gibco) before passage. They were used until passage 20.

One hundred μL of cancer cells were seeded in 96-well plates in triplicate (5 × 10^3^ cells/well) and incubated at 37 °C for 24 h. Next day the tested compounds were added into cells at a concentration of 100 µM in triplicate. Final dimethylsulfoxide (DMSO, solvent of tested compounds) concentration in cells was 0.5%. Free medium without cells was used as a positive control. Cells treated with medium containing 0.5% DMSO served as a blank, or negative control. After 72 h incubation, the medium was aspirated from the plate/Fresh cell culture medium containing 0.5 mg/mL of MTT was added into each well and incubated for 4 h at 37 °C in a humidified atmosphere containing 5% CO_2_. Then the liquid was aspirated from the wells. Formazan crystals were dissolved in 100 μL of DMSO. Absorbance was measured at 570 nm and a reference wavelength of 630 nm using a multi-detection microplate reader Multiskan go (Thermo Fisher Scientific Oy, Ratastie, Finland). 

Compound effect on cell viability was calculated using the formula:

(2) $$Relative\;cell\;viability\;\left(\%\right)=\frac{A-A_{0}}{A_{NC}-A_{0}\;}\;\times \;100$$

where *A*—mean of absorbance of the tested compound, *A*_0_—mean of absorbance of blank (no cells, positive control) and *A_NC_*—mean of absorbance of a negative control (only cells, no treatment).

## 4. Conclusions

A series of novel 3-[(4-methoxyphenyl)amino]propanehydrazide derivatives were synthesized. Antioxidant activity of the synthesized compounds was screened by DPPH radical scavenging method. The antioxidant activity of *N*-(1,3-dioxoisoindolin-2-yl)-3-((4-methoxyphenyl)-amino)propanamide (**39**) and 3-((4-methoxyphenyl)amino)-*N’*-(1-(naphthalen-1-yl)ethylidene)-propanehydrazide (**36**) surpassed that of ascorbic acid *ca*. 1.4 times. 1-(4-Fluorophenyl)-2-((5-(2-((4-methoxyphenyl)amino)ethyl)-4-phenyl-4*H*-1,2,4-triazol-3-yl)thio)ethanone (**21**), which reduced cell viability at 100 µM concentration up to 19.6 ± 1.5%, has been identified as the most active compound against glioblastoma U-87 cell line.

## Figures and Tables

**Scheme 1:**
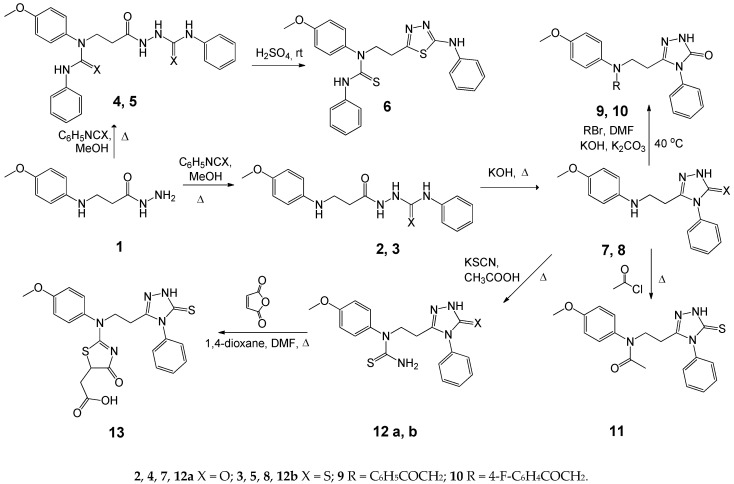
Synthesis of compounds **2**–**13**.

**Table 1:** DPPH scavenging activity of compounds **1**–**39**.

Compound	DPPH Scavenging Activity, %	Compound	DPPH Scavenging Activity, %
**1**	54.98	**21**	31.9
**2**	23.36	**22**	25.6
**3**	67.9	**23**	5.1
**4**	27.5	**24**	48.7
**5**	44.0	**25**	1.1
**6**	62.6	**26**	25.08
**7**	29.98	**27a**	66.67
**8**	45.1	**27b**	36.09
**9**	0	**28**	50.24
**10**	0	**29**	73.46
**11**	28.2	**30**	55.92
**12a**	16.36	**31**	33.18
**12b**	10.79	**32**	0
**13**	13.83	**33**	0
**14**	67.3	**34**	0
**15**	60.7	**35**	65.40
**16**	58.7	**36**	78.67
**17**	65.7	**37**	58.77
**18**	23.1	**38**	7.89
**19**	29.7	**39**	79.62
**20**	60.9	Ascorbic acid *	58.2

* Control.

## Supplementary Materials

The following are available online, Figures S1–S120 display ^1^H-NMR, ^13^C-NMR, and HRMS spectra of compounds **2**–**39**.
