# Supplementary material for: Antioxidant and Anticancer Activity of Novel Derivatives of 3-[(4-Methoxyphenyl)amino]propanehydrazide
Source: Molecules. 2020 Jun 29;25(13):2980. doi: 10.3390/molecules25132980 (PMC7412228; doi:10.3390/molecules25132980)
Supplement: Supplementary file 1 [file molecules-25-02980-s001.pdf]

Supplementary Material

Antioxidant and anticancer activity of novel derivatives of 3-[(4-methoxyphenyl)amino]propanehydrazide

Ingrida Tumosienė [1], Kristina Kantminienė [2,*], Arnas Klevinskas [1,2], Vilma Petrikaitė [3,4,5], Ilona Jonuškienė [1] and Vytautas Mickevičius [1]

[1] Department of Organic Chemistry, Kaunas University of Technology, Radvilėnų pl. 19, LT-50254 Kaunas, Lithuania; ingrida.tumosiene@ktu.lt (I.T.); ilona.jonuskiene@ktu.lt (I.J.); arnas.klevinskas@ktu.edu (A.K.); vytautas.mickevicius@ktu.lt (V.M.)
[2] Department of Physical and Inorganic Chemistry, Kaunas University of Technology, Radvilėnų pl. 19, LT-50254 Kaunas, Lithuania; kristina.kantminiene@ktu.lt
[3] Laboratory of Drug Targets Histopathology, Institute of Cardiology, Lithuanian University of Health Sciences, Sukilėlių pr. 13, LT-50162 Kaunas, Lithuania; vilmapetrikaite@gmail.com
[4] Institute of Physiology and Pharmacology, Faculty of Medicine, Lithuanian University of Health Sciences, A. Mickevičiaus g. 9, LT-44307 Kaunas, Lithuania; vilmapetrikaite@gmail.com
[5] Institute of Biotechnology, Life Sciences Center, Vilnius University, Saulėtekio al. 7, LT-10257 Vilnius, Lithuania; vilmapetrikaite@gmail.com
* Correspondence: kristina.kantminiene@ktu.lt; Ph: +370-37-300178

Table of contents

Figure S1. ^{1}H-NMR (400 MHz, DMSO-d$_6$) spectrum of **2**

Figure S2. ^{13}C-NMR (400 MHz, DMSO-d$_6$) spectrum of **2**

Figure S3. HRMS spectrum of **2**

Figure S4. ^{1}H-NMR (400 MHz, DMSO-d$_6$) spectrum of **3**

Figure S5. ^{13}C-NMR (400 MHz, DMSO-d$_6$) spectrum of **3**

Figure S6. HRMS spectrum of **3**

Figure S7. ^{1}H-NMR (400 MHz, DMSO-d$_6$) spectrum of **4**

Figure S8. ^{13}C-NMR (400 MHz, DMSO-d$_6$) spectrum of **4**

Figure S9. HRMS spectrum of **4**

Figure S10. ^{1}H-NMR (400 MHz, DMSO-d$_6$) spectrum of **5**

Figure S11. ^{13}C-NMR (400 MHz, DMSO-d$_6$) spectrum of **5**

Figure S12. HRMS spectrum of **5**

Figure S13. ^{1}H-NMR (400 MHz, DMSO-d$_6$) spectrum of **6**

Figure S14. ^{13}C-NMR (400 MHz, DMSO-d$_6$) spectrum of **6**

Figure S15. HRMS spectrum of **6**

Figure S16. ^{1}H-NMR (400 MHz, DMSO-d_6) spectrum of **7**

Figure S17. ^{13}C-NMR (400 MHz, DMSO-d_6) spectrum of **7**

Figure S18. HRMS spectrum of **7**

Figure S19. ^{1}H-NMR (400 MHz, DMSO-d_6) spectrum of **8**

Figure S20. ^{13}C-NMR (400 MHz, DMSO-d_6) spectrum of **8**

Figure S21. HRMS spectrum of **8**

Figure S22. ^{1}H-NMR (400 MHz, DMSO-d_6) spectrum of **9**

Figure S23. ^{13}C-NMR (400 MHz, DMSO-d_6) spectrum of **9**

Figure S24. HRMS spectrum of **9**

Figure S25. ^{1}H-NMR (400 MHz, DMSO-d_6) spectrum of **10**

Figure S26. ^{13}C-NMR (400 MHz, DMSO-d_6) spectrum of **10**

Figure S27. HRMS spectrum of **10**

Figure S28. ^{1}H-NMR (400 MHz, DMSO-d_6) spectrum of **11**

Figure S29. ^{13}C-NMR (400 MHz, DMSO-d_6) spectrum of **11**

Figure S30. HRMS spectrum of **11**

Figure S31. ^{1}H-NMR (400 MHz, DMSO-d_6) spectrum of **12a**

Figure S32. ^{13}C-NMR (400 MHz, DMSO-d_6) spectrum of **12a**

Figure S33. HRMS spectrum of **12a**

Figure S34. ^{1}H-NMR (400 MHz, DMSO-d_6) spectrum of **12b**

Figure S35. ^{13}C-NMR (400 MHz, DMSO-d_6) spectrum of **12b**

Figure S36. HRMS spectrum of **12b**

Figure S37. ^{1}H-NMR (400 MHz, DMSO-d_6) spectrum of **13**

Figure S38. ^{13}C-NMR (400 MHz, DMSO-d_6) spectrum of **13**

Figure S39. HRMS spectrum of **13**

Figure S40. ^{1}H-NMR (400 MHz, DMSO-d_6) spectrum of **14**

Figure S41. ^{13}C-NMR (400 MHz, DMSO-d_6) spectrum of **14**

Figure S42. HRMS spectrum of **14**

Figure S43. ^{1}H-NMR (400 MHz, DMSO-d_6) spectrum of **15**

Figure S44. ^{13}C-NMR (400 MHz, DMSO-d_6) spectrum of **15**

Figure S45. HRMS spectrum of **15**

Figure S46. ^{1}H-NMR (400 MHz, DMSO-d_6) spectrum of **16**

Figure S47. ^{13}C-NMR (400 MHz, DMSO-d_6) spectrum of **16**

Figure S48. HRMS spectrum of **16**

Figure S49. ^{1}H-NMR (400 MHz, DMSO-d_6) spectrum of **17**

Figure S50. ^{13}C-NMR (400 MHz, DMSO-d_6) spectrum of **17**

Figure S51. HRMS spectrum of **17**

Figure S52. ^{1}H-NMR (400 MHz, DMSO-d_6) spectrum of **18**

Figure S53. ^{13}C-NMR (400 MHz, DMSO-d_6) spectrum of **18**

Figure S54. HRMS spectrum of **18**

Figure S55. ^{1}H-NMR (400 MHz, DMSO-d_6) spectrum of **19**

Figure S56. ^{13}C-NMR (400 MHz, DMSO-d_6) spectrum of **19**

Figure S57. HRMS spectrum of **19**

Figure S58. ^{1}H-NMR (400 MHz, DMSO-d_6) spectrum of **20**

Figure S59. ^{13}C-NMR (400 MHz, DMSO-d_6) spectrum of **20**

Figure S60. HRMS spectrum of **20**

Figure S61. ^{1}H-NMR (400 MHz, DMSO-d_6) spectrum of **21**

Figure S62. ^{13}C-NMR (400 MHz, DMSO-d_6) spectrum of **21**

Figure S63. HRMS spectrum of **21**

Figure S64. ^{1}H-NMR (400 MHz, DMSO-d_6) spectrum of **22**

Figure S65. ^{13}C-NMR (400 MHz, DMSO-d_6) spectrum of **22**

Figure S66. HRMS spectrum of **22**

Figure S67. ^{1}H-NMR (400 MHz, DMSO-d_6) spectrum of **23**

Figure S68. ^{13}C-NMR (400 MHz, DMSO-d_6) spectrum of **23**

Figure S69. HRMS spectrum of **23**

Figure S70. ^{1}H-NMR (400 MHz, DMSO-d_6) spectrum of **24**

Figure S71. ^{13}C-NMR (400 MHz, DMSO-d$_6$) spectrum of **24**

Figure S72. HRMS spectrum of **24**

Figure S73. ^{1}H-NMR (400 MHz, DMSO-d$_6$) spectrum of **25**

Figure S74. ^{13}C-NMR (400 MHz, DMSO-d$_6$) spectrum of **25**

Figure S75. HRMS spectrum of **25**

Figure S76. ^{1}H-NMR (400 MHz, DMSO-d$_6$) spectrum of **26**

Figure S77. ^{13}C-NMR (400 MHz, DMSO-d$_6$) spectrum of **26**

Figure S78. HRMS spectrum of **26**

Figure S79. ^{1}H-NMR (400 MHz, DMSO-d$_6$) spectrum of **27a**

Figure S80. ^{13}C-NMR (400 MHz, DMSO-d$_6$) spectrum of **27a**

Figure S81. HRMS spectrum of **27a**

Figure S82. ^{1}H-NMR (400 MHz, DMSO-d$_6$) spectrum of **27b**

Figure S83. ^{13}C-NMR (400 MHz, DMSO-d$_6$) spectrum of **27b**

Figure S84. HRMS spectrum of **27b**

Figure S85. ^{1}H-NMR (400 MHz, DMSO-d$_6$) spectrum of **28**

Figure S86. ^{13}C-NMR (400 MHz, DMSO-d$_6$) spectrum of **28**

Figure S87. HRMS spectrum of **28**

Figure S88. ^{1}H-NMR (400 MHz, DMSO-d$_6$) spectrum of **29**

Figure S89. ^{13}C-NMR (400 MHz, DMSO-d$_6$) spectrum of **29**

Figure S90. HRMS spectrum of **29**

Figure S91. ^{1}H-NMR (400 MHz, DMSO-d$_6$) spectrum of **30**

Figure S92. ^{13}C-NMR (400 MHz, DMSO-d$_6$) spectrum of **30**

Figure S93. HRMS spectrum of **30**

Figure S94. ^{1}H-NMR (400 MHz, DMSO-d$_6$) spectrum of **31**

Figure S95. ^{13}C-NMR (400 MHz, DMSO-d$_6$) spectrum of **31**

Figure S96. HRMS spectrum of **31**

Figure S97. ^{1}H-NMR (400 MHz, DMSO-d$_6$) spectrum of **32**

Figure S98. ^{13}C-NMR (400 MHz, DMSO-d$_6$) spectrum of **32**

Figure S99. HRMS spectrum of **32**

Figure S100. ^{1}H-NMR (400 MHz, DMSO-d_6) spectrum of **33**

Figure S101. ^{13}C-NMR (400 MHz, DMSO-d_6) spectrum of **33**

Figure S102. HRMS spectrum of **33**

Figure S103. ^{1}H-NMR (400 MHz, DMSO-d_6) spectrum of **34**

Figure S104. ^{13}C-NMR (400 MHz, DMSO-d_6) spectrum of **34**

Figure S105. HRMS spectrum of **34**

Figure S106. ^{1}H-NMR (400 MHz, DMSO-d_6) spectrum of **35**

Figure S107. ^{13}C-NMR (400 MHz, DMSO-d_6) spectrum of **35**

Figure S108. HRMS spectrum of **35**

Figure S109. ^{1}H-NMR (400 MHz, DMSO-d_6) spectrum of **36**

Figure S110. ^{13}C-NMR (400 MHz, DMSO-d_6) spectrum of **36**

Figure S111. HRMS spectrum of **36**

Figure S112. ^{1}H-NMR (400 MHz, DMSO-d_6) spectrum of **37**

Figure S113. ^{13}C-NMR (400 MHz, DMSO-d_6) spectrum of **37**

Figure S114. HRMS spectrum of **37**

Figure S115. ^{1}H-NMR (400 MHz, DMSO-d_6) spectrum of **38**

Figure S116. ^{13}C-NMR (400 MHz, DMSO-d_6) spectrum of **38**

Figure S117. HRMS spectrum of **38**

Figure S118. ^{1}H-NMR (400 MHz, DMSO-d_6) spectrum of **39**

Figure S119. ^{13}C-NMR (400 MHz, DMSO-d_6) spectrum of **39**

Figure S120. HRMS spectrum of **39**

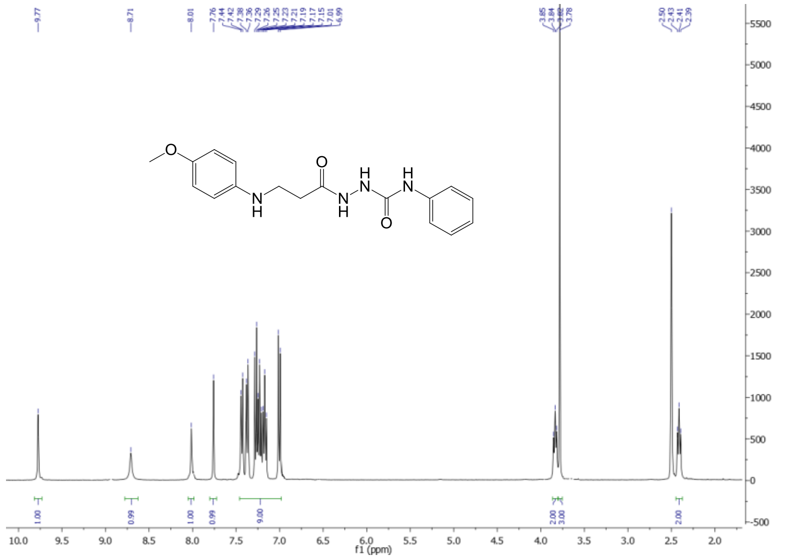

Figure S1. ^{1}H-NMR (400 MHz, DMSO-*d*$_6$) spectrum of **2**

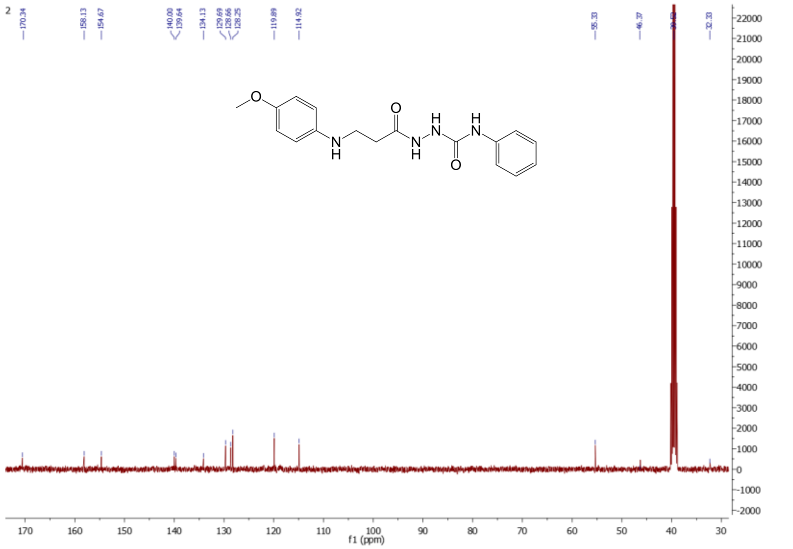

Figure S2. ^{13}C-NMR (400 MHz, DMSO-*d*$_6$) spectrum of **2**

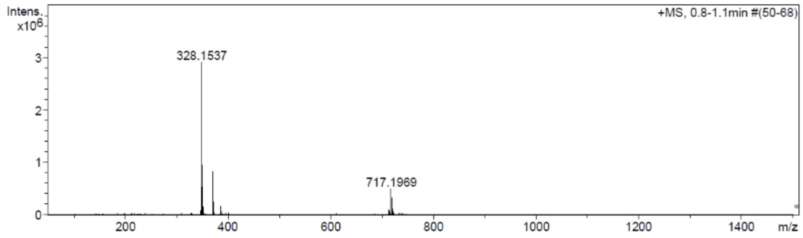

Meas. m/z	#	Formula	Score	m/z	err [ppm]	Mean err [ppm]	mSigma	rdb	e⁻ Conf	N-Rule
328.1537	1	C 17 H 20 N 4 O 3	100.00	328.1537	-2.1	-1.6	11.2	9.5	even	ok

Figure S3. HRMS spectrum of **2**

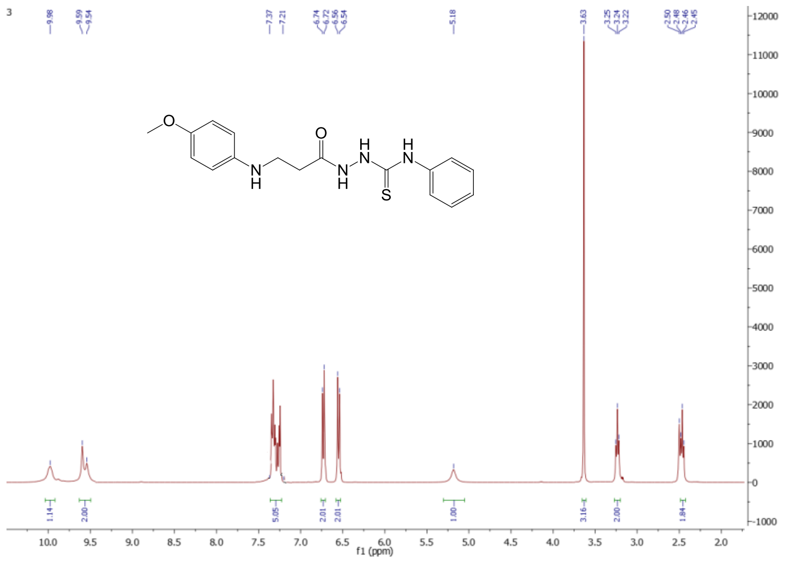

Figure S4. ^{1}H-NMR (400 MHz, DMSO-d_6) spectrum of **3**

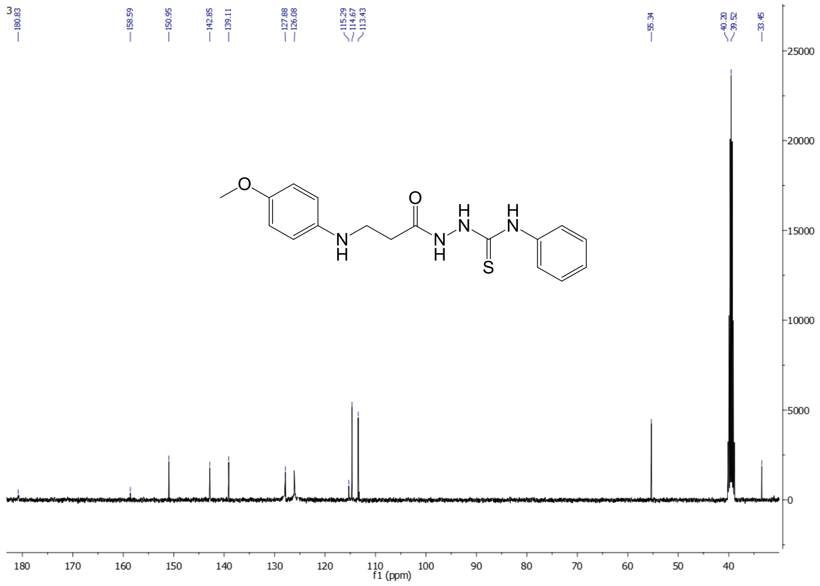

Figure S5. ^{13}C-NMR (400 MHz, DMSO-d_6) spectrum of **3**

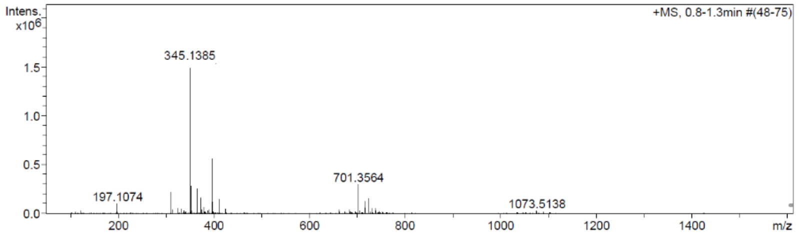

Meas. m/z	#	Formula	Score	m/z	err [ppm]	Mean err [ppm]	mSigma	rdb	e⁻ Conf	N-Rule
345.1385	1	C 17 H 21 N 4 O 2 S	100.00	345.1385	-2.7	-2.5	3.8	11.0	odd	ok

Figure S6. HRMS spectrum of **3**

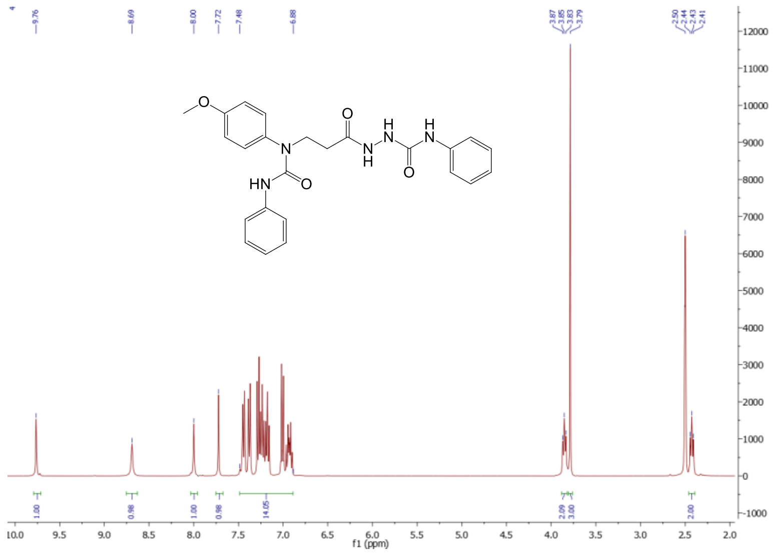

Figure S7. ^{1}H-NMR (400 MHz, DMSO-d_6) spectrum of **4**

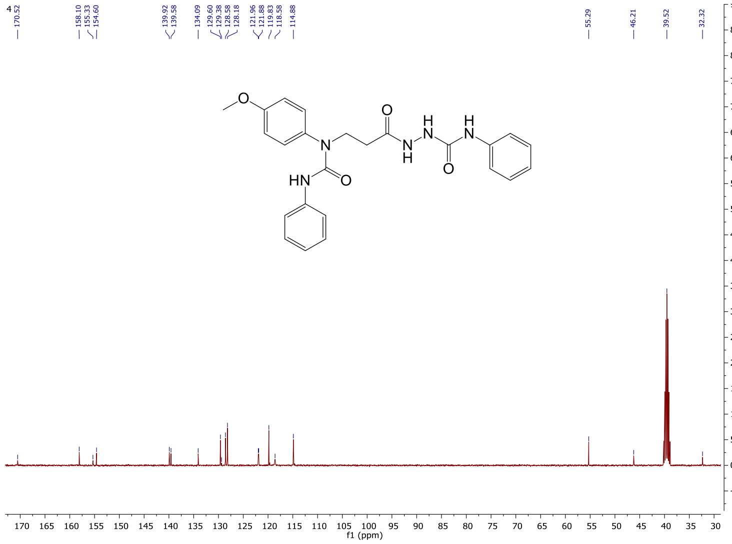

Figure S8. ^{13}C-NMR (400 MHz, DMSO-d_6) spectrum of **4**

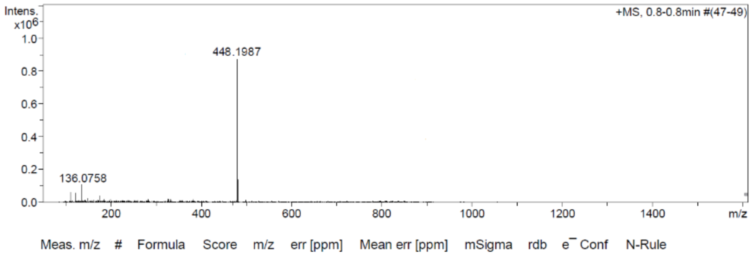

Figure S9. HRMS spectrum of **4**

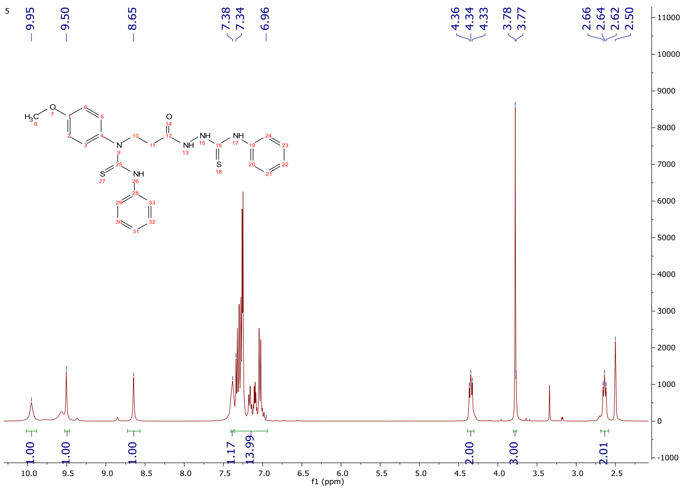

Figure S10. ^{1}H-NMR (400 MHz, DMSO-*d*$_6$) spectrum of **5**

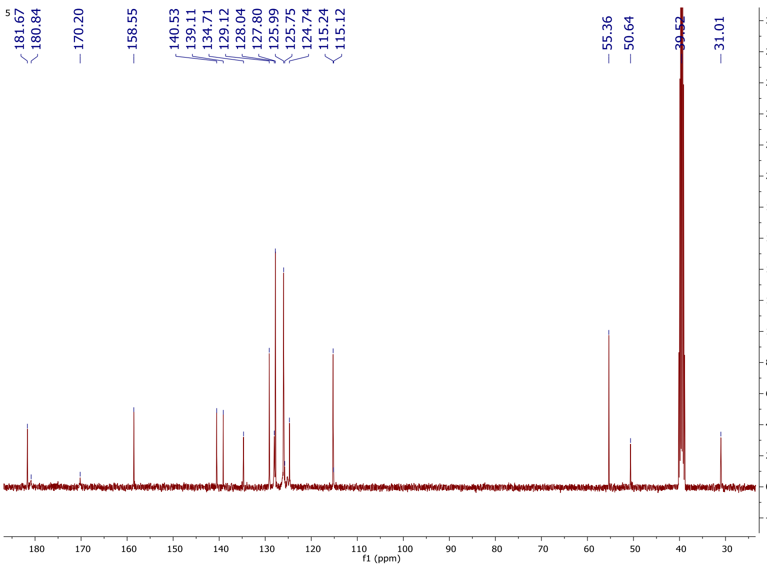

Figure S11. ^{13}C-NMR (400 MHz, DMSO-*d*$_6$) spectrum of **5**

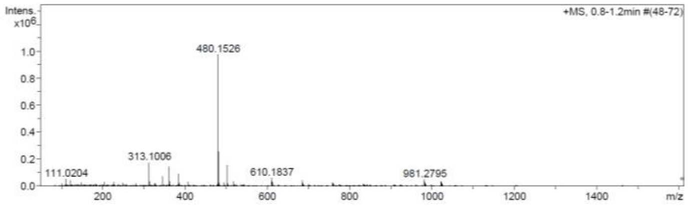

Meas. m/z	#	Formula	Score	m/z	err [ppm]	Mean err [ppm]	mSigma	rdb	e⁻ Conf	N-Rule
480.1526	1	C 17 H 18 N 15 O S	88.18	480.1534	1.6	1.5	11.0	16.5	even	ok
	2	C 18 H 24 N 8 O 6 S	81.08	480.1534	1.6	1.6	15.1	11.0	odd	ok
	3	C 19 H 30 N O 11 S	72.67	480.1534	1.6	1.6	20.3	5.5	even	ok
	4	C 25 H 24 N 2 O 8	100.00	480.1527	0.2	1.9	20.6	15.0	odd	ok
	5	C 23 H 22 N 5 O 7	54.17	480.1514	-2.6	-0.9	21.3	15.5	even	ok
	6	C 22 H 24 N 8 O S 2	37.22	480.1509	-3.6	-3.6	24.0	15.0	odd	ok
	7	C 17 H 28 N 4 O 10 S	72.12	480.1521	-1.2	-1.2	25.6	6.0	odd	ok
	8	C 23 H 30 N O 6 S 2	35.89	480.1509	-3.6	-3.6	25.8	9.5	even	ok
	9	C 22 H 16 N 12 O 2	45.66	480.1514	-2.6	-1.3	28.6	21.0	odd	ok
	10	C 24 H 18 N 9 O 3	82.20	480.1527	0.2	1.5	29.3	20.5	even	ok
	11	C 24 H 26 N 5 O 2 S 2	70.86	480.1522	-0.8	-0.8	29.9	14.5	even	ok
	12	C 26 H 20 N 6 O 4	38.40	480.1541	3.0	4.4	31.4	20.0	odd	ok
	13	C 26 H 28 N 2 O 3 S 2	46.33	480.1536	2.0	2.0	35.2	14.0	odd	ok
	14	C 32 H 22 N 3 S	38.80	480.1529	0.6	1.9	53.5	23.5	even	ok

Figure S12. HRMS spectrum of **5**

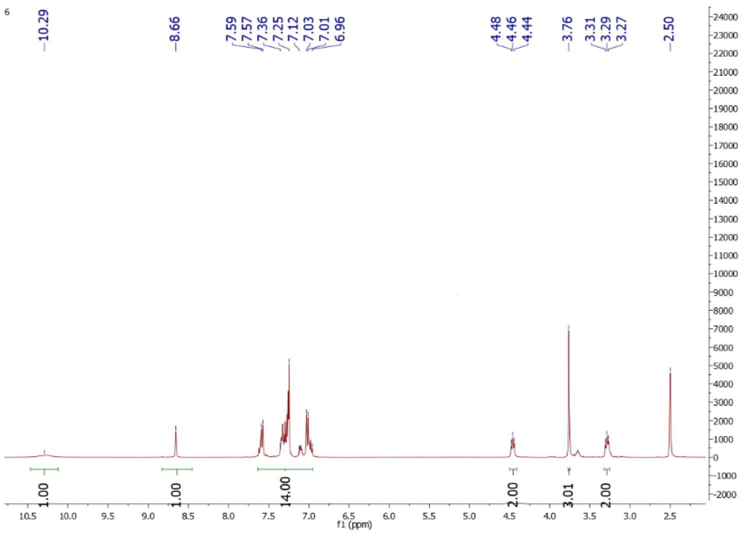

Figure S13. ^{1}H-NMR (400 MHz, DMSO-*d*6) spectrum of **6**

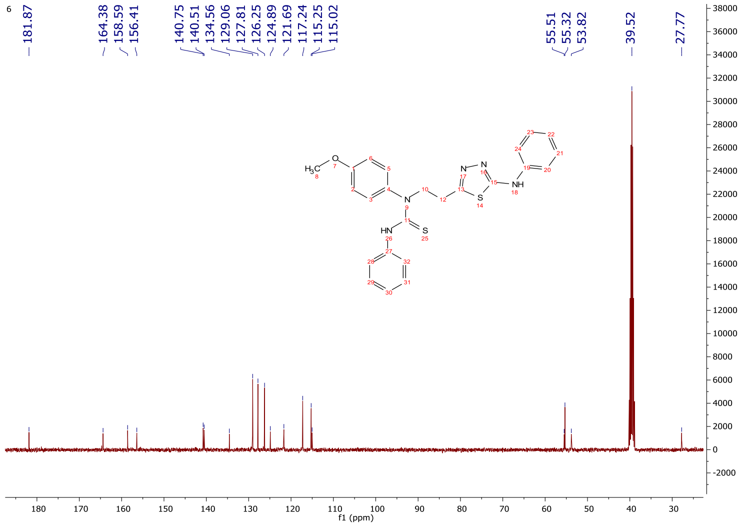

Figure S14. ^{13}C-NMR (400 MHz, DMSO-*d*6) spectrum of **6**

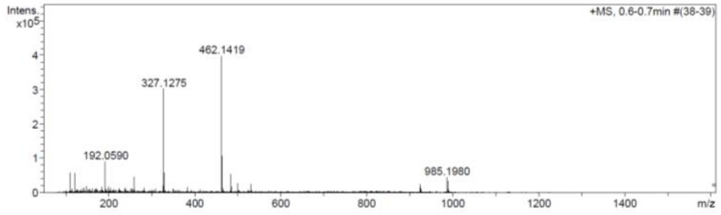

Meas. m/z	#	Formula	Score	m/z	err [ppm]	Mean err [ppm]	mSigma	rdb	e⁻ Conf	N-Rule
327.1275	1	C 15 H 17 N 7 S	52.40	327.1261	-4.5	-4.4	9.1	11.0	odd	ok
	2	C 17 H 19 N 4 O S	100.00	327.1274	-0.3	-0.3	12.8	10.5	even	ok
	3	C 16 H 23 O 5 S	48.67	327.1261	-4.4	-4.4	13.1	5.5	even	ok
	4	C 19 H 21 N O 2 S	50.07	327.1288	3.8	3.8	18.7	10.0	odd	ok
	5	C 11 H 17 N 7 O 5	43.12	327.1286	3.2	4.2	30.1	7.0	odd	ok
	6	C 12 H 21 N 7 S 2	24.09	327.1294	5.9	5.8	30.5	6.0	odd	ok
	7	C 12 H 23 O 10	39.03	327.1286	3.2	4.3	34.0	1.5	even	ok
	8	C 13 H 27 O 5 S 2	20.30	327.1294	5.9	5.9	37.0	0.5	even	ok
	9	C 11 H 25 N 3 O 4 S 2	45.17	327.1281	1.8	1.8	38.2	1.0	odd	ok
462.1419	1	C 25 H 22 N 2 O 7	100.00	462.1422	0.5	2.0	18.4	16.0	odd	ok
	[illegible]	[illegible]	[illegible]	[illegible]	[illegible]	[illegible]	[illegible]	[illegible]	[illegible]	[illegible]

Figure S15. HRMS spectrum of **6**

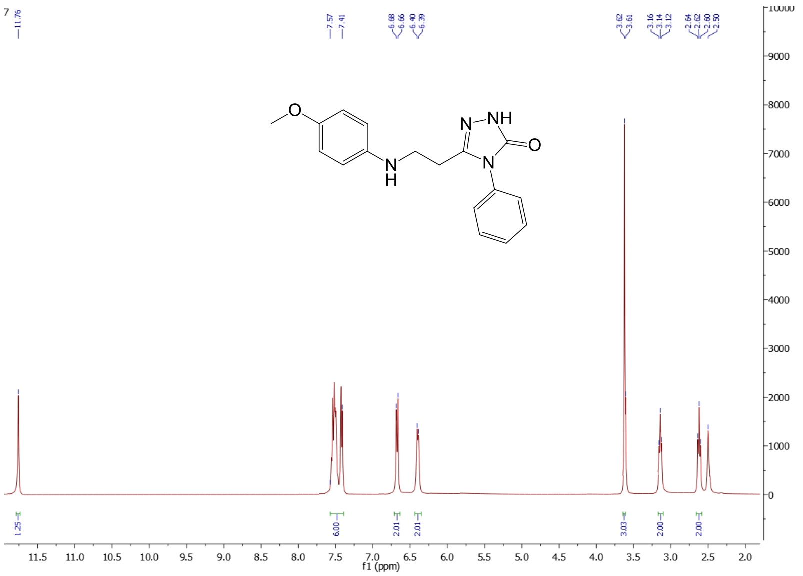

Figure S16. ^{1}H-NMR (400 MHz, DMSO-d_6) spectrum of **7**

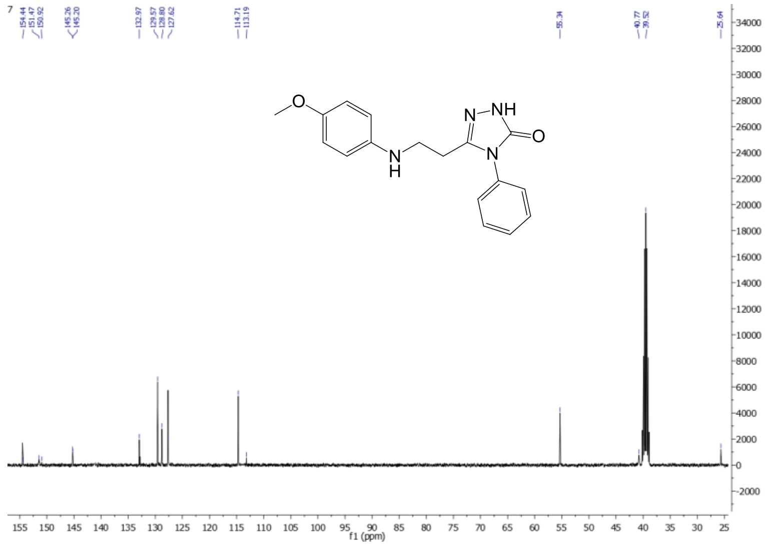

Figure S17. ^{13}C-NMR (400 MHz, DMSO-d_6) spectrum of **7**

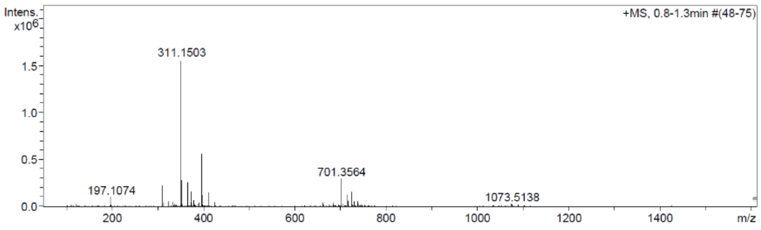

Meas. m/z	#	Formula	Score	m/z	err [ppm]	Mean err [ppm]	mSigma	rdb	e⁻ Conf	N-Rule
311.1498	1	C 15 H 17 N 7 O	87.43	311.1489	-2.7	-2.5	3.8	11.0	odd	ok
	2	C 17 H 19 N 4 O 2	100.00	311.1503	1.6	1.9	6.7	10.5	even	ok
	3	C 16 H 23 O 6	80.99	311.1489	-2.7	-2.4	8.3	5.5	even	ok
	4	C 19 H 21 N O 3	39.65	311.1516	5.9	6.2	13.0	10.0	odd	ok

Figure S18. HRMS spectrum of **7**

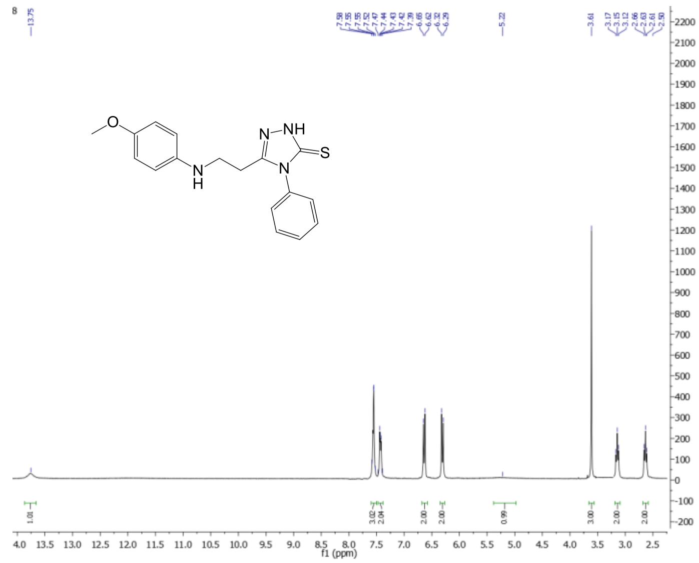

Figure S19. ^{1}H-NMR (400 MHz, DMSO-d_6) spectrum of **8**

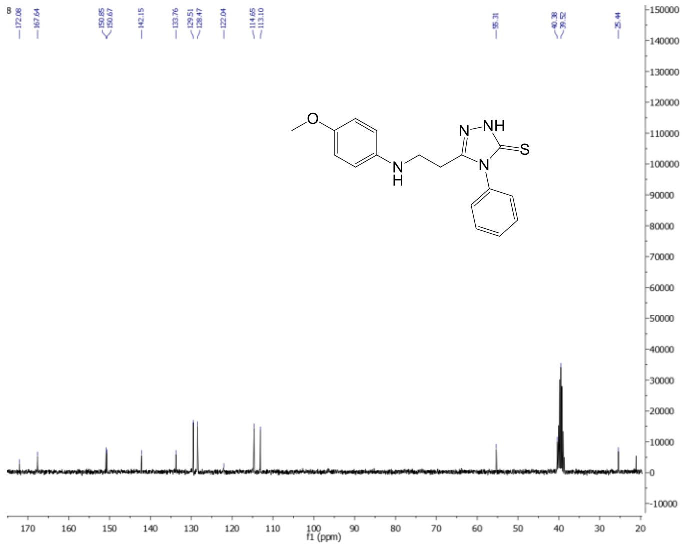

Figure S20. ^{13}C-NMR (400 MHz, DMSO-d_6) spectrum of **8**

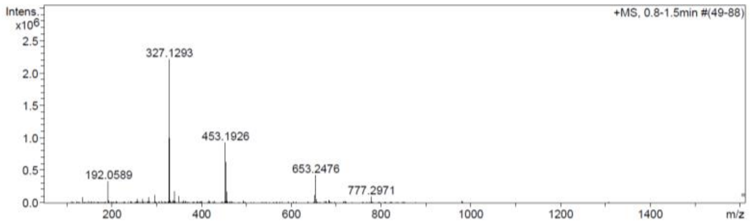

Meas. m/z	#	Formula	Score	m/z	err [ppm]	Mean err [ppm]	mSigma	rdb	e⁻ Conf	N-Rule
192.0589	1	C 3 H 8 N 6 O 4	100.00	192.0602	6.7	6.9	23.4	3.0	odd	ok
	2	C 14 H 8 O	50.72	192.0570	-9.9	-9.6	33.7	11.0	odd	ok
327.1293	1	C 14 H 15 N 8 O 2	64.69	327.1312	6.0	7.7	157.4	11.5	even	ok
	2	C 12 H 13 N 11 O	100.00	327.1299	1.9	3.5	163.3	12.0	odd	ok
	3	C 15 H 21 N O 7	34.19	327.1313	6.1	7.8	166.5	6.0	odd	ok

Figure S21. HRMS spectrum of **8**

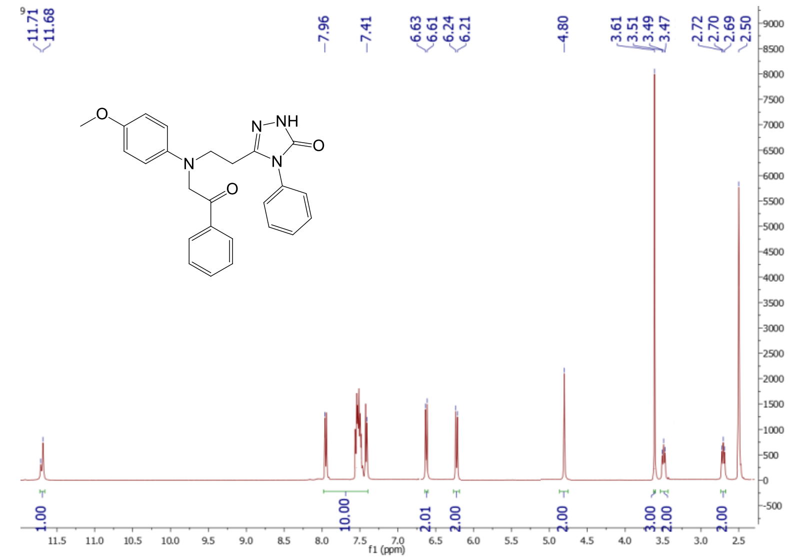

Figure S22. ^{1}H-NMR (400 MHz, DMSO-d_6) spectrum of **9**

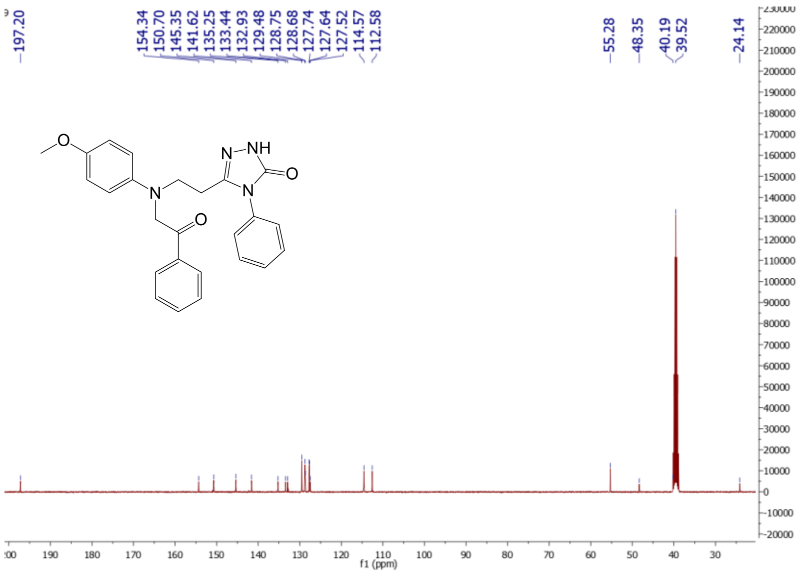

Figure S23. ^{13}C-NMR (400 MHz, DMSO-d_6) spectrum of **9**

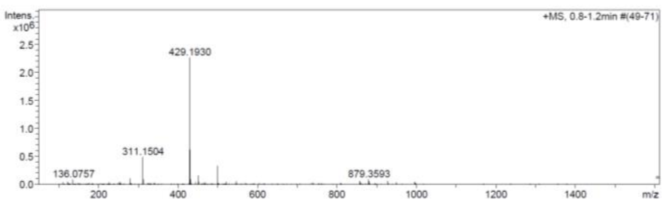

Meas. m/z	#	Formula	Score	m/z	err [ppm]	Mean err [ppm]	mSigma	rdb	e⁻ Conf	N-Rule
429.1930	1	C 25 H 25 N 4 O 3	86.87	429.1921	-2.1	-1.7	9.1	15.5	even	ok
	2	C 27 H 27 N O 4	100.00	429.1935	1.0	1.5	13.7	15.0	odd	ok
	3	C 28 H 23 N 5	35.29	429.1948	4.2	4.6	25.4	20.0	odd	ok

Figure S24. HRMS spectrum of **9**

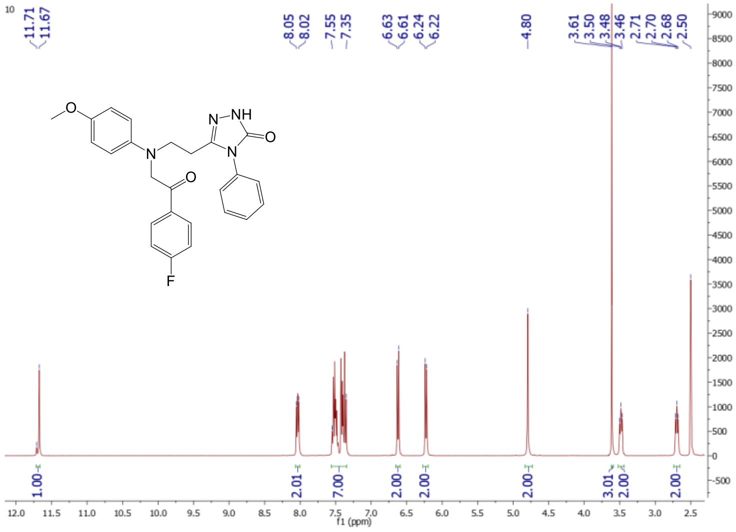

Figure S25. ^{1}H-NMR (400 MHz, DMSO-*d*6) spectrum of **10**

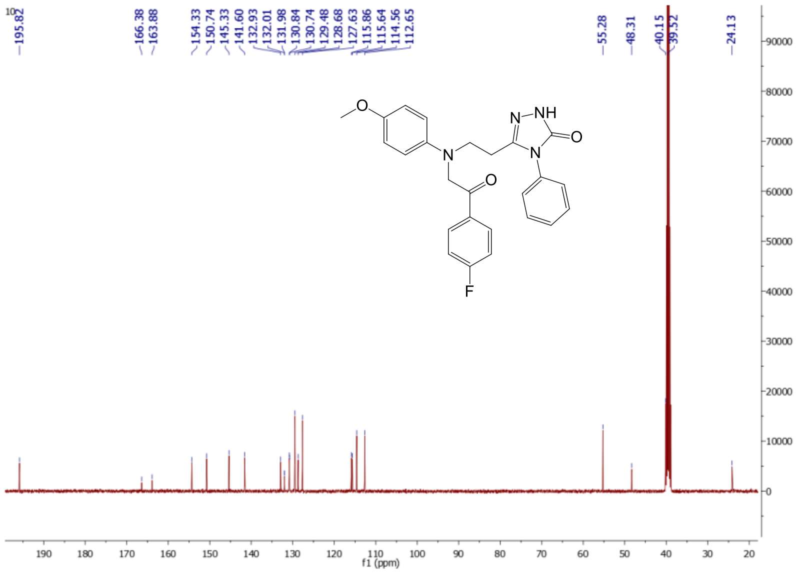

Figure S26. ^{13}C-NMR (400 MHz, DMSO-*d*6) spectrum of **10**

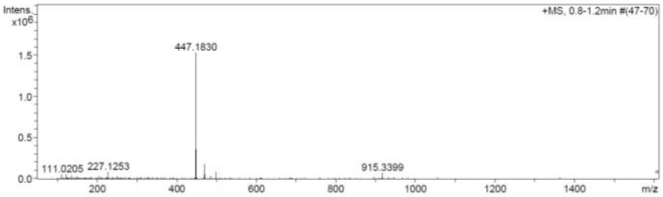

Meas. m/z	#	Formula	Score	m/z	err [ppm]	Mean err [ppm]	mSigma	rdb	e⁻ Conf	N-Rule
447.1830	1	C 17 H 23 N 10 O 5	51.12	447.1847	3.9	4.2	5.2	11.5	even	ok
	2	C 16 H 17 N 17	50.60	447.1847	3.9	4.1	6.0	17.0	odd	ok
	3	C 18 H 29 N 3 O 10	45.97	447.1847	3.9	4.1	10.9	6.0	odd	ok
	4	C 15 H 21 N 13 O 4	100.00	447.1834	0.9	1.1	11.7	12.0	odd	ok
	5	C 24 H 28 F O 7	45.34	447.1814	-3.6	-3.3	16.3	10.5	even	ok
	6	C 16 H 27 N 6 O 9	84.22	447.1834	0.9	1.1	20.1	6.5	even	ok
	7	C 23 H 22 F N 7 O 2	36.46	447.1814	-3.7	-3.4	26.1	16.0	odd	ok
	8	C 15 H 31 N 2 O 13	56.79	447.1821	-2.1	-1.9	26.2	1.5	even	ok
	9	C 25 H 24 F N 4 O 3	66.53	447.1827	-0.7	-0.4	32.7	15.5	even	ok

Figure S27. HRMS spectrum of **10**

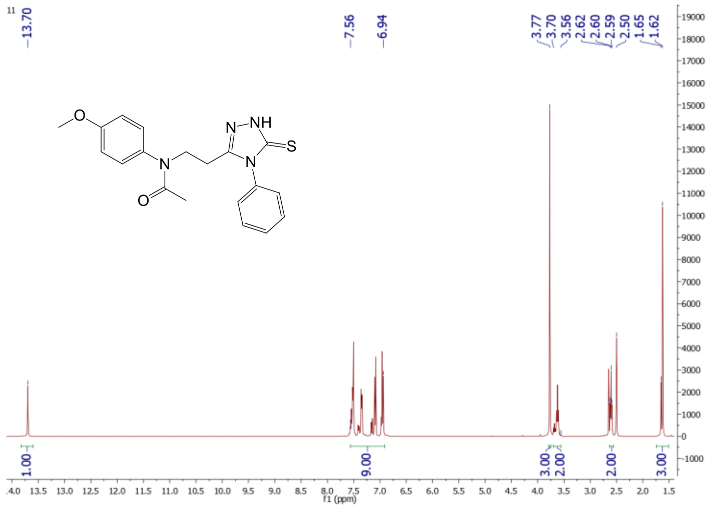

Figure S28. ^{1}H-NMR (400 MHz, DMSO-d_6) spectrum of **11**

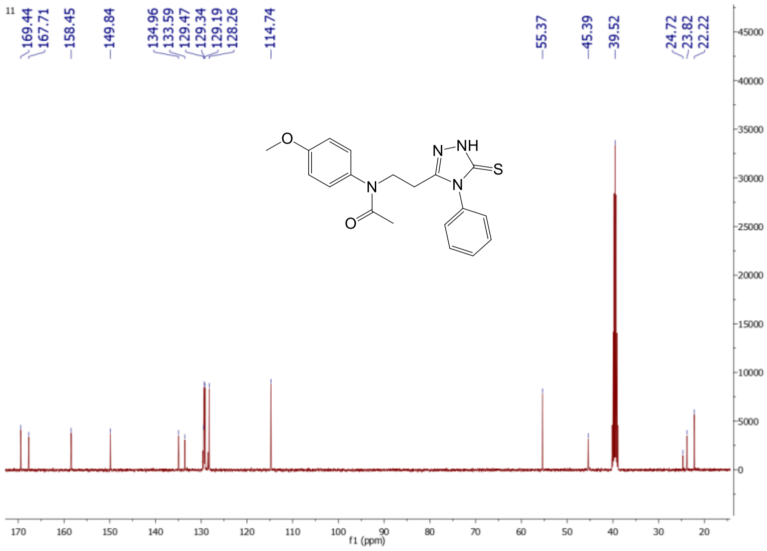

Figure S29. ^{13}C-NMR (400 MHz, DMSO-d_6) spectrum of **11**

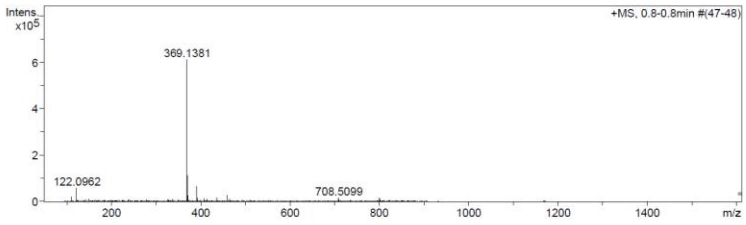

Meas. m/z	#	Formula	Score	m/z	err [ppm]	Mean err [ppm]	mSigma	rdb	e⁻ Conf	N-Rule
369.1381	1	C 13 H 19 N 7 O 6	75.35	369.1391	2.9	3.7	13.8	8.0	odd	ok
	2	C 12 H 13 N 14 O	72.27	369.1391	2.9	3.9	16.0	13.5	even	ok
	3	C 14 H 25 O 11	70.99	369.1391	2.9	3.8	16.6	2.5	even	ok
	4	C 11 H 17 N 10 O 5	100.00	369.1378	-0.7	0.0	19.9	8.5	even	ok
	5	C 18 H 25 O 6 S	53.25	369.1366	-3.9	-3.8	20.0	6.5	even	ok
	6	C 17 H 19 N 7 O S	51.41	369.1366	-3.9	-3.9	21.4	12.0	odd	ok
	7	C 12 H 23 N 3 O 10	93.11	369.1378	-0.7	0.1	23.3	3.0	odd	ok
	8	C 19 H 21 N 4 O 2 S	90.88	369.1380	-0.2	-0.2	27.6	11.5	even	ok

Figure S30. HRMS spectrum of **11**

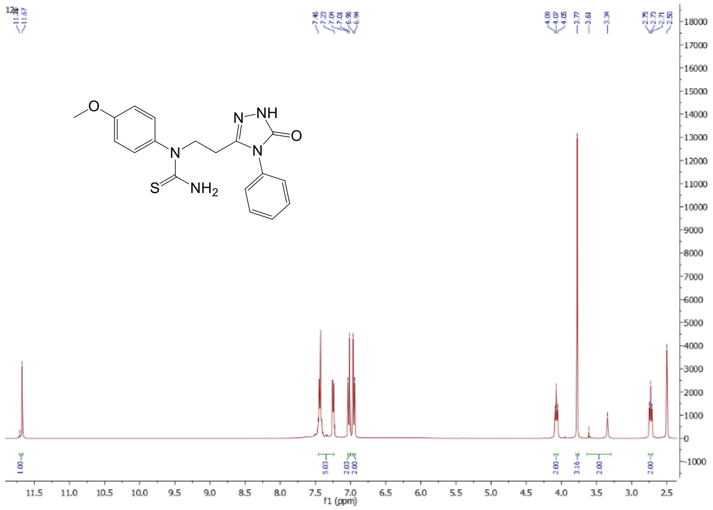

Figure S31. ^{1}H-NMR (400 MHz, DMSO-*d*6) spectrum of **12a**

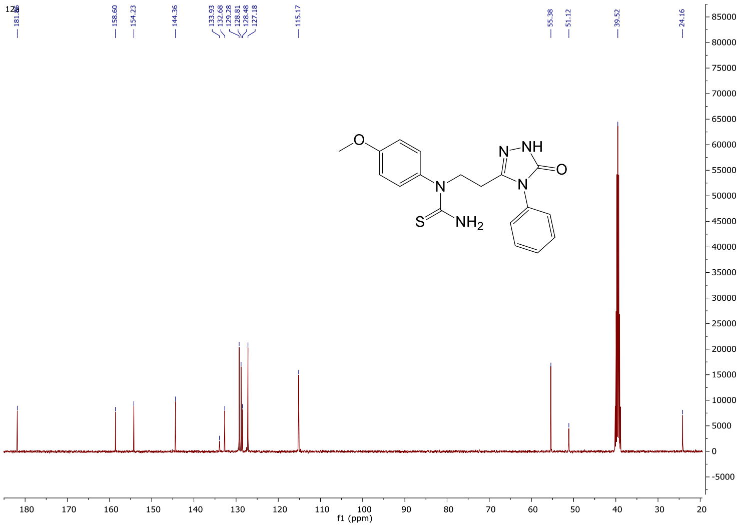

Figure S32. ^{13}C-NMR (400 MHz, DMSO-*d*6) spectrum of **12a**

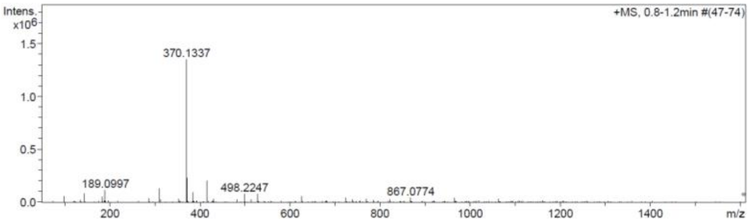

Meas. m/z	#	Formula	Score	m/z	err [ppm]	Mean err [ppm]	mSigma	rdb	e⁻ Conf	N-Rule
370.1337	1	C 14 H 20 N 5 O 7	46.35	370.1357	5.4	6.2	5.3	7.5	even	ok
	2	C 12 H 18 N 8 O 6	100.00	370.1344	1.8	2.5	9.8	8.0	odd	ok
	3	C 13 H 24 N O 11	94.36	370.1344	1.8	2.6	12.7	2.5	even	ok
	4	C 13 H 14 N 12 O 2	40.21	370.1357	5.4	6.3	13.2	13.0	odd	ok
	5	C 11 H 12 N 15 O	92.47	370.1344	1.8	2.1	14.0	13.5	even	ok
	6	C 10 H 16 N 11 O 5	87.80	370.1330	-1.9	-1.1	15.8	8.5	even	ok
	7	C 11 H 22 N 4 O 10	81.79	370.1330	-1.8	-1.1	19.3	3.0	odd	ok
	8	C 17 H 24 N O 6 S	36.67	370.1319	-5.0	-4.9	23.2	6.5	even	ok
	9	C 16 H 18 N 8 O S	35.14	370.1319	-5.0	-4.9	24.9	12.0	odd	ok
	10	C 18 H 20 N 5 O 2 S	68.23	370.1332	-1.4	-1.3	31.1	11.5	even	ok

Figure S33. HRMS spectrum of **12a**

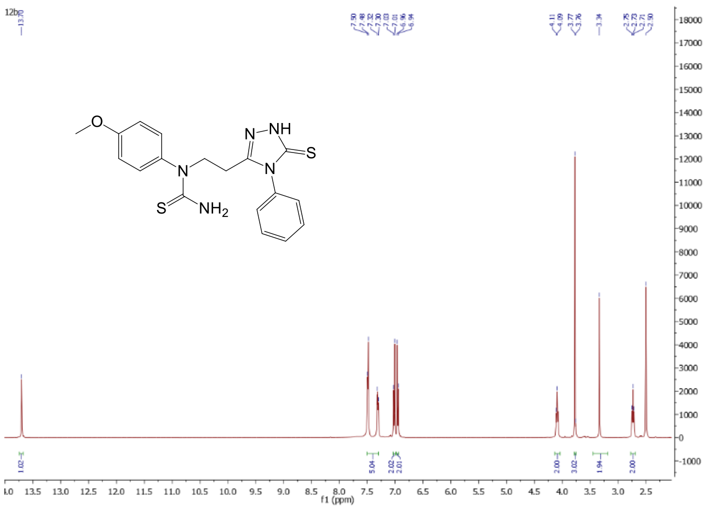

Figure S34. ^{1}H-NMR (400 MHz, DMSO-d_6) spectrum of **12b**

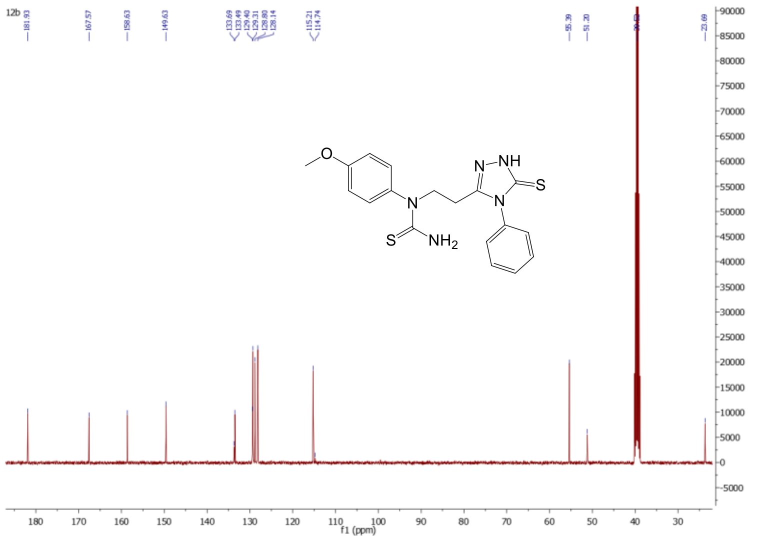

Figure S35. ^{13}C-NMR (400 MHz, DMSO-d_6) spectrum of **12b**

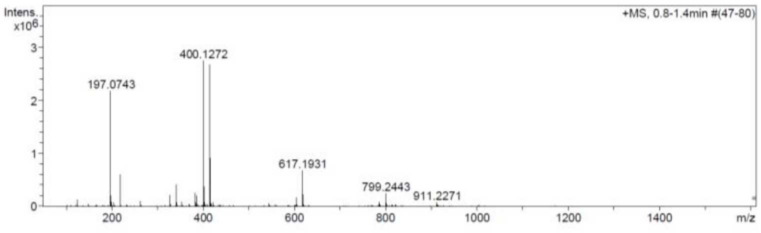

Meas. m/z	#	Formula	Score	m/z	err [ppm]	Mean err [ppm]	mSigma	rdb	e⁻ Conf	N-Rule
197.0743	1	C 7 H 11 N 5 S	54.92	197.0730	-6.8	-6.5	7.9	5.0	odd	ok
	2	C 9 H 13 N 2 O S	100.00	197.0743	0.1	0.3	12.1	4.5	even	ok
386.1100	1	C 16 H 18 N 8 S 2	75.60	386.1090	-2.6	-2.5	9.8	12.0	odd	ok
	2	C 14 H 8 N 15	41.03	386.1082	-4.9	-4.6	11.9	18.5	even	ok
	3	C 16 H 10 N 12 O	91.88	386.1095	-1.4	-1.1	12.6	18.0	odd	ok
	4	C 18 H 20 N 5 O S 2	100.00	386.1104	0.9	1.0	13.0	11.5	even	ok

Figure S36. HRMS spectrum of **12b**

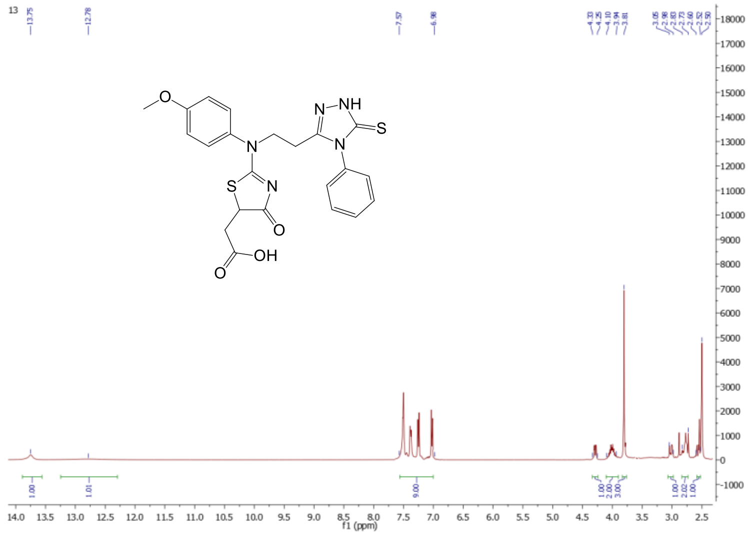

Figure S37. ^{1}H-NMR (400 MHz, DMSO-d_6) spectrum of **13**

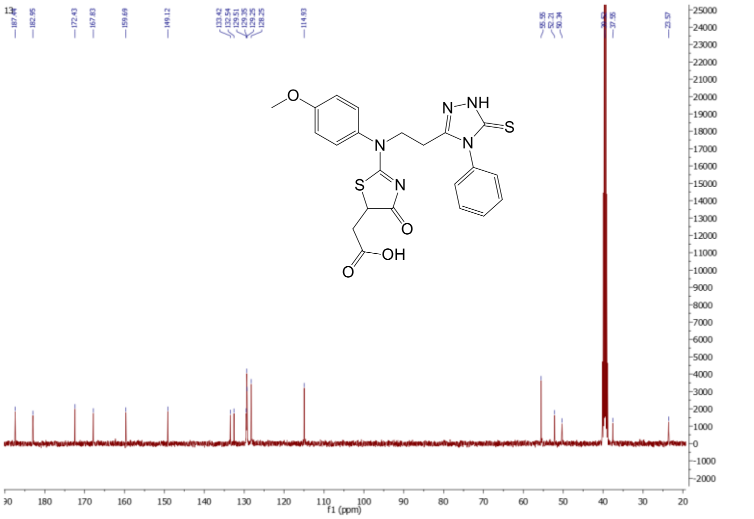

Figure S38. ^{13}C-NMR (400 MHz, DMSO-d_6) spectrum of **13**

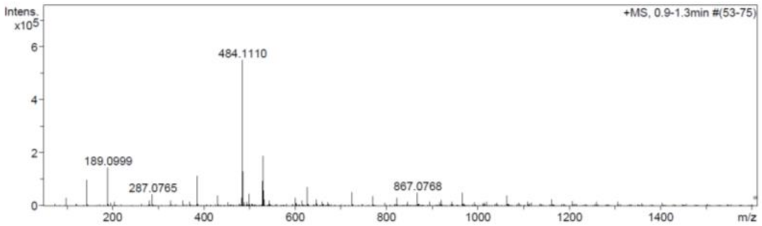

Meas. m/z	#	Formula	Score	m/z	err [ppm]	Mean err [ppm]	mSigma	rdb	e⁻ Conf	N-Rule
484.1110	1	C 15 H 14 N 15 O 3 S	95.43	484.1119	1.9	1.7	11.4	16.5	even	ok
	2	C 16 H 20 N 8 O 8 S	91.05	484.1119	1.9	1.8	13.7	11.0	odd	ok
	3	C 17 H 26 N O 13 S	84.32	484.1119	1.9	1.9	17.4	5.5	even	ok
	4	C 21 H 26 N O 8 S 2	49.27	484.1094	-3.3	-3.3	22.7	9.5	even	ok
	5	C 15 H 24 N 4 O 12 S	95.35	484.1106	-0.9	-0.9	23.0	6.0	odd	ok
	6	C 20 H 20 N 8 O 3 S 2	48.74	484.1094	-3.3	-3.4	23.0	15.0	odd	ok
	7	C 21 H 18 N 5 O 9	63.06	484.1099	-2.3	-0.6	25.0	15.5	even	ok
	8	C 23 H 20 N 2 O 10	100.00	484.1112	0.4	2.2	25.2	15.0	odd	ok
	9	C 22 H 22 N 5 O 4 S 2	91.27	484.1108	-0.5	-0.6	28.4	14.5	even	ok

Figure S39. HRMS spectrum of **13**

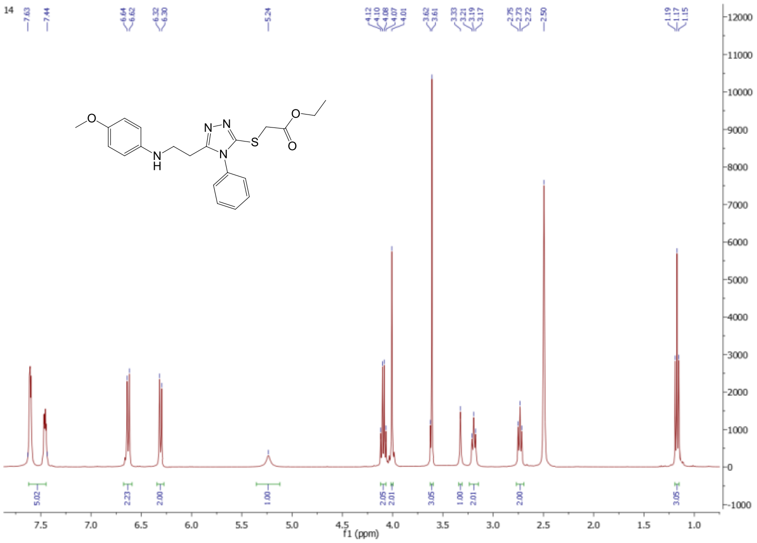

Figure S40. ^{1}H-NMR (400 MHz, DMSO-d_6) spectrum of **14**

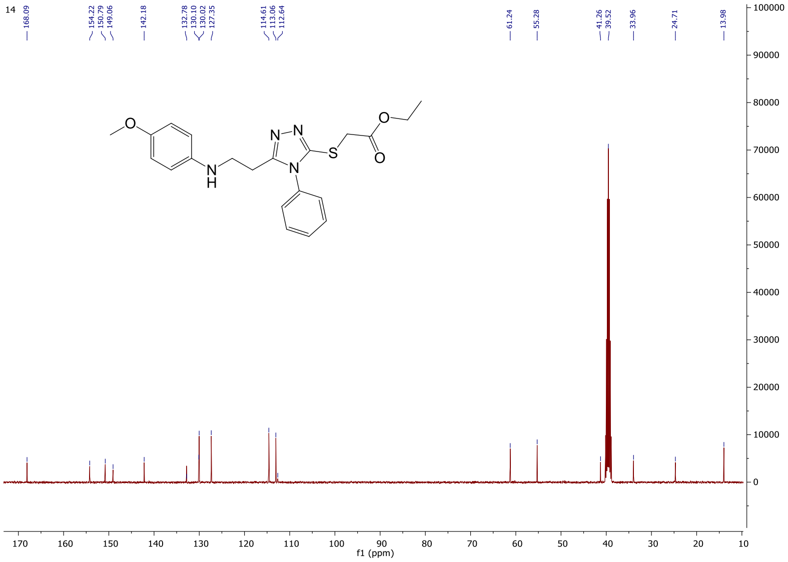

Figure S41. ^{13}C-NMR (400 MHz, DMSO-d_6) spectrum of **14**

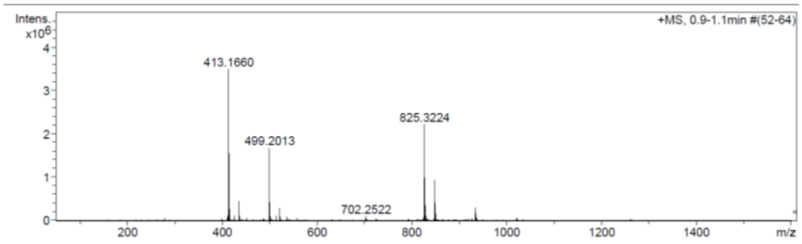

Meas. m/z	#	Formula	Score	m/z	err [ppm]	Mean err [ppm]	mSigma	rdb	e⁻ Conf	N-Rule
413.1660	1	C 29 H 21 N 2 O	100.00	413.1648	-2.7	-0.4	62.8	20.5	even	ok
	2	C 21 H 25 N 4 O 3 S	16.17	413.1642	-4.3	-3.3	97.3	11.5	even	ok
	[illegible]	[illegible]	[illegible]	[illegible]	[illegible]	[illegible]	[illegible]	[illegible]	[illegible]	[illegible]

Figure S42. HRMS spectrum of **14**

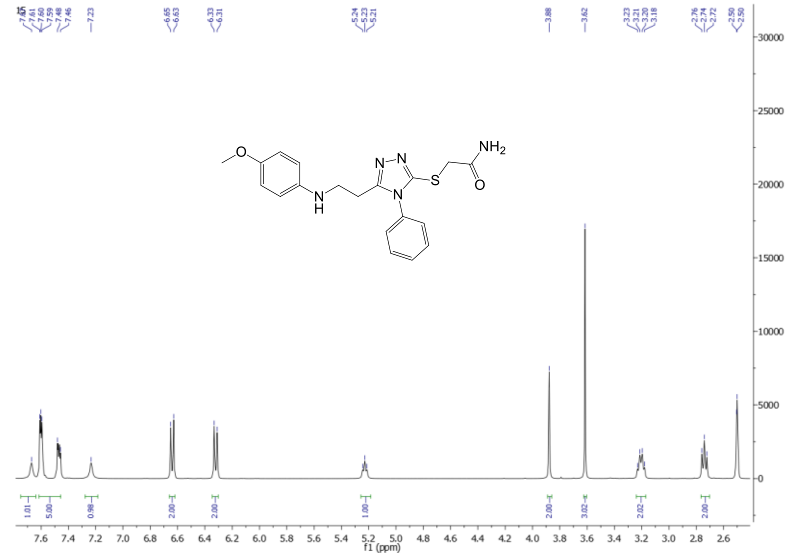

Figure S43. ^{1}H-NMR (400 MHz, DMSO-d_6) spectrum of **15**

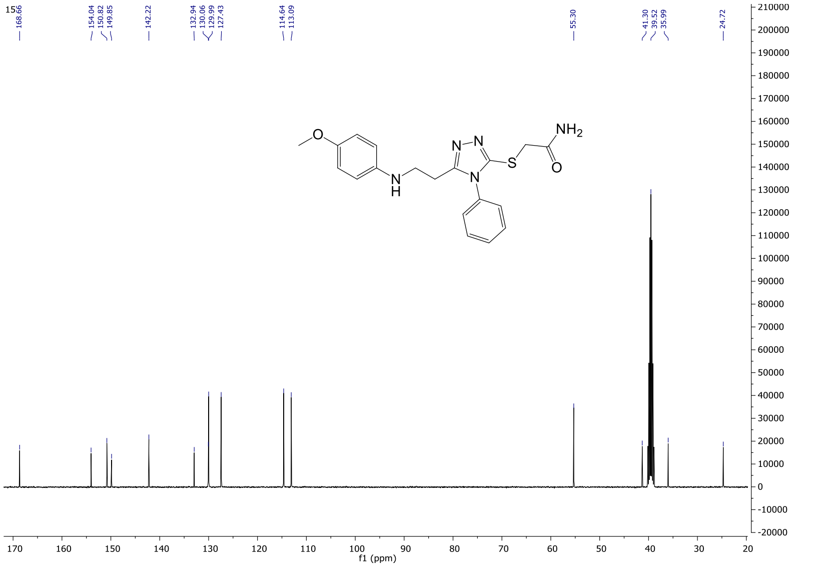

Figure S44. ^{13}C-NMR (400 MHz, DMSO-d_6) spectrum of **15**

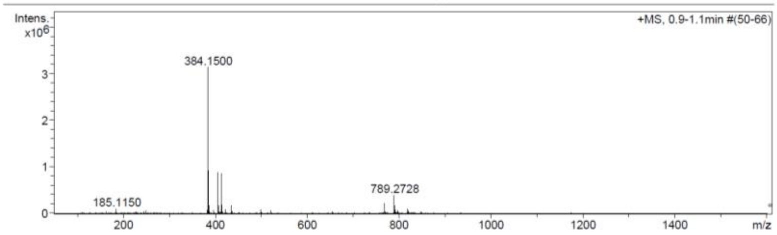

Meas. m/z	#	Formula	Score	m/z	err [ppm]	Mean err [ppm]	mSigma	rdb	e⁻ Conf	N-Rule
384.1500	1	C 27 H 18 N 3	100.00	384.1495	-1.2	0.4	7.6	20.5	even	ok

Figure S45. HRMS spectrum of **15**

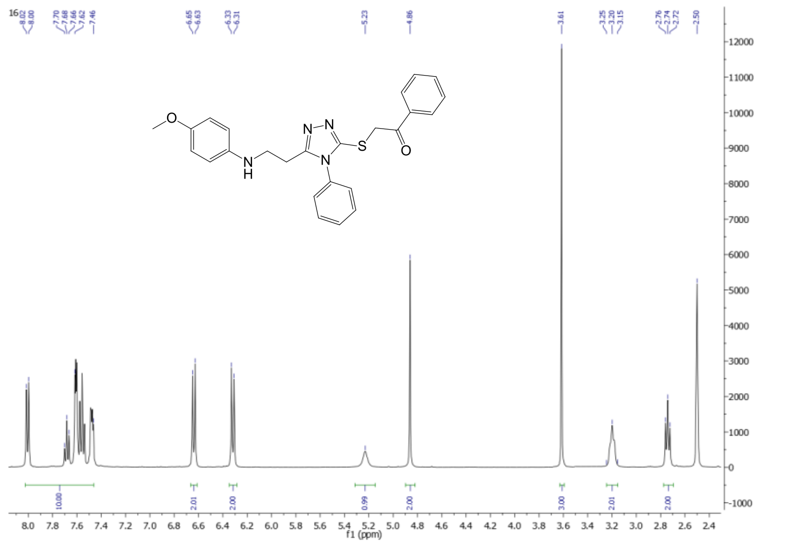

Figure S46. ^{1}H-NMR (400 MHz, DMSO-*d*$_6$) spectrum of **16**

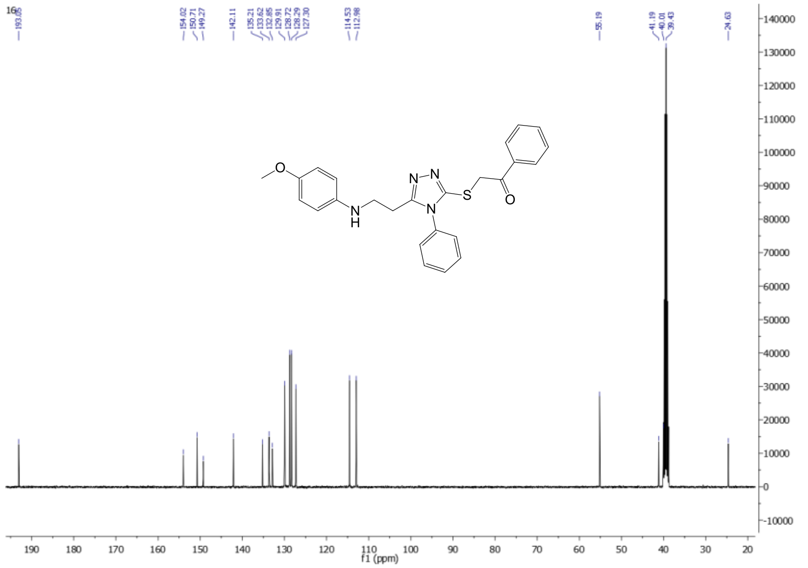

Figure S47. ^{13}C-NMR (400 MHz, DMSO-*d*$_6$) spectrum of **16**

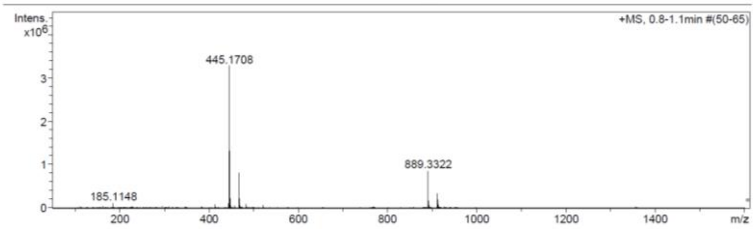

Meas. m/z	#	Formula	Score	m/z	err [ppm]	Mean err [ppm]	mSigma	rdb	e⁻ Conf	N-Rule
445.1708	1	C 33 H 21 N 2	100.00	445.1699	-1.9	-0.3	18.4	24.5	even	ok
889.3322	1	C 45 H 53 N 4 O 13 S	100.00	889.3324	0.3	0.9	6.8	21.5	even	ok
	2	C 42 H 45 N 14 O 7 S	63.67	889.3311	-1.2	-0.7	8.8	27.5	even	ok

Figure S48. HRMS spectrum of **16**

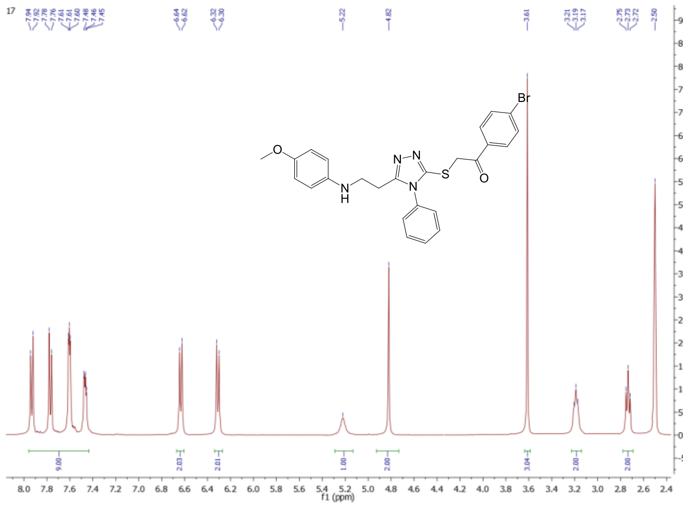

Figure S49 . ^{1}H-NMR (400 MHz, DMSO-*d*6) spectrum of **17**

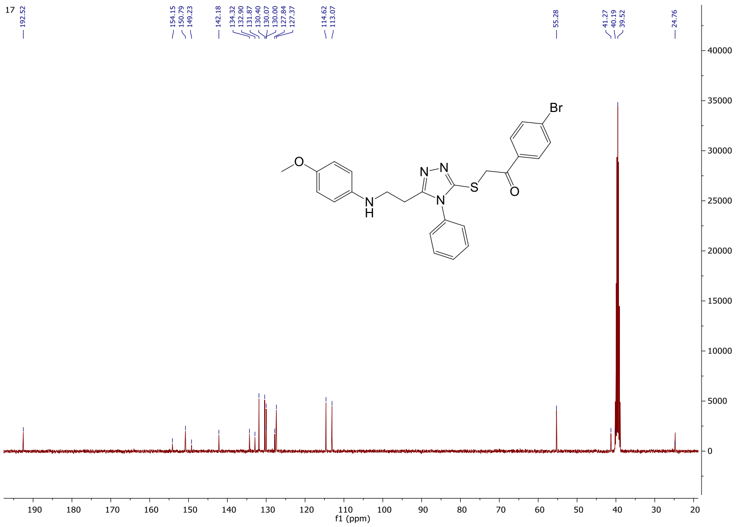

Figure S50. ^{13}C-NMR (400 MHz, DMSO-*d*6) spectrum of **17**

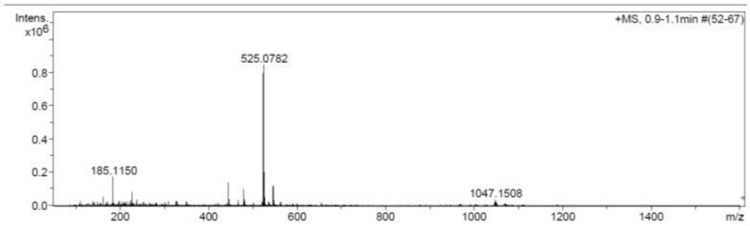

Meas. m/z	#	Formula	Score	m/z	err [ppm]	Mean err [ppm]	mSigma	rdb	e⁻ Conf	N-Rule
523.0799	1	C 24 H 28 Br O 6 S	61.81	523.0784	-2.8	-3.0	16.4	10.5	even	ok
	2	C 20 H 28 Br O 11	72.93	523.0810	2.0	2.3	21.1	6.5	even	ok
	3	C 18 H 24 Br N 10 S 2	87.55	523.0805	1.1	0.8	24.4	11.5	even	ok
	4	C 18 H 11 N 12 O 8	39.78	523.0817	3.5	3.8	25.4	19.5	even	ok
	5	C 25 H 24 Br N 4 O 2 S	100.00	523.0798	-0.2	-0.4	27.2	15.5	even	ok
	6	C 17 H 20 Br N 10 O 5	89.81	523.0796	-0.6	-0.3	28.3	12.5	even	ok
	7	C 18 H 16 Br N 14 O	61.11	523.0809	2.0	2.2	29.1	17.5	even	ok
	8	C 17 H 28 Br N 6 O 4 S 2	68.60	523.0791	-1.5	-1.7	30.0	6.5	even	ok
	9	C 21 H 32 Br O 6 S 2	32.98	523.0818	3.6	3.4	30.6	5.5	even	ok
	10	C 16 H 24 Br N 6 O 9	34.19	523.0783	-3.1	-2.9	36.6	7.5	even	ok
	11	C 29 H 11 N 6 O 5	24.48	523.0785	-2.6	-2.3	54.4	27.5	even	ok
	12	C 30 H 7 N 10 O	34.79	523.0799	-0.1	0.3	64.6	32.5	even	ok

Figure S51. HRMS spectrum of **17**

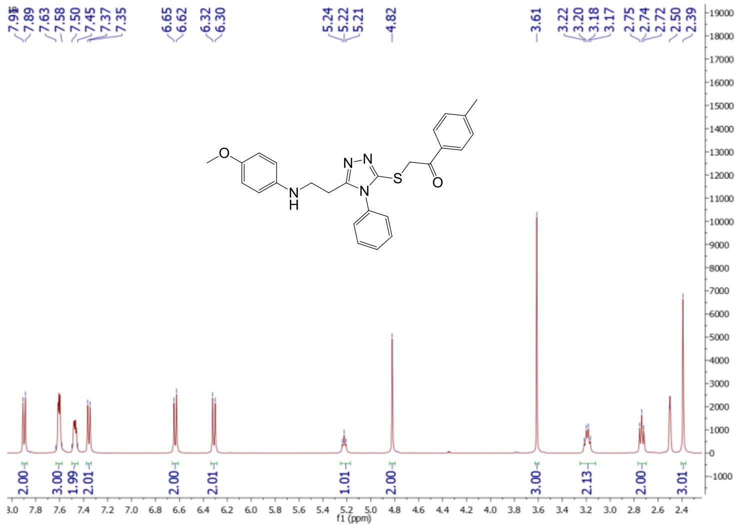

Figure S52. ^{1}H-NMR (400 MHz, DMSO-d_6) spectrum of **18**

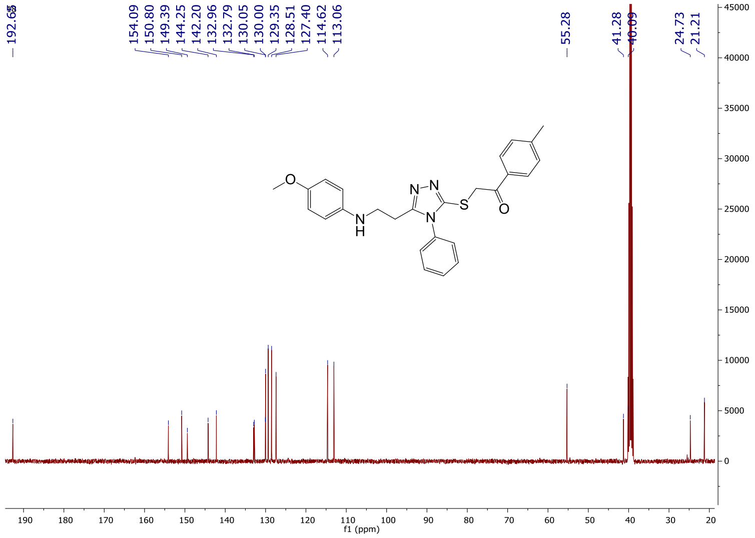

Figure S53. ^{13}C-NMR (400 MHz, DMSO-d_6) spectrum of **18**

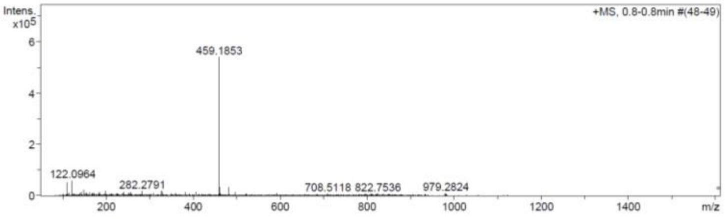

Meas. m/z	#	Formula	Score	m/z	err [ppm]	Mean err [ppm]	mSigma	rdb	e⁻ Conf	N-Rule
459.1853	1	C 21 H 31 O 11	99.63	459.1861	1.6	2.7	16.7	6.5	even	ok
	2	C 19 H 19 N 14 O	100.00	459.1861	1.6	2.4	16.8	17.5	even	ok
	3	C 20 H 25 N 7 O 6	98.87	459.1861	1.6	2.4	17.2	12.0	odd	ok
	4	C 24 H 25 N 7 O S	49.49	459.1836	-3.8	-4.1	19.7	16.0	odd	ok
	5	C 25 H 31 O 6 S	48.96	459.1836	-3.8	-4.0	20.4	10.5	even	ok
	6	C 18 H 23 N 10 O 5	91.63	459.1847	-1.3	-0.5	23.7	12.5	even	ok
	7	C 19 H 29 N 3 O 10	90.80	459.1847	-1.3	-0.5	24.3	7.0	odd	ok
	8	C 26 H 27 N 4 O 2 S	94.83	459.1849	-0.9	-1.1	26.1	15.5	even	ok

Figure S54. HRMS spectrum of **18**

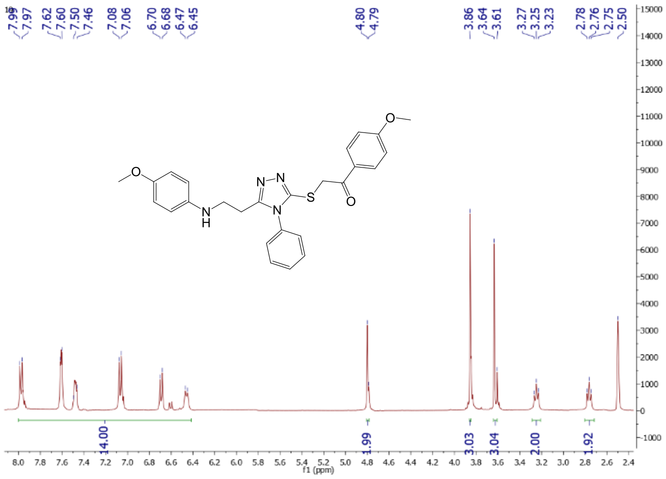

Figure S55. ^{1}H-NMR (400 MHz, DMSO-d_6) spectrum of **19**

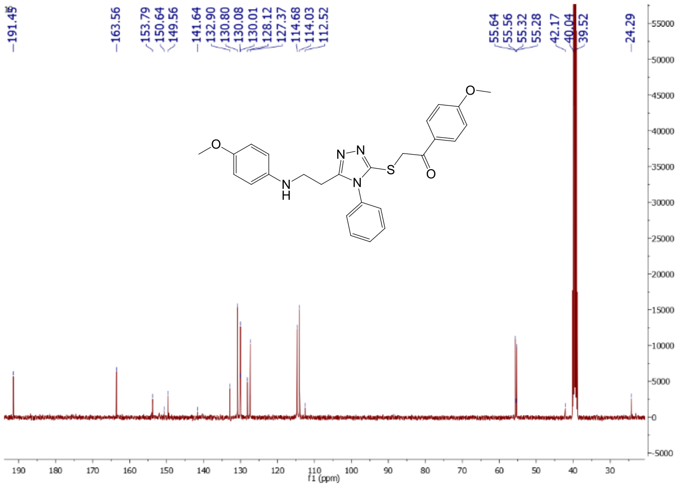

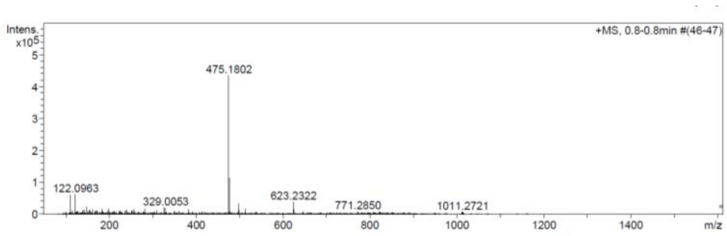

Meas. m/z	#	Formula	Score	m/z	err [ppm]	Mean err [ppm]	mSigma	rdb	e⁻ Conf	N-Rule
475.1802	1	C 21 H 31 O 12	96.70	475.1810	1.8	2.6	14.4	6.5	even	ok
	2	C 20 H 25 N 7 O 7	95.73	475.1810	1.8	2.6	15.0	12.0	odd	ok
	3	C 19 H 19 N 14 O 2	95.62	475.1810	1.7	2.5	15.2	17.5	even	ok
	4	C 17 H 17 N 17 O	100.00	475.1796	-1.1	-0.3	20.9	18.0	odd	ok
	5	C 18 H 23 N 10 O 6	99.12	475.1797	-1.1	-0.3	21.4	12.5	even	ok
	6	C 25 H 31 O 7 S	50.31	475.1785	-3.5	-3.7	21.4	10.5	even	ok
	7	C 19 H 29 N 3 O 11	98.40	475.1797	-1.1	-0.3	21.8	7.0	odd	ok
	8	C 24 H 25 N 7 O 2 S	49.20	475.1785	-3.5	-3.8	22.3	16.0	odd	ok
	9	C 17 H 27 N 6 O 10	38.31	475.1783	-3.9	-3.1	27.7	7.5	even	ok
	10	C 26 H 27 N 4 O 3 S	92.92	475.1798	-0.7	-0.9	28.0	15.5	even	ok

Figure S57. HRMS spectrum of **19**

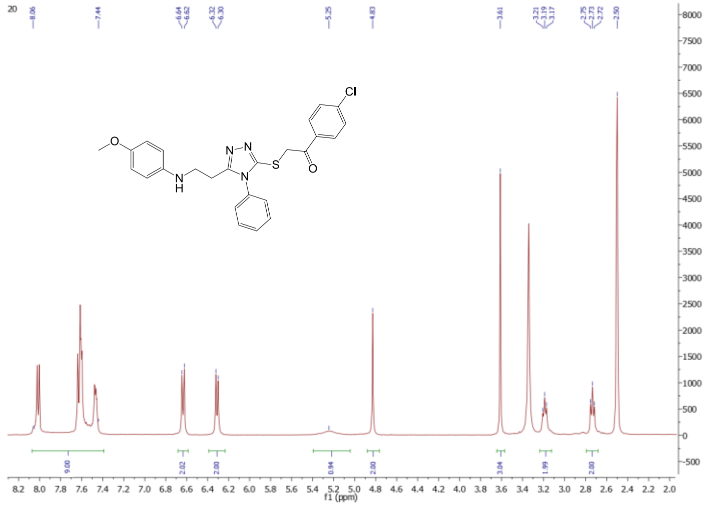

Figure S58. ^{1}H-NMR (400 MHz, DMSO-*d*6) spectrum of **20**

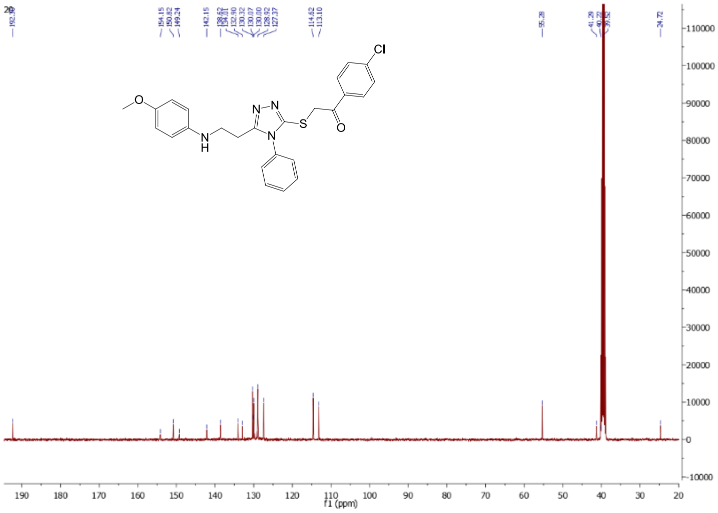

Figure S59. ^{13}C-NMR (400 MHz, DMSO-*d*6) spectrum of **20**

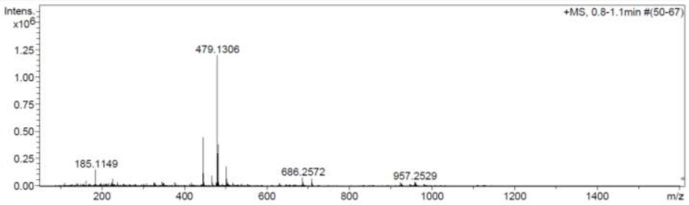

Meas. m/z	#	Formula	Score	m/z	err [ppm]	Mean err [ppm]	mSigma	rdb	e⁻ Conf	N-Rule
445.1694	1	C 24 H 29 O 6 S	54.34	445.1679	-3.3	-3.5	17.0	10.5	even	ok
	2	C 18 H 17 N 14 O	68.65	445.1704	2.3	3.0	19.9	17.5	even	ok
	3	C 25 H 25 N 4 O 2 S	100.00	445.1693	-0.3	-0.6	22.2	15.5	even	ok
	4	C 20 H 29 O 11	64.37	445.1704	2.3	3.1	22.6	6.5	even	ok
	5	C 17 H 21 N 10 O 5	80.27	445.1691	-0.7	0.1	28.3	12.5	even	ok
	6	C 18 H 25 N 10 S 2	67.04	445.1700	1.2	0.9	31.7	11.5	even	ok
	7	C 17 H 29 N 6 O 4 S 2	49.88	445.1686	-1.8	-2.1	38.0	6.5	even	ok
	8	C 21 H 33 O 6 S 2	24.97	445.1713	4.2	4.0	38.0	5.5	even	ok
	9	C 33 H 21 N 2	39.92	445.1699	1.1	2.2	50.9	24.5	even	ok
	10	C 27 H 26 Cl N 2 O 2	0.25	445.1677	-3.8	-3.9	144.4	15.5	even	ok
	11	C 24 H 30 Cl N 2 O 2 S	0.22	445.1711	3.8	3.7	146.7	10.5	even	ok
	12	C 20 H 26 Cl N 8 S	0.19	445.1684	-2.3	-2.4	155.3	11.5	even	ok
479.1306	1	C 18 H 16 Cl N 14 O	93.31	479.1315	1.8	2.1	16.6	17.5	even	ok
	2	C 17 H 20 Cl N 10 O 5	100.00	479.1301	-1.0	-0.6	21.6	12.5	even	ok
	3	C 19 H 11 N 16 O	76.99	479.1296	-2.1	-1.9	21.8	22.5	even	ok

Figure S60. HRMS spectrum of **20**

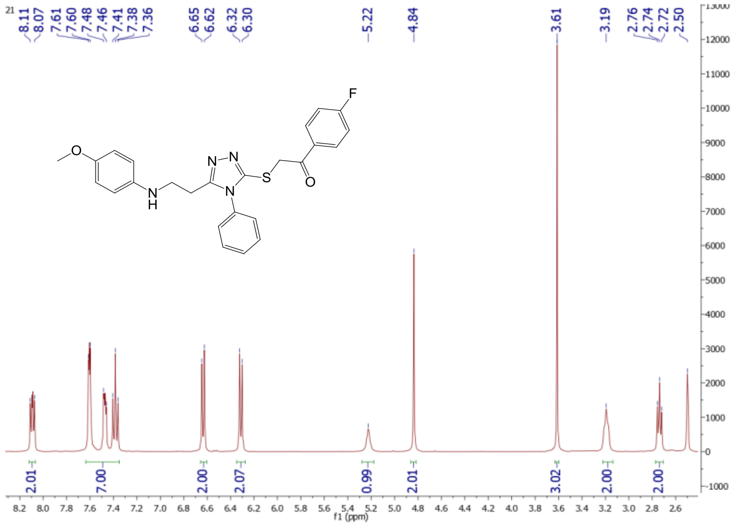

Figure S61. ^{1}H-NMR (400 MHz, DMSO-d_6) spectrum of **21**

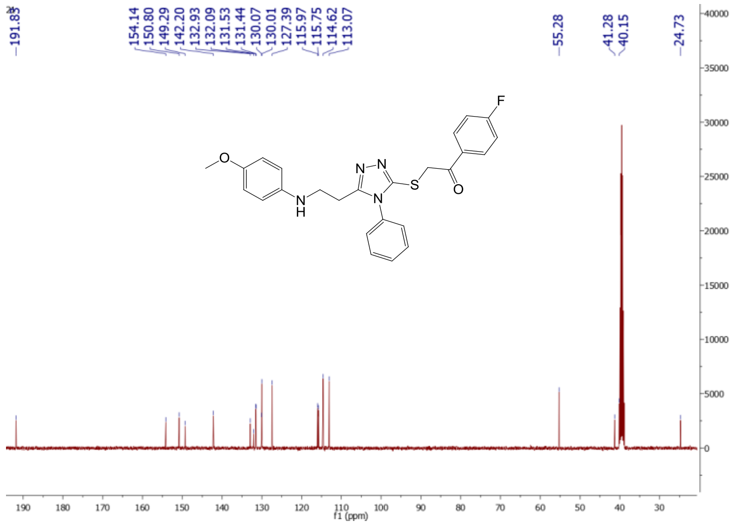

Figure S62. ^{13}C-NMR (400 MHz, DMSO-d_6) spectrum of **21**

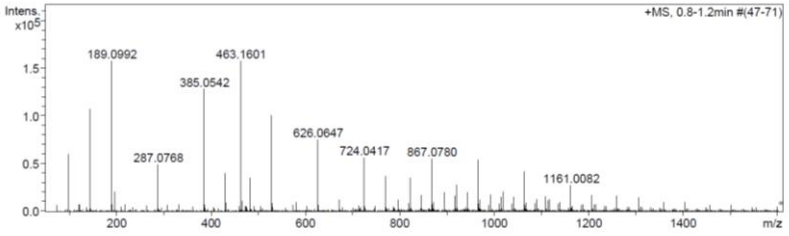

Meas. m/z	#	Formula	Score	m/z	err [ppm]	Mean err [ppm]	mSigma	rdb	e⁻ Conf	N-Rule
189.0992	1	C 4 H 10 F N 8	64.61	189.1007	7.9	8.0	16.3	3.5	even	ok
	2	C 6 H 13 N 4 O 3	82.39	189.0982	-5.2	-5.1	19.3	2.5	even	ok
	3	C 8 H 15 N O 4	100.00	189.0996	1.9	2.0	25.4	2.0	odd	ok

Figure S63. HRMS spectrum of **21**

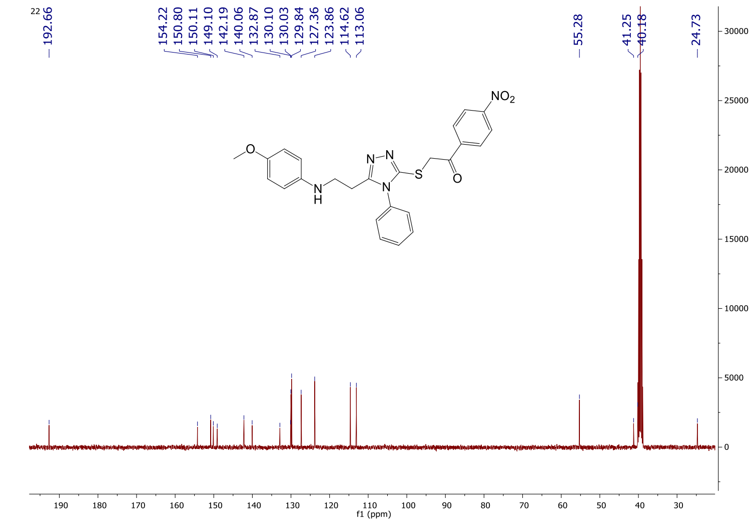

Figure S64. ^{1}H-NMR (400 MHz, DMSO-d_6) spectrum of **22**

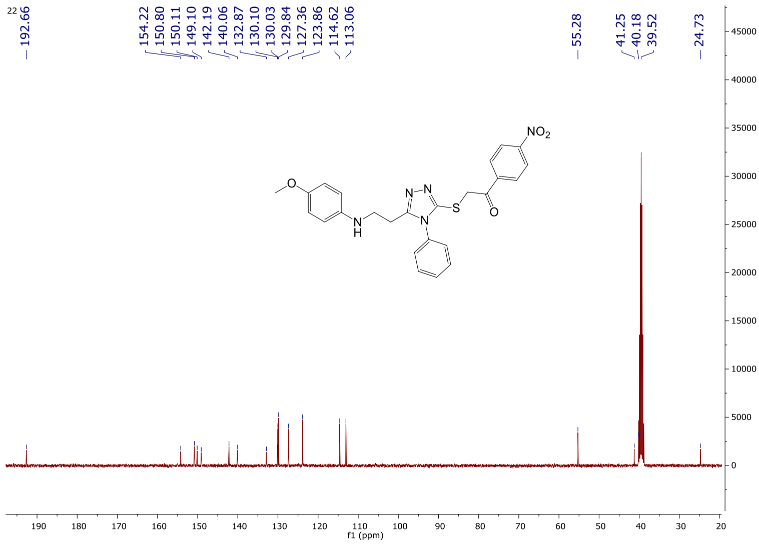

Figure S65. ^{13}C-NMR (400 MHz, DMSO-d_6) spectrum of **22**

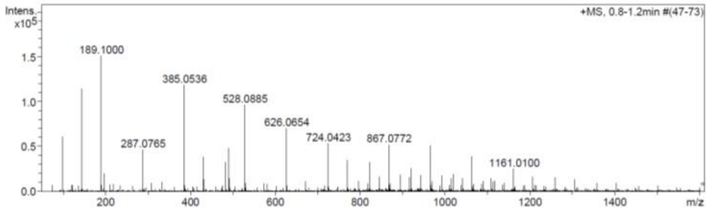

Meas. m/z	#	Formula	Score	m/z	err [ppm]	Mean err [ppm]	mSigma	rdb	e⁻ Conf	N-Rule
	32	C 34 H 3 N 4 O	0.21	483.0301	-0.4	-0.2	159.1	35.5	even	ok
	33	C 36 H 5 N O 2	0.08	483.0315	2.4	2.6	164.9	35.0	odd	ok
490.1542	1	C 23 H 22 N 8 O 3 S	60.88	490.1530	-2.4	-2.8	9.7	17.0	odd	ok
	2	C 25 H 24 N 5 O 4 S	100.00	490.1544	0.3	-0.1	10.7	16.5	even	ok

Figure S66. HRMS spectrum of **22**

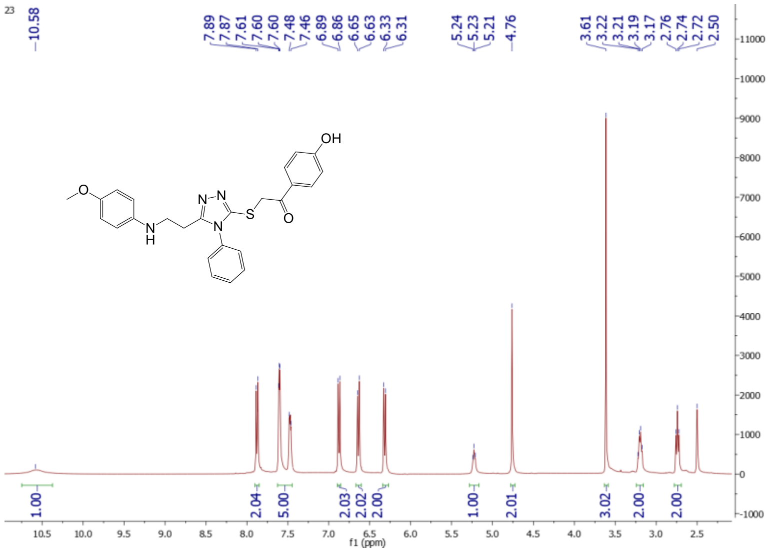

Figure S67. ^{1}H-NMR (400 MHz, DMSO-d_6) spectrum of **23**

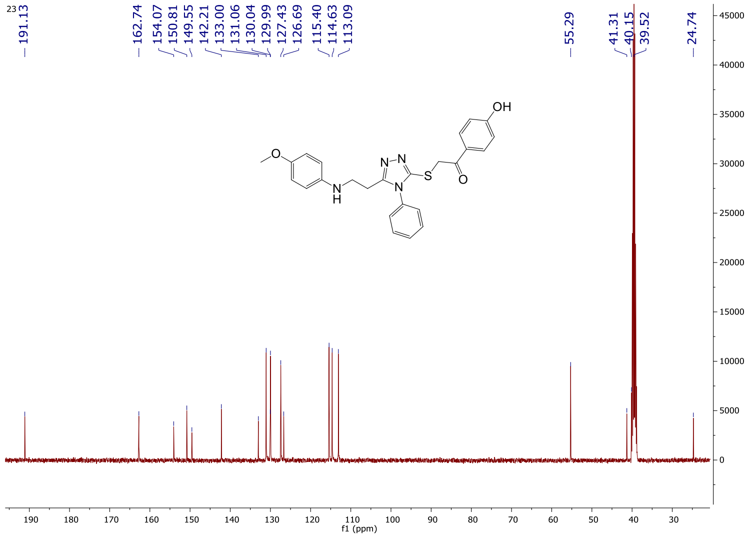

Figure S68. ^{13}C-NMR (400 MHz, DMSO-d_6) spectrum of **23**

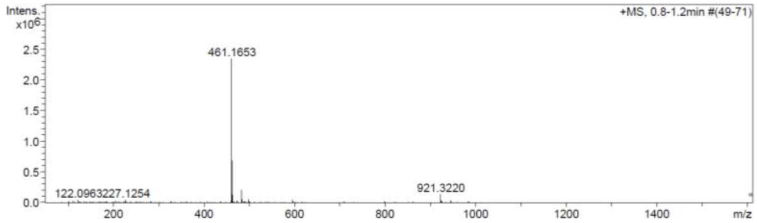

Meas. m/z	#	Formula	Score	m/z	err [ppm]	Mean err [ppm]	mSigma	rdb	e⁻ Conf	N-Rule
461.1653	1	C 25 H 25 N 4 O 3 S	69.71	461.1642	-2.4	-2.4	19.8	15.5	even	ok
	2	C 27 H 27 N O 4 S	100.00	461.1655	0.5	0.6	23.1	15.0	odd	ok
	3	C 20 H 19 N 11 O 3	52.90	461.1667	3.0	3.9	23.7	17.0	odd	ok
	4	C 21 H 25 N 4 O 8	48.79	461.1667	3.1	4.1	27.0	11.5	even	ok
	5	C 28 H 23 N 5 S	42.03	461.1669	3.4	3.5	28.4	20.0	odd	ok

Figure S69. HRMS spectrum of **23**

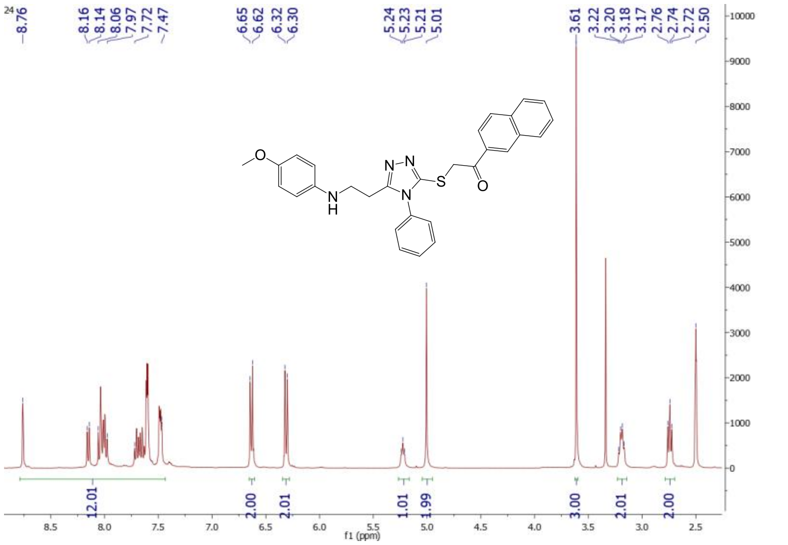

Figure S70. ^{1}H-NMR (400 MHz, DMSO-*d*6) spectrum of **24**

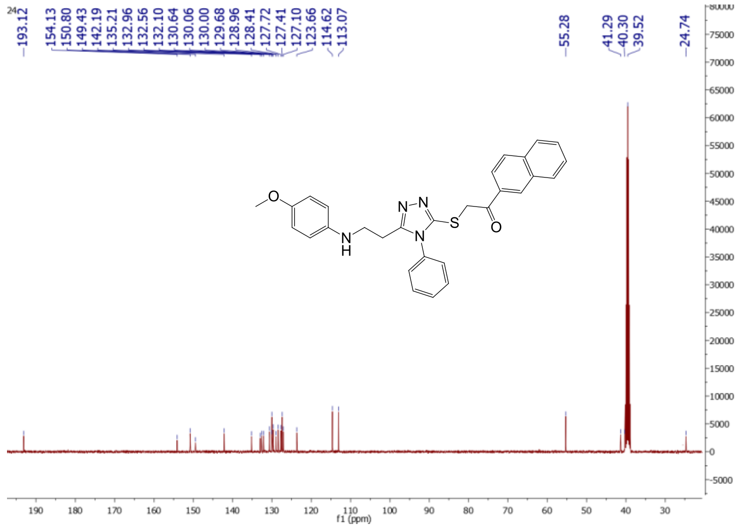

Figure S71. ^{13}C-NMR (400 MHz, DMSO-*d*6) spectrum of **24**

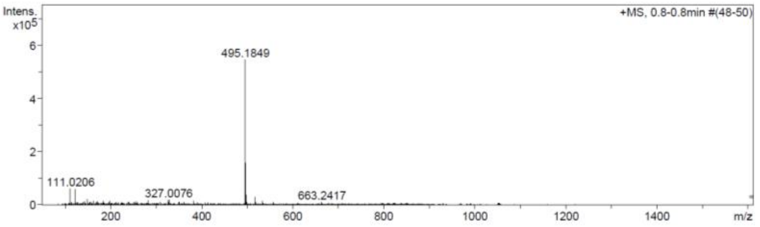

Meas. m/z	#	Formula	Score	m/z	err [ppm]	Mean err [ppm]	mSigma	rdb	e⁻ Conf	N-Rule
495.1849	1	C 24 H 31 O 11	66.32	495.1861	2.4	3.3	12.6	9.5	even	ok
	2	C 23 H 25 N 7 O 6	65.97	495.1861	2.4	3.1	13.1	15.0	odd	ok
	3	C 22 H 19 N 14 O	63.14	495.1861	2.4	3.0	15.4	20.5	even	ok
	4	C 20 H 17 N 17	100.00	495.1847	-0.3	0.3	18.3	21.0	odd	ok
	5	C 21 H 23 N 10 O 5	97.99	495.1847	-0.3	0.4	19.4	15.5	even	ok
	6	C 22 H 29 N 3 O 10	96.70	495.1847	-0.3	0.6	20.1	10.0	odd	ok
	7	C 28 H 31 O 6 S	48.75	495.1836	-2.7	-3.0	23.7	13.5	even	ok
	8	C 27 H 25 N 7 O S	48.17	495.1836	-2.7	-3.0	24.0	19.0	odd	ok
	9	C 20 H 27 N 6 O 9	39.45	495.1834	-3.0	-2.2	27.7	10.5	even	ok
	10	C 29 H 27 N 4 O 2 S	80.22	495.1849	0.0	0.7	30.6	18.5	even	ok

Figure S72. HRMS spectrum of **24**

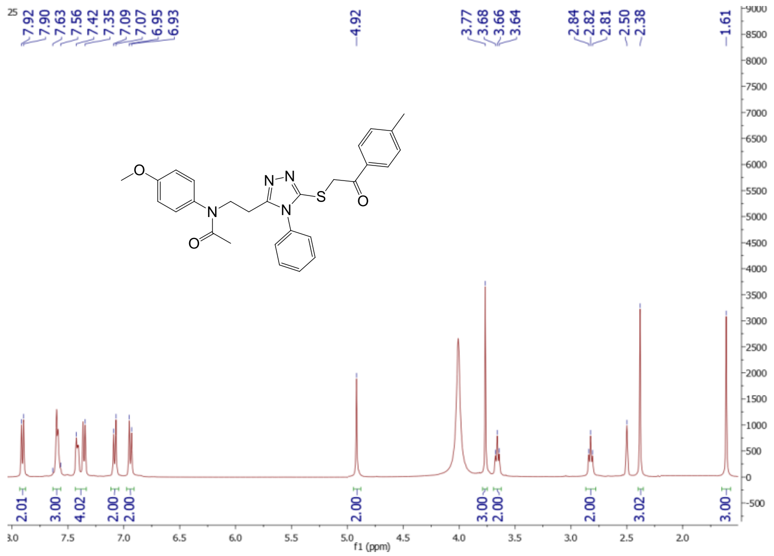

Figure S73. ^{1}H-NMR (400 MHz, DMSO-*d*$_6$) spectrum of **25**

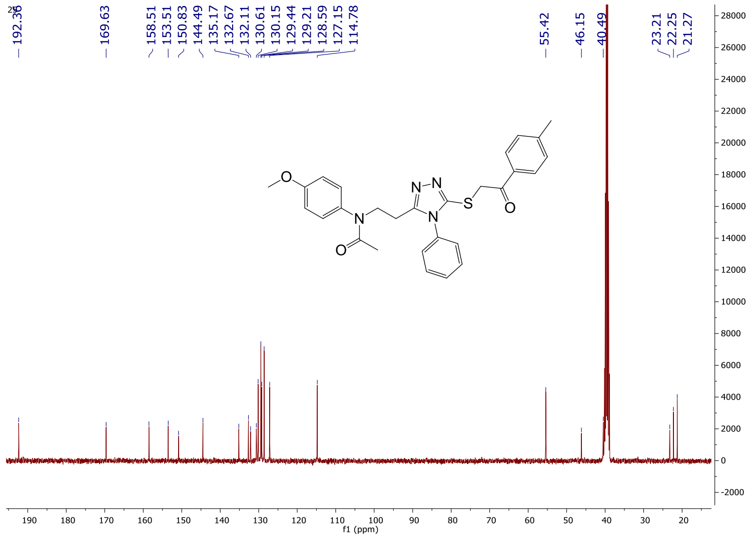

Figure S74. ^{13}C-NMR (400 MHz, DMSO-*d*$_6$) spectrum of **25**

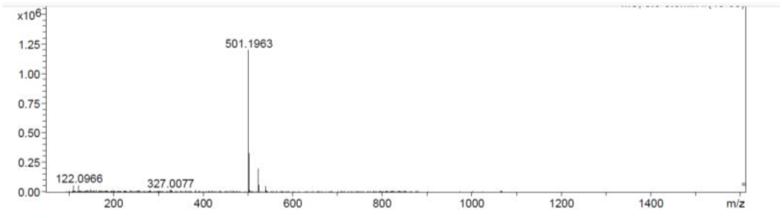

Meas. m/z	#	Formula	Score	m/z	err [ppm]	Mean err [ppm]	mSigma	rdb	e⁻ Conf	N-Rule
501.1963	1	C 24 H 29 N 4 O 8	48.77	501.1980	3.5	4.3	4.9	12.5	even	ok
	2	C 22 H 27 N 7 O 7	100.00	501.1966	0.8	1.5	8.6	13.0	odd	ok
	3	C 23 H 33 O 12	97.67	501.1967	0.8	1.7	9.7	7.5	even	ok
	4	C 21 H 21 N 14 O 2	97.92	501.1966	0.8	1.4	9.8	18.5	even	ok
	5	C 23 H 23 N 11 O 3	44.29	501.1980	3.4	4.1	10.4	18.0	odd	ok
	6	C 19 H 19 N 17 O	66.85	501.1953	-1.9	-1.3	14.5	19.0	odd	ok
	7	C 20 H 25 N 10 O 6	66.13	501.1953	-1.9	-1.2	15.2	13.5	even	ok
	8	C 21 H 31 N 3 O 11	66.11	501.1953	-1.9	-1.0	15.3	8.0	odd	ok
	9	C 28 H 29 N 4 O 3 S	47.38	501.1955	-1.5	-1.8	34.2	16.5	even	ok

Figure S75. HRMS spectrum of **25**

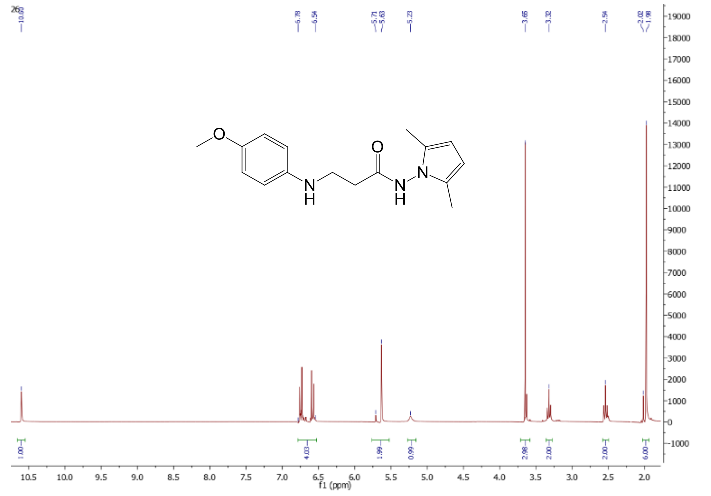

Figure S76. ^{1}H-NMR (400 MHz, DMSO-d_6) spectrum of **26**

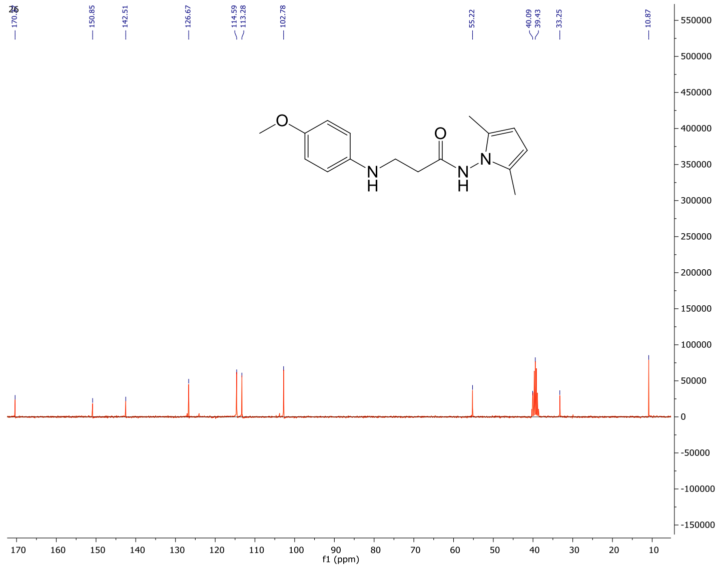

Figure S77. ^{13}C-NMR (400 MHz, DMSO-d_6) spectrum of **26**

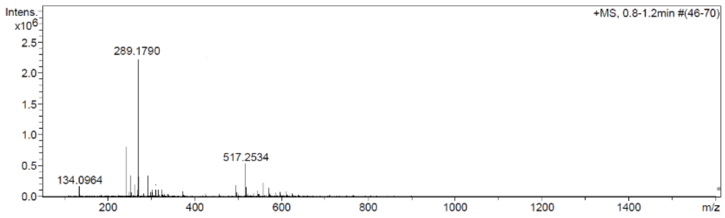

Meas. m/z	#	Formula	Score	m/z	err [ppm]	Mean err [ppm]	mSigma	rdb	e⁻ Conf	N-Rule
248.1397	1	C 13 H 18 N 3 O 2	100.00	248.1394	-1.3	-1.0	6.6	6.5	even	ok
270.1213	1	C 10 H 16 N 5 O 4	55.55	270.1197	-6.1	-5.9	2.9	5.5	even	ok
	2	C 11 H 12 N 9	100.00	270.1210	-1.1	-0.8	12.3	10.5	even	ok
276.1706	1	C 15 H 22 N 3 O 2	100.00	276.1707	0.2	0.4	10.5	6.5	even	ok
289.1790	1	C 16 H 22 N 3 O 2	100.00	289.1790	-0.2	0.1	23.2	7.5	even	ok

Figure S78. HRMS spectrum of **26**

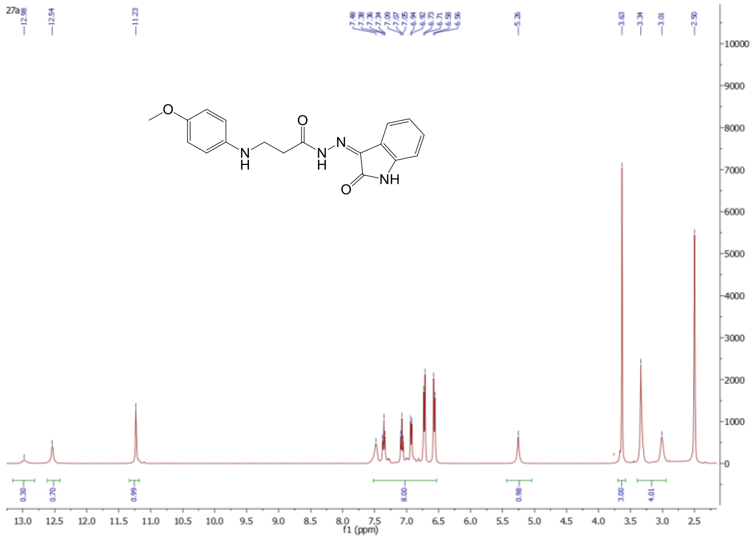

Figure S79. ^{1}H-NMR (400 MHz, DMSO-*d*$_6$) spectrum of **27a**

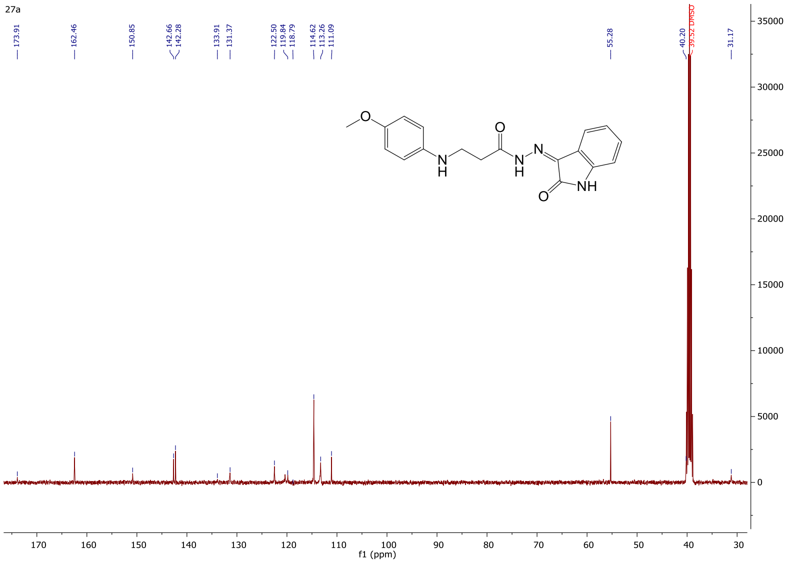

Figure S80. ^{13}C-NMR (400 MHz, DMSO-*d*$_6$) spectrum of **27a**

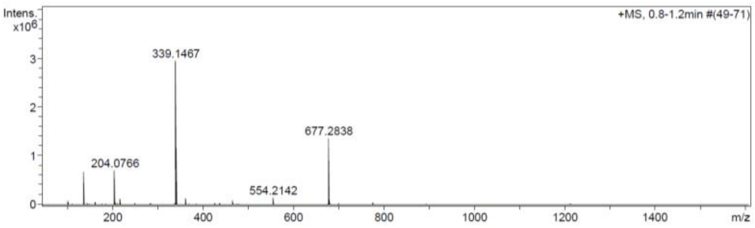

Meas. m/z	#	Formula	Score	m/z	err [ppm]	Mean err [ppm]	mSigma	rdb	e⁻ Conf	N-Rule
136.0756	1	C 8 H 10 N O	100.00	136.0757	0.3	0.5	3.4	4.5	even	ok
204.0766	1	C 10 H 10 N 3 O 2	100.00	204.0768	0.5	0.7	12.7	7.5	even	ok
339.1467	1	C 4 H 11 N 20	100.00	339.1470	1.0	0.6	70.8	9.5	even	ok
	2	C 18 H 19 N 4 O 3	19.25	339.1452	-4.4	-2.9	94.9	11.5	even	ok
[illegible]	[illegible]	[illegible]	[illegible]	[illegible]	[illegible]	[illegible]	[illegible]	[illegible]	[illegible]	[illegible]

Figure S81. HRMS spectrum of **27a**

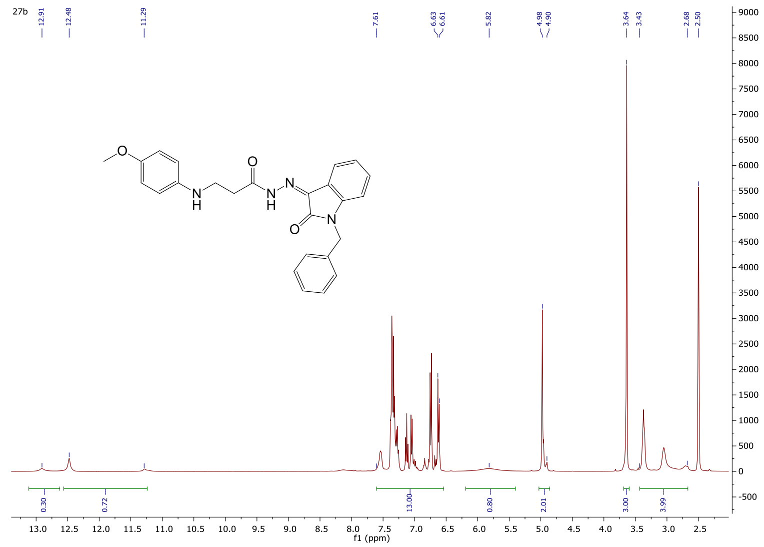

Figure S82. ^{1}H-NMR (400 MHz, DMSO-d_6) spectrum of **27b**

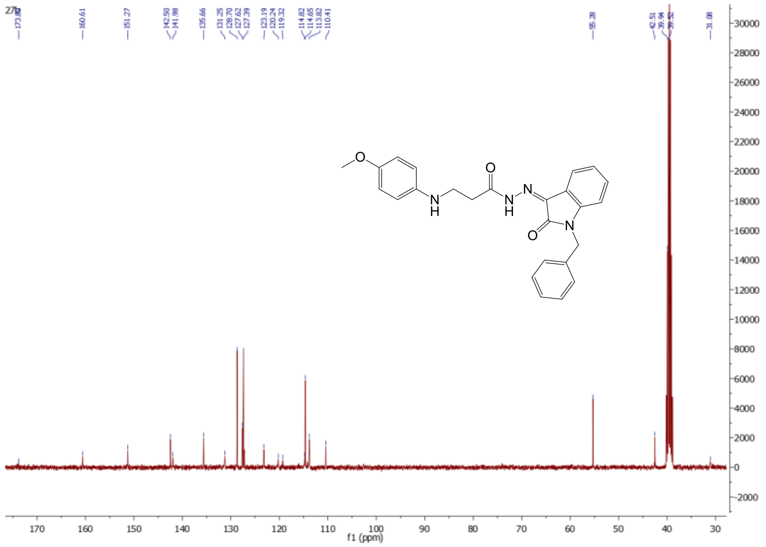

Figure S83. ^{13}C-NMR (400 MHz, DMSO-d_6) spectrum of **27b**

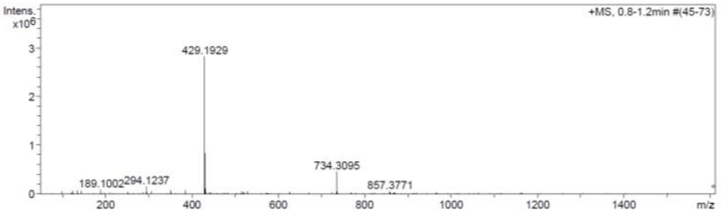

Meas. m/z	#	Formula	Score	m/z	err [ppm]	Mean err [ppm]	mSigma	rdb	e⁻ Conf	N-Rule
429.1929	1	C 27 H 27 N O 4	100.00	429.1935	1.3	1.9	6.3	15.0	odd	ok
	2	C 25 H 25 N 4 O 3	86.83	429.1921	-1.8	-1.3	7.9	15.5	even	ok
	3	C 28 H 23 N 5	38.35	429.1948	4.4	4.9	13.7	20.0	odd	ok

Figure S84. HRMS spectrum of **27b**

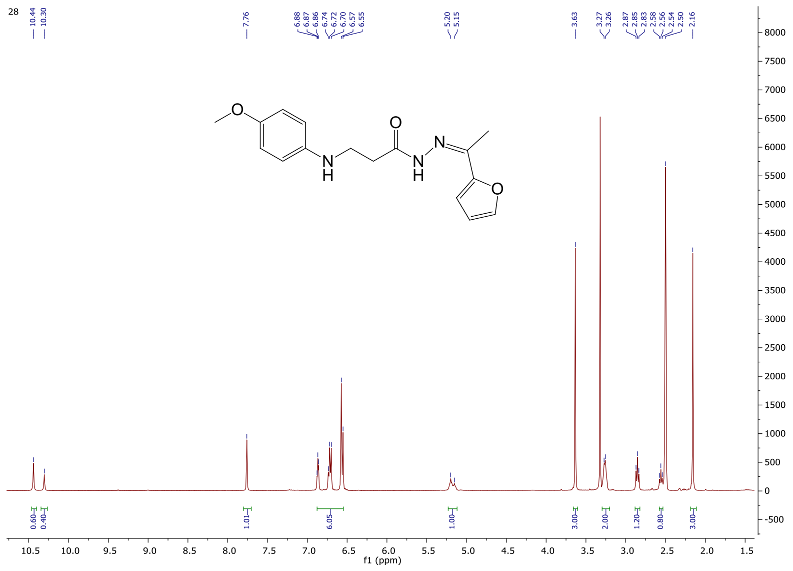

Figure S85. ^{1}H-NMR (400 MHz, DMSO-*d*6) spectrum of **28**

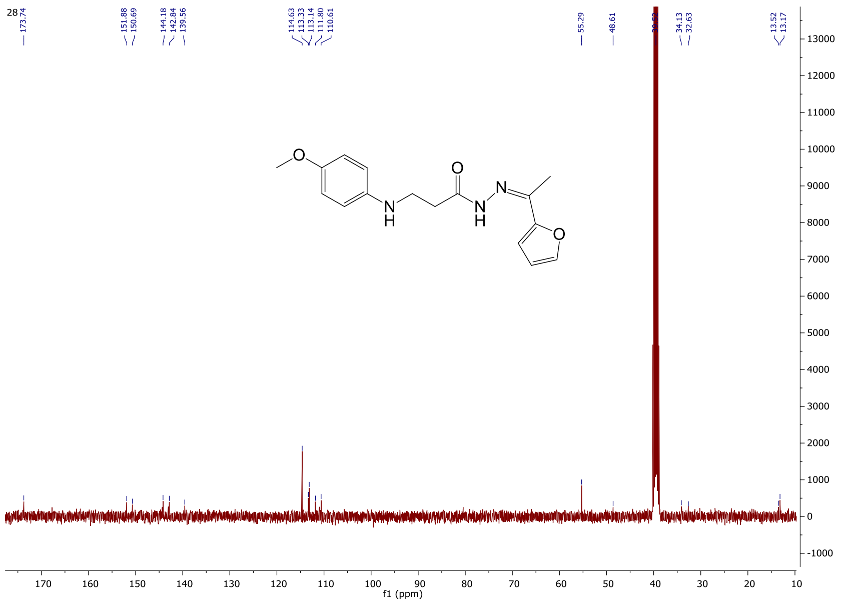

Figure S86. ^{13}C-NMR (400 MHz, DMSO-*d*6) spectrum of **28**

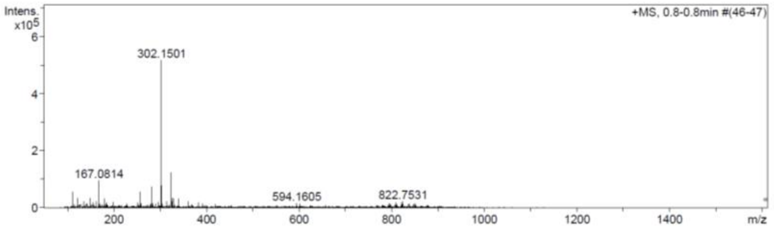

Meas. m/z	#	Formula	Score	m/z	err [ppm]	Mean err [ppm]	mSigma	rdb	e⁻ Conf	N-Rule
302.1501	1	C 14 H 18 N 6 O 2	54.45	302.1486	-5.1	-4.9	14.0	9.0	odd	ok
	2	C 16 H 20 N 3 O 3	100.00	302.1499	-0.7	-0.4	20.3	8.5	even	ok
	3	C 18 H 22 O 4	53.34	302.1513	3.8	4.1	26.6	8.0	odd	ok

Figure S87. HRMS spectrum of **28**

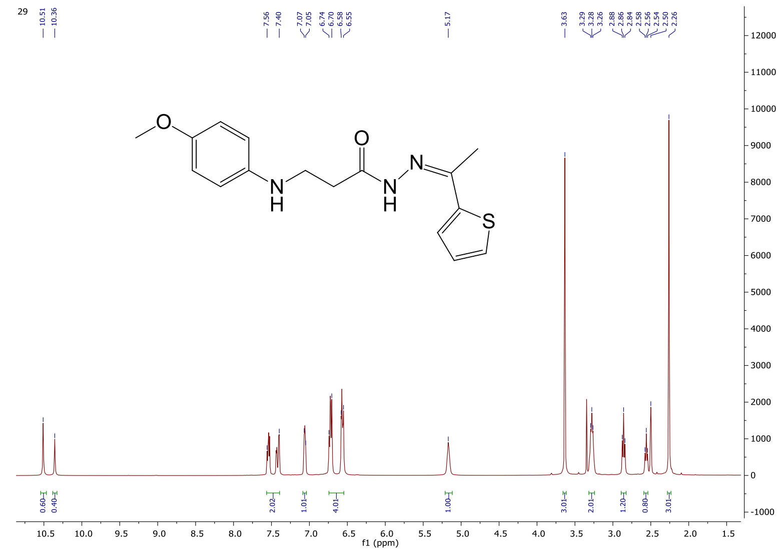

Figure S88. ^{1}H-NMR (400 MHz, DMSO-d_6) spectrum of **29**

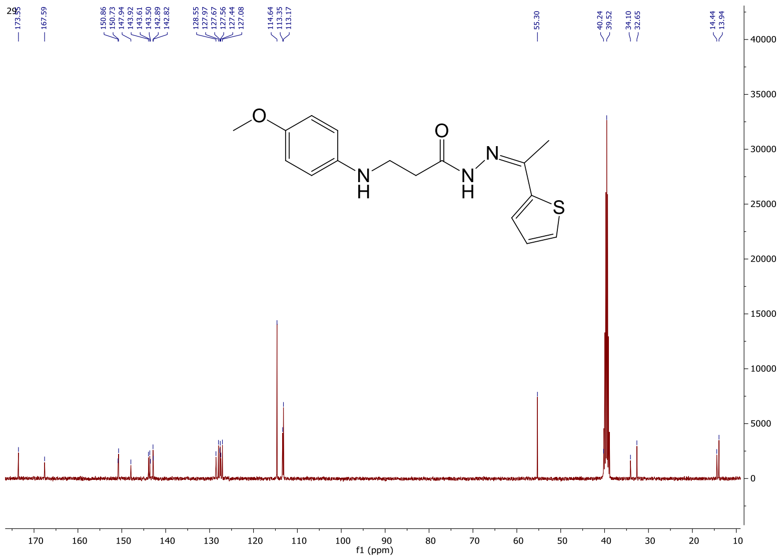

Figure S89. ^{13}C-NMR (400 MHz, DMSO-d_6) spectrum of **29**

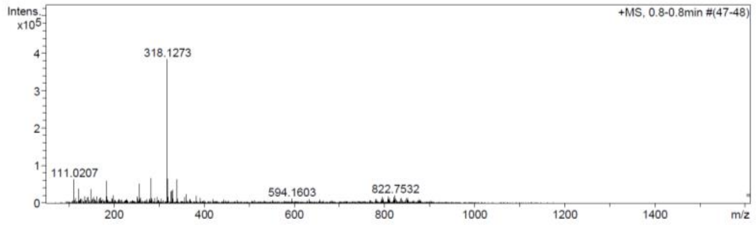

Meas. m/z	#	Formula	Score	m/z	err [ppm]	Mean err [ppm]	mSigma	rdb	e⁻ Conf	N-Rule
318.1273	1	C 14 H 18 N 6 O S	53.07	318.1257	-4.9	-5.0	12.3	9.0	odd	ok
	2	C 16 H 20 N 3 O 2 S	100.00	318.1271	-0.7	-0.7	17.8	8.5	even	ok
	3	C 18 H 22 O 3 S	56.92	318.1284	3.5	3.5	22.9	8.0	odd	ok
	4	C 10 H 18 N 6 O 6	60.01	318.1282	2.9	3.8	25.2	5.0	odd	ok

Figure S90. HRMS spectrum of **29**

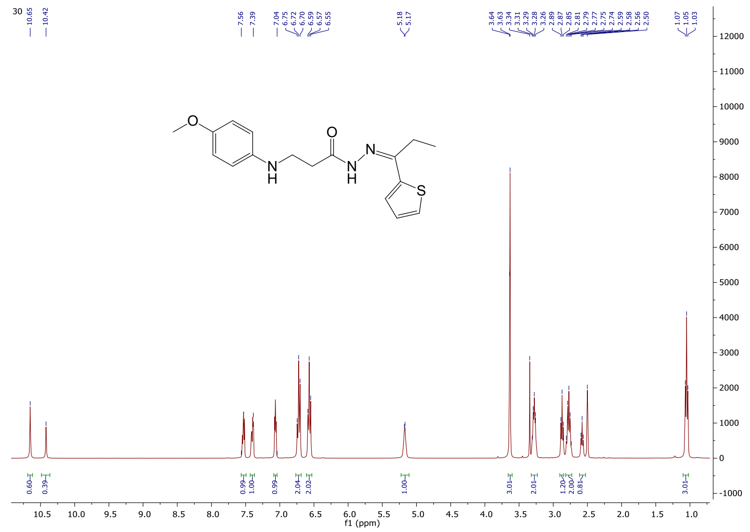

Figure S91. ^{1}H-NMR (400 MHz, DMSO-d_6) spectrum of **30**

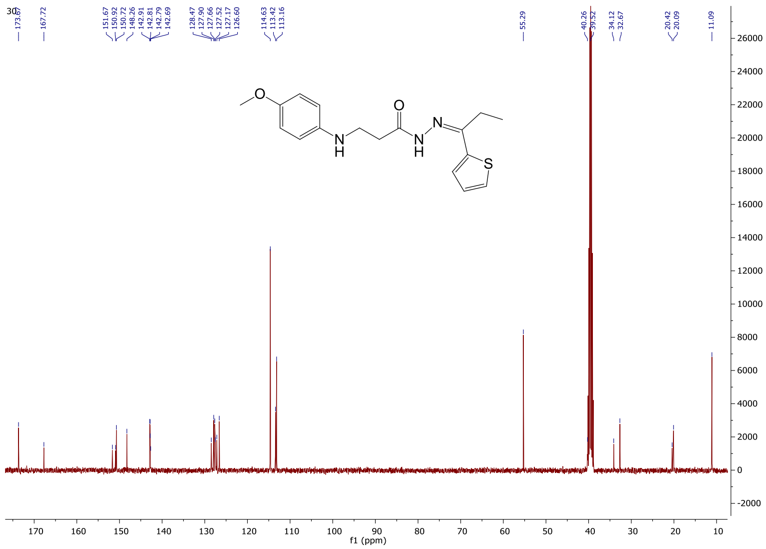

Figure S92. ^{13}C-NMR (400 MHz, DMSO-d_6) spectrum of **30**

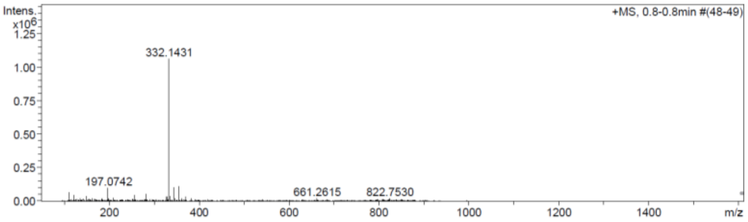

Meas. m/z	#	Formula	Score	m/z	err [ppm]	Mean err [ppm]	mSigma	rdb	e⁻ Conf	N-Rule
332.1431	1	C 11 H 20 N 6 O 6	96.89	332.1439	2.5	3.2	12.9	5.0	odd	ok
	2	C 10 H 14 N 13 O	95.01	332.1439	2.5	3.4	14.1	10.5	even	ok
	3	C 9 H 18 N 9 O 5	100.00	332.1425	-1.6	-0.8	19.2	5.5	even	ok
	4	C 15 H 20 N 6 O S	47.80	332.1414	-5.0	-5.0	21.7	9.0	odd	ok
	5	C 10 H 24 N 2 O 10	91.45	332.1425	-1.5	-0.7	23.4	0.0	odd	ok
	6	C 17 H 22 N 3 O 2 S	89.83	332.1427	-1.0	-1.0	27.8	8.5	even	ok

Figure S93. HRMS spectrum of **30**

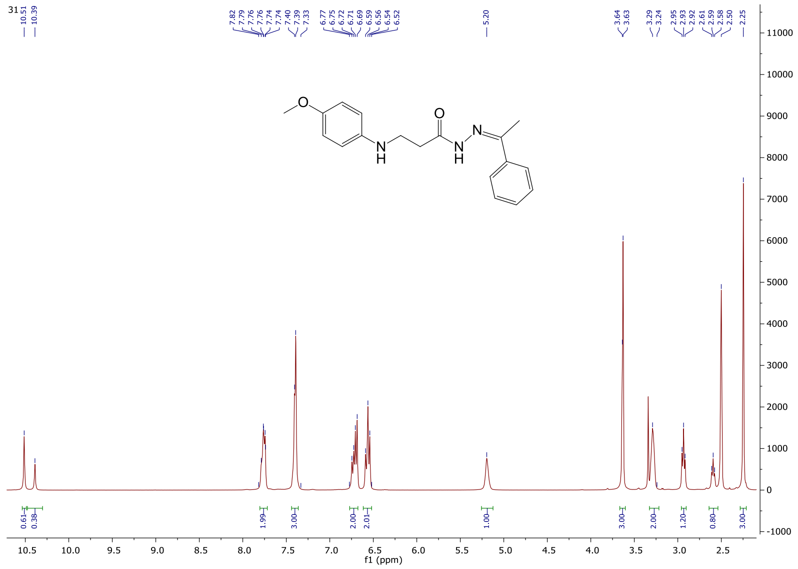

Figure S94. ^{1}H-NMR (400 MHz, DMSO-*d*$_6$) spectrum of **31**

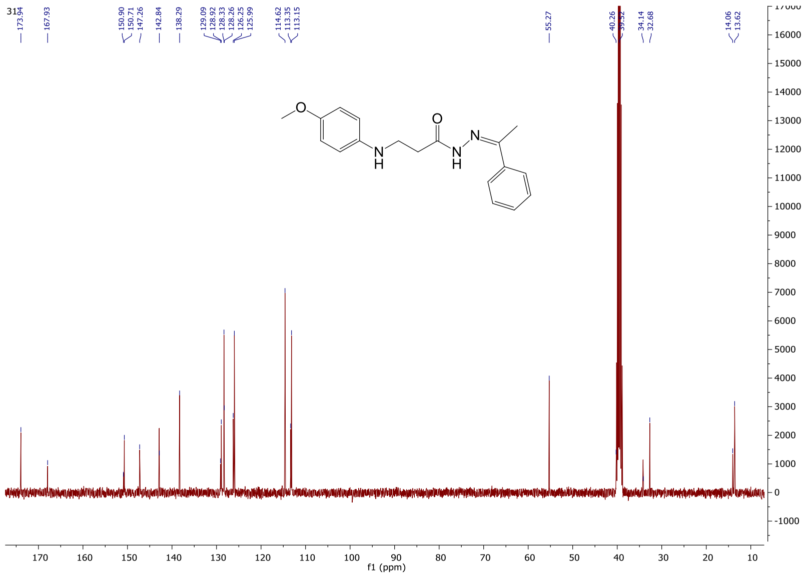

Figure S95. ^{13}C-NMR (400 MHz, DMSO-*d*$_6$) spectrum of **31**

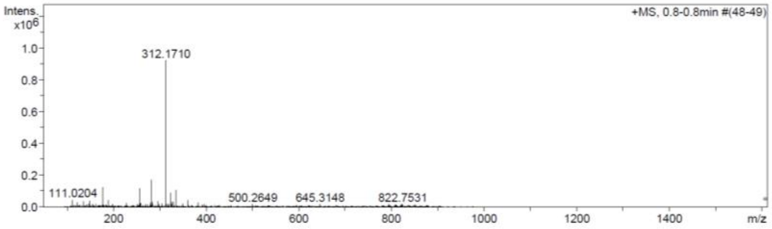

Meas. m/z	#	Formula	Score	m/z	err [ppm]	Mean err [ppm]	mSigma	rdb	e⁻ Conf	N-Rule
312.1710	1	C 16 H 20 N 6 O	52.45	312.1693	-5.5	-5.2	15.5	10.0	odd	ok
	2	C 18 H 22 N 3 O 2	100.00	312.1707	-1.2	-0.9	21.7	9.5	even	ok
	3	C 20 H 24 O 3	63.02	312.1720	3.1	3.5	28.1	9.0	odd	ok

Figure S96. HRMS spectrum of **31**

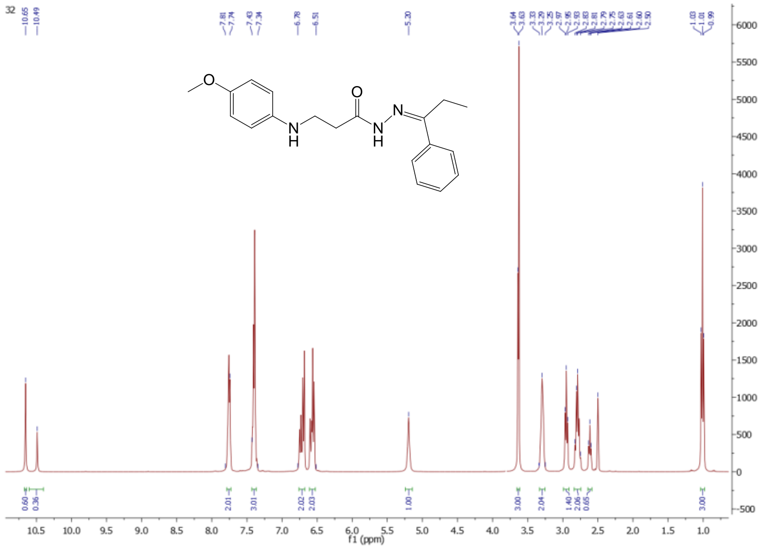

Figure S97. ^{1}H-NMR (400 MHz, DMSO-*d*6) spectrum of **32**

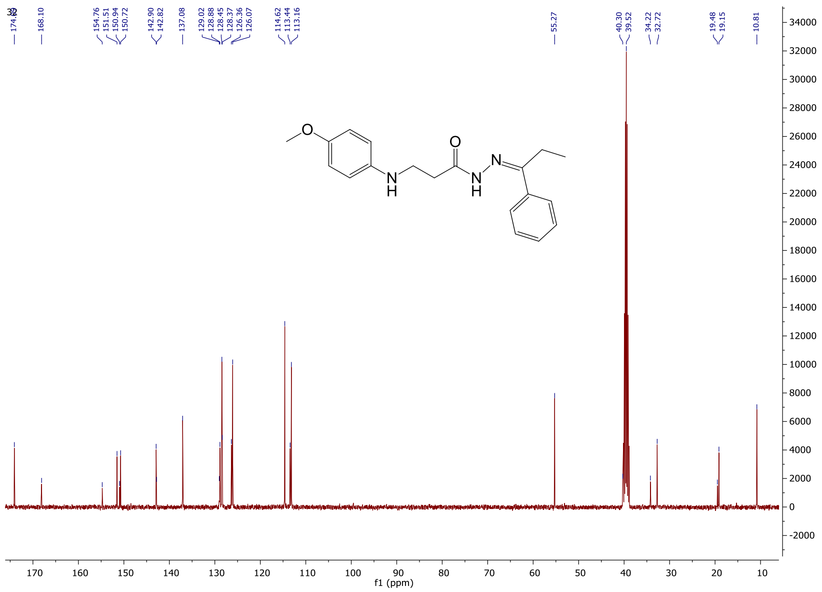

Figure S98. ^{13}C-NMR (400 MHz, DMSO-*d*6) spectrum of **32**

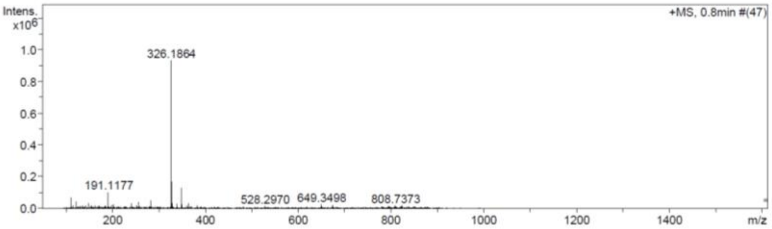

Meas. m/z	#	Formula	Score	m/z	err [ppm]	Mean err [ppm]	mSigma	rdb	e⁻ Conf	N-Rule
326.1864	1	C 17 H 22 N 6 O	55.84	326.1850	-4.5	-4.3	15.5	10.0	odd	ok
	2	C 19 H 24 N 3 O 2	100.00	326.1863	-0.4	-0.2	22.0	9.5	even	ok
	3	C 21 H 26 O 3	49.31	326.1876	3.7	4.0	28.5	9.0	odd	ok

Figure S99. HRMS spectrum of **32**

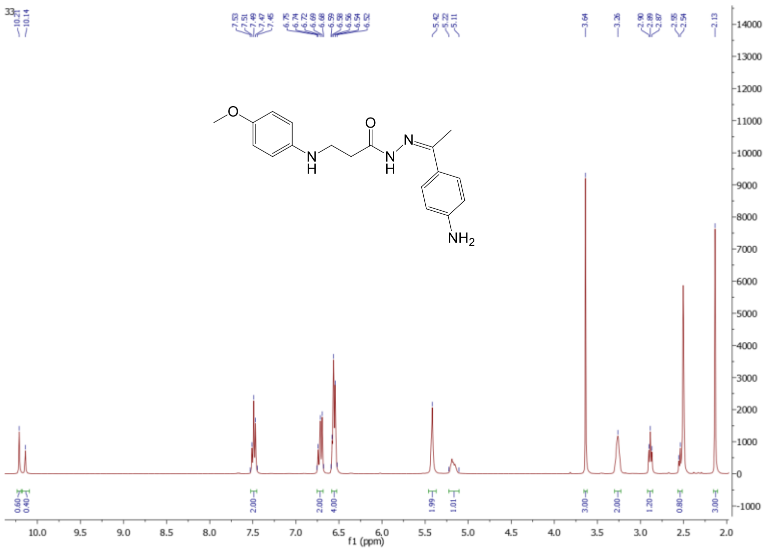

Figure S100. ^{1}H-NMR (400 MHz, DMSO-d_6) spectrum of **33**

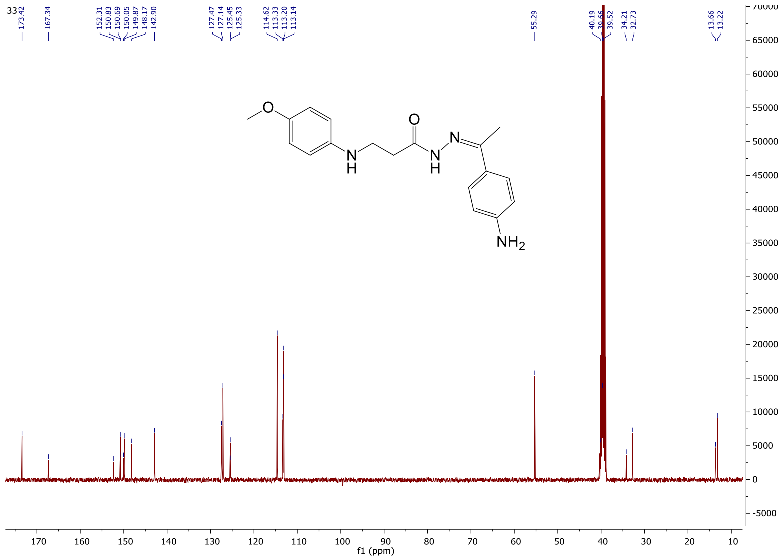

Figure S101. ^{13}C-NMR (400 MHz, DMSO-d_6) spectrum of **33**

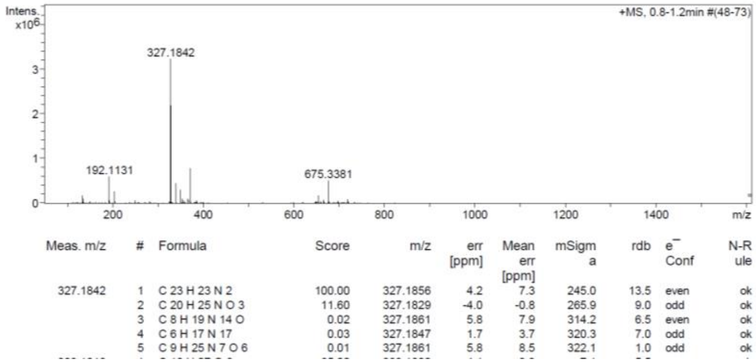

Figure S102. HRMS spectrum of **33**

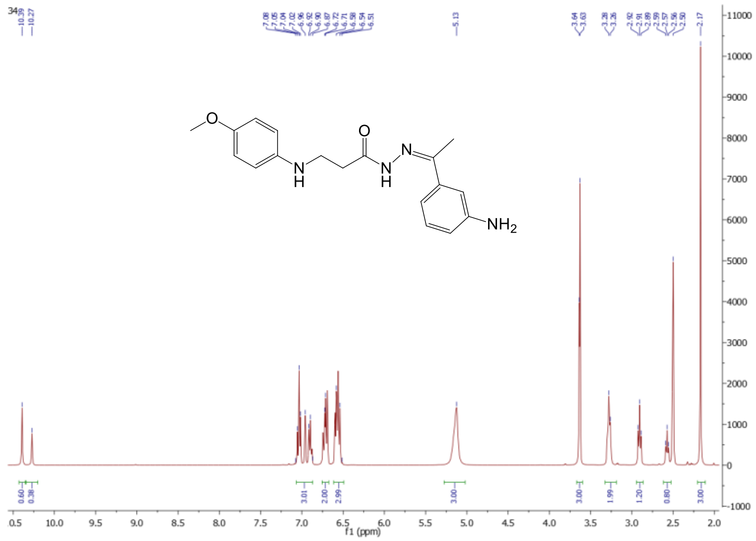

Figure S103. ^{1}H-NMR (400 MHz, DMSO-d_6) spectrum of **34**

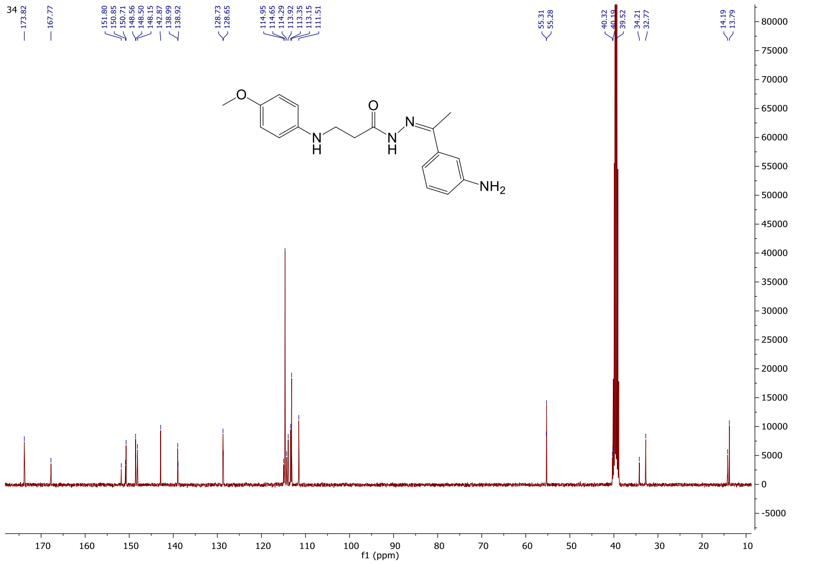

Figure S104. ^{13}C-NMR (400 MHz, DMSO-d_6) spectrum of **34**

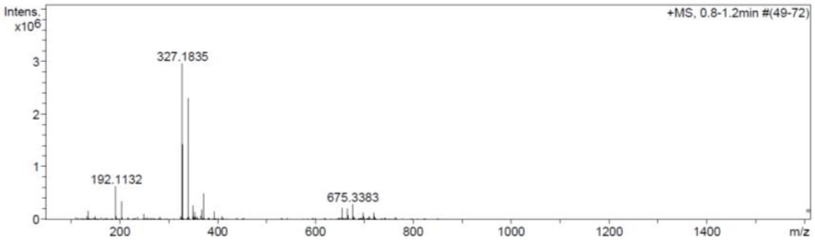

Meas. m/z	#	Formula	Score	m/z	err [ppm]	Mean err [ppm]	mSigma	rdb	e⁻ Conf	N-Rule
327.1835	1	C 20 H 25 N O 3	100.00	327.1829	-1.8	0.0	151.0	9.0	odd	ok
	2	C 18 H 23 N 4 O 2	28.76	327.1816	-5.9	-4.1	157.2	9.5	even	ok

Figure S105. HRMS spectrum of **34**

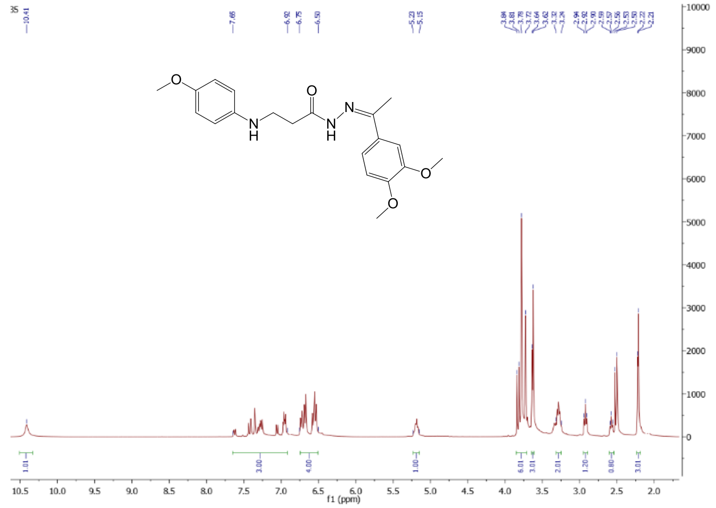

Figure S106. ^{1}H-NMR (400 MHz, DMSO-*d*6) spectrum of **35**

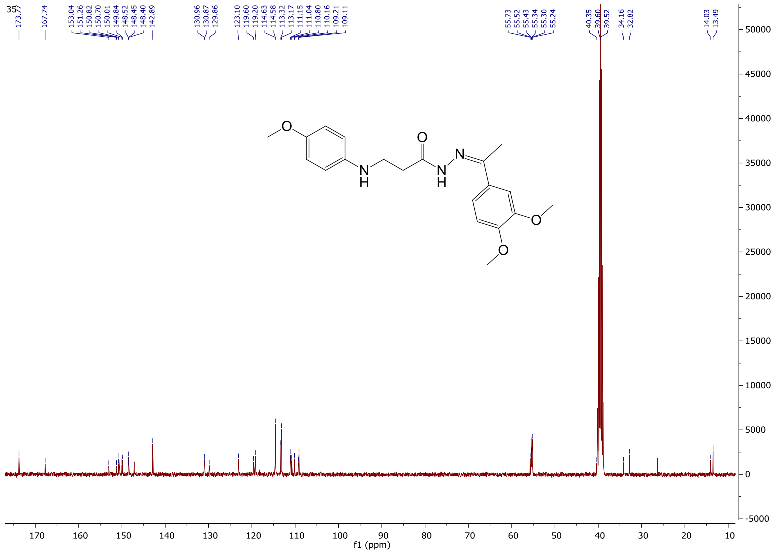

Figure S107. ^{13}C-NMR (400 MHz, DMSO-*d*6) spectrum of **35**

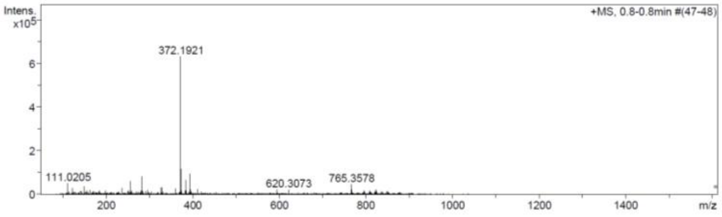

Meas. m/z	#	Formula	Score	m/z	err [ppm]	Mean err [ppm]	mSigma	rdb	e⁻ Conf	N-Rule
372.1921	1	C 18 H 24 N 6 O 3	53.93	372.1904	-4.4	-4.0	18.7	10.0	odd	ok
	2	C 20 H 26 N 3 O 4	100.00	372.1918	-0.7	-0.4	25.0	9.5	even	ok
	3	C 22 H 28 O 5	57.28	372.1931	2.9	3.2	31.4	9.0	odd	ok
	4	C 21 H 22 N 7	46.46	372.1931	2.9	3.2	39.6	14.5	even	ok

Figure S108. HRMS spectrum of **35**

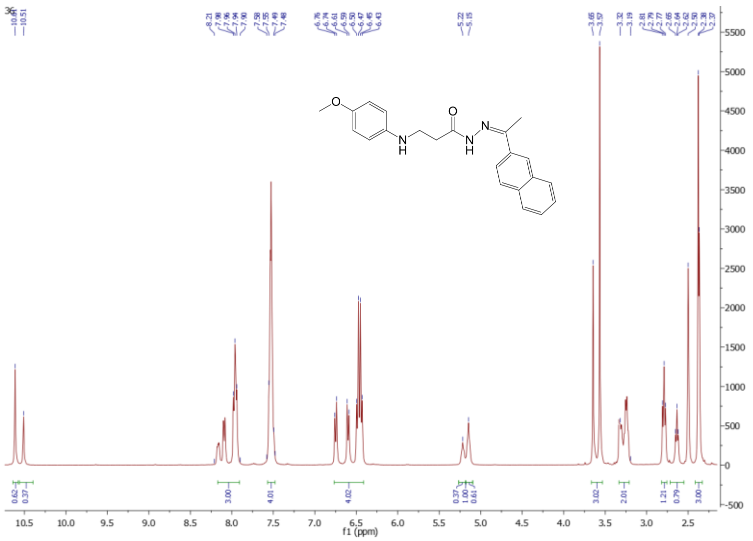

Figure S109. ^{1}H-NMR (400 MHz, DMSO-d_6) spectrum of **36**

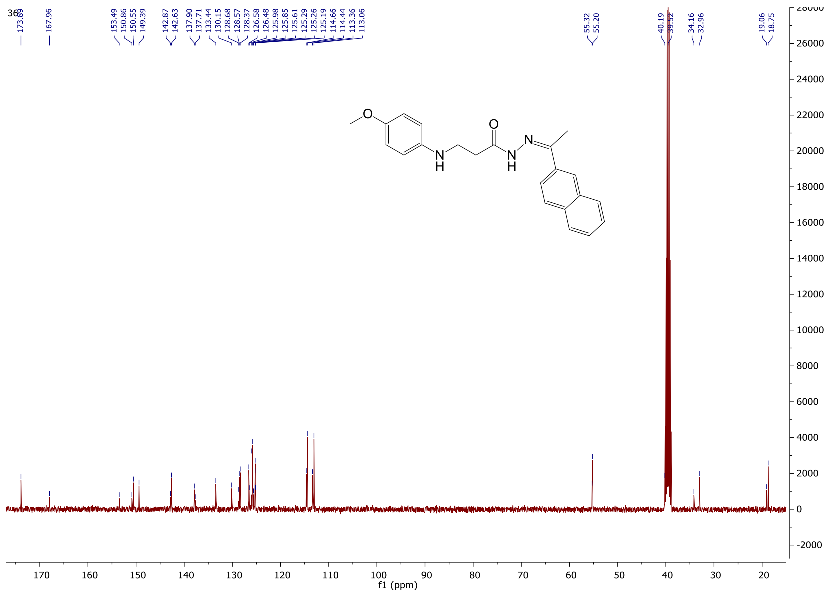

Figure S110. ^{13}C-NMR (400 MHz, DMSO-d_6) spectrum of **36**

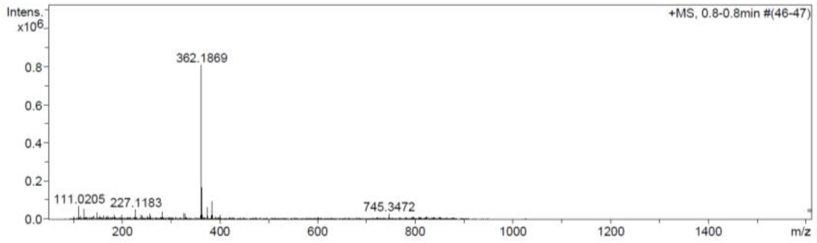

Meas. m/z	#	Formula	Score	m/z	err [ppm]	Mean err [ppm]	mSigma	rdb	e⁻ Conf	N-Rule
362.1869	1	C 20 H 22 N 6 O	49.60	362.1850	-5.4	-5.2	18.8	13.0	odd	ok
	2	C 22 H 24 N 3 O 2	100.00	362.1863	-1.7	-1.5	25.2	12.5	even	ok
	3	C 24 H 26 O 3	81.62	362.1876	2.0	2.2	31.7	12.0	odd	ok

Figure S111. HRMS spectrum of **36**

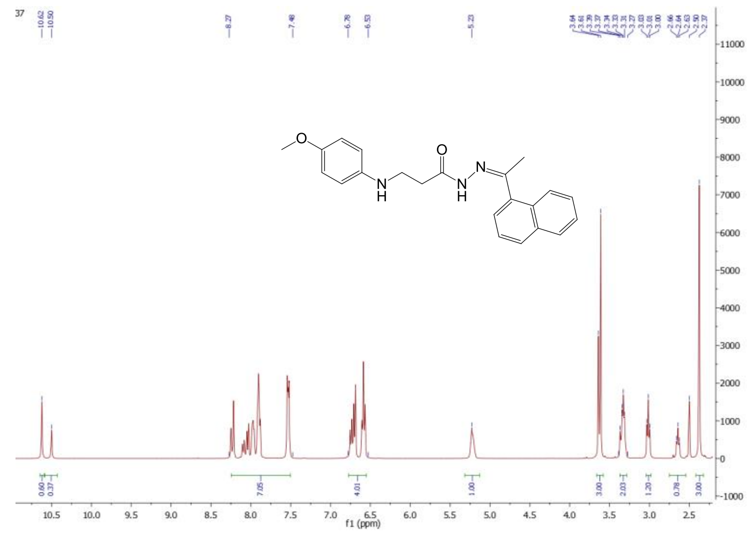

Figure S112. ^{1}H-NMR (400 MHz, DMSO-d_6) spectrum of **37**

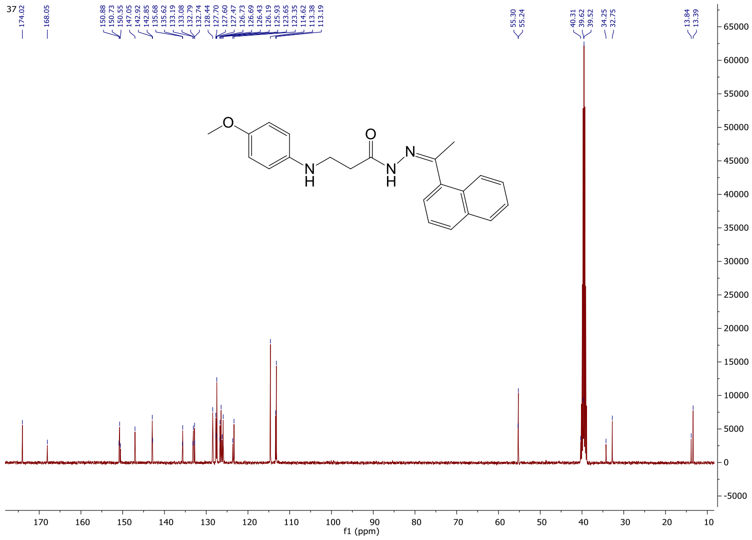

Figure S113. ^{13}C-NMR (400 MHz, DMSO-d_6) spectrum of **37**

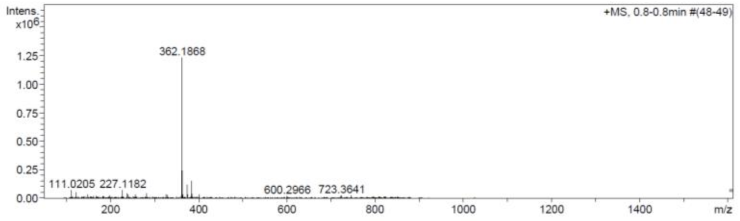

Meas. m/z	#	Formula	Score	m/z	err [ppm]	Mean err [ppm]	mSigma	rdb	e⁻ Conf	N-Rule
362.1868	1	C 20 H 22 N 6 O	51.32	362.1850	-5.2	-5.0	25.2	13.0	odd	ok
	2	C 22 H 24 N 3 O 2	100.00	362.1863	-1.5	-1.2	31.6	12.5	even	ok
	3	C 24 H 26 O 3	73.62	362.1876	2.2	2.5	38.0	12.0	odd	ok

Figure S114. HRMS spectrum of **37**

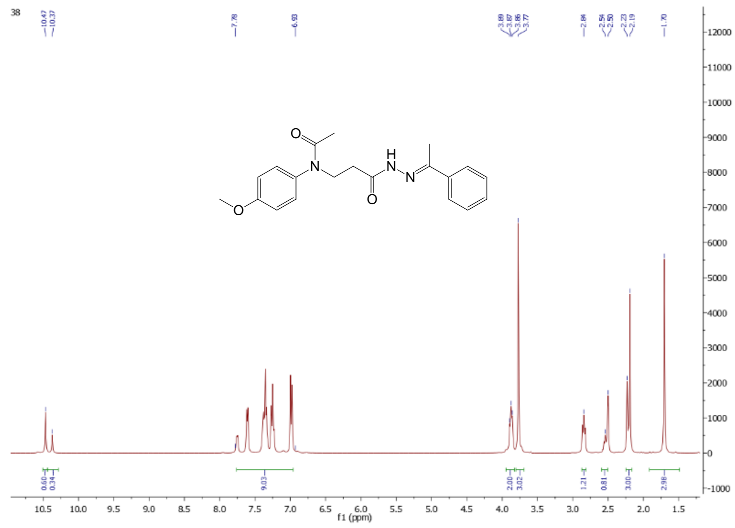

Figure S115. ^{1}H-NMR (400 MHz, DMSO-d_6) spectrum of **38**

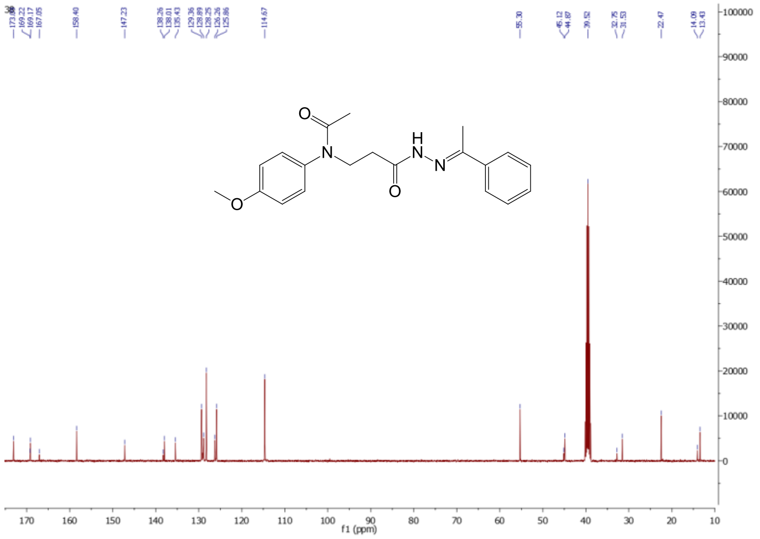

Figure S116. ^{13}C-NMR (400 MHz, DMSO-d_6) spectrum of **38**

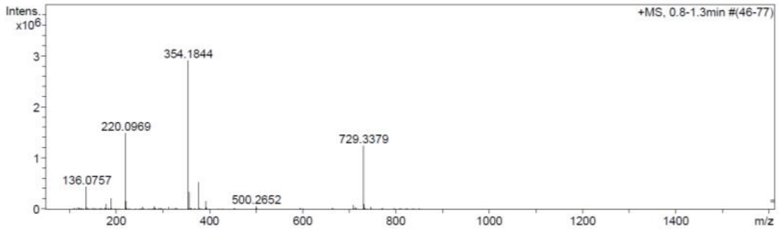

Meas. m/z	#	Formula	Score	m/z	err [ppm]	Mean err [ppm]	mSigma	rdb	e⁻ Conf	N-Rule
220.0969	1	C 10 H 12 N 4 O 2	55.87	220.0955	-6.4	-6.3	11.6	7.0	odd	ok
	2	C 12 H 14 N O 3	100.00	220.0968	-0.3	-0.2	17.9	6.5	even	ok
354.1844	1	C 25 H 24 N O	100.00	354.1852	2.3	5.9	286.9	14.5	even	ok
	2	C 23 H 22 N 4	54.60	354.1839	-1.5	2.0	293.2	15.0	odd	ok
	3	C 22 H 26 O 4	4.08	354.1826	-5.3	-1.7	307.6	10.0	odd	ok
	4	C 10 H 20 N 13 O 2	0.01	354.1857	3.7	7.0	356.1	7.5	even	ok
	5	C 8 H 18 N 16 O	0.01	354.1844	-0.1	2.1	363.0	8.0	odd	ok

Figure S117. HRMS spectrum of **38**

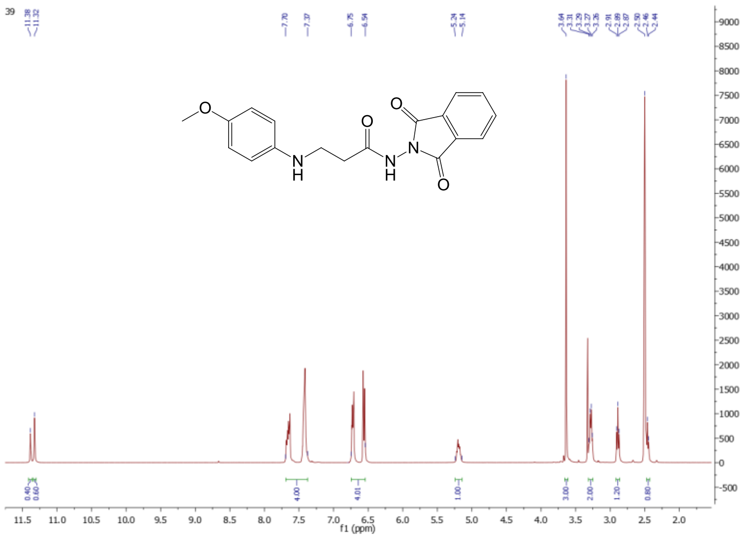

Figure S118. ^{1}H-NMR (400 MHz, DMSO-d_6) spectrum of **39**

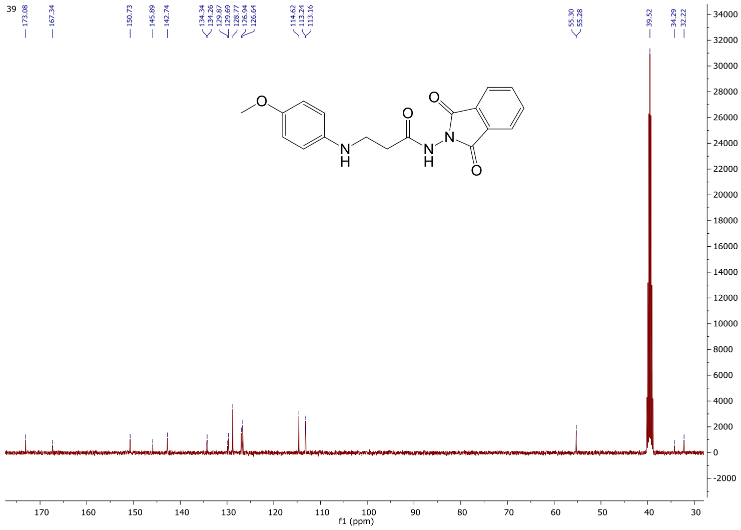

Figure S119. ^{13}C-NMR (400 MHz, DMSO-d_6) spectrum of **39**

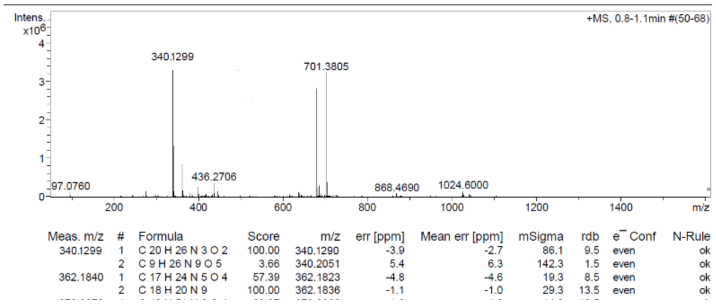

Figure S120. HRMS spectrum of **39**
